# Helical Bilayer Nanographenes: Impact of the Helicene Length on the Structural, Electrochemical, Photophysical, and Chiroptical Properties

**DOI:** 10.1021/jacs.3c01088

**Published:** 2023-05-02

**Authors:** Patricia Izquierdo-García, Jesús M. Fernández-García, Samara Medina Rivero, Michal Šámal, Jiří Rybáček, Lucie Bednárová, Sergio Ramírez-Barroso, Francisco J. Ramírez, Rafael Rodríguez, Josefina Perles, David García-Fresnadillo, Jeanne Crassous, Juan Casado, Irena G. Stará, Nazario Martín

**Affiliations:** † Departamento de Química Orgánica I, Facultad de Ciencias Químicas, Universidad Complutense de Madrid, 28040 Madrid, Spain; ‡ Departament of Physical Chemistry, Facultad de Ciencias, Universidad de Málaga, 29071 Málaga, Spain; § Department of Physics & Astronomy, University of Sheffield, S3 7RH Sheffield, U.K.; ∥ Laboratorio DRX Monocristal, SIdI, Universidad Autónoma de Madrid, 28049 Madrid, Spain; ⊥ Institut des Sciences Chimiques de Rennes (ISCR), UMR 6226 CNRS—Univ Rennes, 35000 Rennes, France; # Institute of Organic Chemistry and Biochemistry, Czech Academy of Sciences, Flemingovo nám. 2, 166 10 Prague 6, Czech Republic; ¶ IMDEA-Nanociencia, C/Faraday, 9, Campus de Cantoblanco, 28049 Madrid, Spain

## Abstract

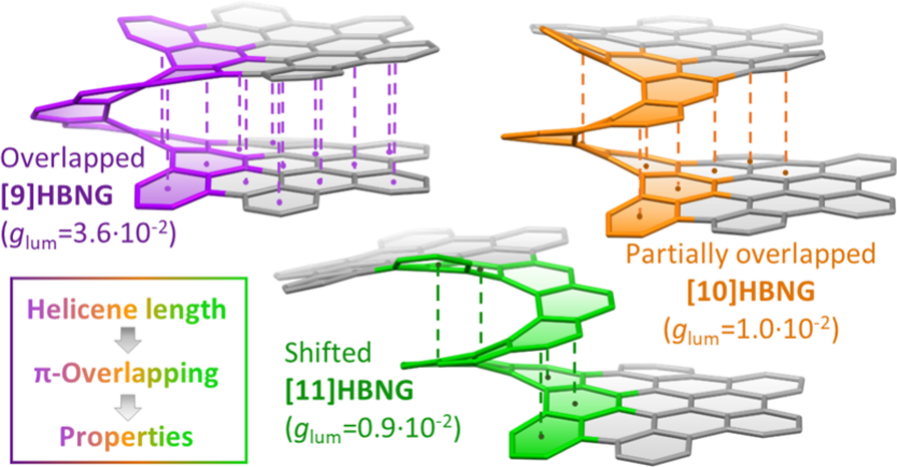

Helical bilayer nanographenes (HBNGs) are chiral π-extended aromatic compounds consisting of two π–π stacked hexabenzocoronenes (HBCs) joined by a helicene, thus resembling van der Waals layered 2D materials. Herein, we compare **[9]HBNG**, **[10]HBNG**, and **[11]HBNG** helical bilayers endowed with [9], [10], and [11]helicenes embedded in their structure, respectively. Interestingly, the helicene length defines the overlapping degree between the two HBCs (number of benzene rings involved in π–π interactions between the two layers), being 26, 14, and 10 benzene rings, respectively, according to the X-ray analysis. Unexpectedly, the electrochemical study shows that the lesser π-extended system **[9]HBNG** shows the strongest electron donor character, in part by interlayer exchange resonance, and more red-shifted values of emission. Furthermore, **[9]HBNG** also shows exceptional chiroptical properties with the biggest values of *g*_abs_ and *g*_lum_ (3.6 × 10^–2^) when compared to **[10]HBNG** and **[11]HBNG** owing to the fine alignment in the configuration of **[9]HBNG** between its electric and magnetic dipole transition moments. Furthermore, spectroelectrochemical studies as well as the fluorescence spectroscopy support the aforementioned experimental findings, thus confirming the strong impact of the helicene length on the properties of this new family of bilayer nanographenes.

## Introduction

The discovery of graphene by Geim and Novoselov in 2004 laid the foundations of the science of single-atom-thick layer materials or 2D materials.^1^ Since this historic milestone, the development of atomically thin layer materials—with carbon and other elements of the periodic table—has been successfully achieved along the last recent years. The new family of monoelemental 2D materials, namely, graphyne,^2^ borophene,^3^ germanene,^4^ silicene,^5^ stanene,^6^ plumbene,^7^ phosphorene,^8^ antimonene,^9^ and bismuthene,^10^ was rapidly followed by the search for multielemental layered materials.^11^ The main expectation is that, given their exceptional properties, 2D materials could replace conventional semiconductors to deliver a new generation of electronics with applications in sensors,^12^ low-cost organic solar cells,^13^ LEDs,^14^ transistors,^15^ piezoelectrics,^16^ superconductors,^17^ or magnetism,^18^ among others.

Chemically, the pristine forms of these materials show strong in-plane covalent bonds and weak out-of-plane van der Waals (vdW) interactions. The stacking of several layers generates pseudocrystalline materials known as vdW layered solids.^19^ Remarkably, the twist angle between the layers of stacked 2D materials produces a moiré pattern, forming a superlattice with a new band structure that dramatically affects the electronic properties of the material.^20^ In this regard, Jarillo-Herrero et al. have recently reported that a two-layered graphene having a “magic angle” of 1.1° between the two honeycomb lattices shows superconductivity at 1.7 K.^21^ This finding represents an important landmark in the search for high-temperature superconductivity.^22^

Interestingly, the band gap properties of 2D materials can also be modified by the quantum confinement of the electrons in dimensionally reduced structures (1D, nanoribbons; 0D, quantum dots).^23^ As a consequence of the edge effects in nanosized fragments of graphene, the so-called nanographenes (NGs), it is possible to open up the zero band gap of graphene, leading to semiconductor materials with potential applications in organic electronics.^24^

Additionally, the bottom-up synthesis of monodisperse molecular NGs by using the arsenal of organic chemistry reactions allows controlling at will the size, morphology, and the *ad hoc* incorporation of defects in the final structure of the molecule. This synthetic control allows, in turn, the fine-tuning of chemical, optical, and electronic properties.^25^ In the last recent years, a broad variety of molecular NGs have been synthesized by this methodology, covering a wide range of shapes, from the straightforward planar NGs^26^ to curved,^27^ twisted,^28^ or helically arranged molecular NGs.^29^ The incorporation of curvature, strain, or helicity in NGs may introduce asymmetry in the structures, leading to the appearance of stereoisomeric forms,^30^ that open the possibility of new applications derived from their chiroptical properties.^31^

**Chart 1:**
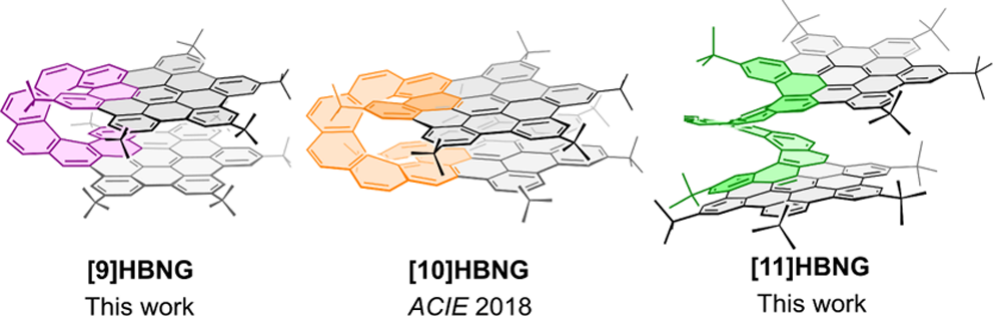
HBNGs Endowed with Helicenes of Different Length, which Results in Different Degrees of π-Overlapping between the HBC Layers

The combination of both, stacked vdW materials and quantum confinement of electrons in NGs, leads to a new type of molecular bilayer NGs whose properties remain basically unexplored and scarcely reported.^32^ In this regard, some of the co-authors pioneered the topic,^33^ and our research group has recently reported a helical bilayer nanographene (HBNG) **[10]HBNG** inspired by the π-extended connection of a [6]helicene moiety and two hexabenzocoronene (HBC) units (Chart 1).^34^ The length of the resulting [10]helicene places the HBC fragments in a AA stacking (AB for graphite) disposition.

Herein, we report on the synthesis of new molecular bilayer NGs starting from [5]helicene and [7]helicene.^35^ Both systems lead to the expected formation of the respective helical bilayers **[9]HBNG** and **[11]HBNG** (Chart 1). The different lengths of the helicene moiety ([9] and [11]helicenes, respectively) make the HBC layers to be disposed with a significantly different degree of overlapping between them. This allows us to address electronic features arising from the co-facial coupling such as mixed valence effects in the oxidized state, excimer-like formation in the excited state, and Raman vibrational properties comparable to graphene and multilayer graphene. Since the final bilayer NGs are obtained as racemates, a careful separation of their respective enantiomers has been performed by using chiral HPLC. Interestingly, the structural differences have a strong impact on their chiroptical, photophysical, electrochemical, and spectroelectrochemical properties, which are discussed in the light of the previously synthesized [10]helicene-containing bilayer **[10]HBNG**, whose new properties are also reported for the first time in this work. Singularly, **[9]HBNG** displays one of the largest chiral photoluminescence dissymmetry factors (*g*_lum_) reported for pure hydrocarbons. This experimental finding arises from an alignment of the electric and magnetic transition vectors, stemming from the fine balance between vertical and layered π-conjugations.

## Results and Discussion

**Scheme 1:**
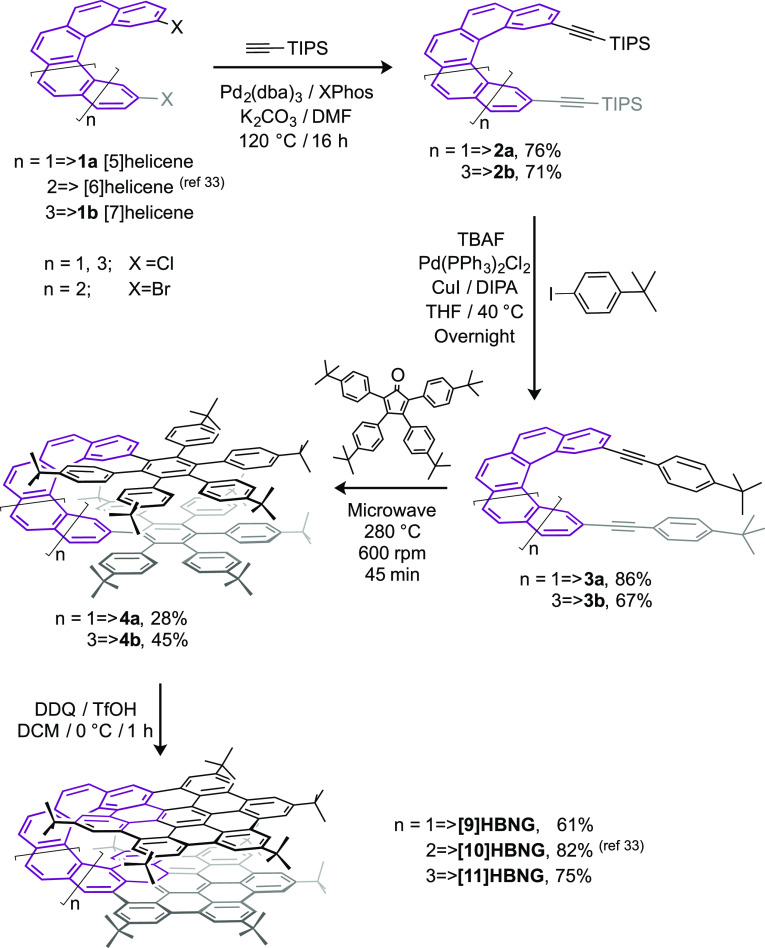
Synthesis of HBNGs **[9]HBNG** and **[11]HBNG** from Dichloro[5]helicene **1a** and Dichloro[7]helicene **1b**, Respectively

Inspired by the intriguing modification of the properties of 2D materials by twisting stacking bilayers, we decided to unveil the structural, chiroptical, photophysical, electrochemical, and spectroelectrochemical properties of this family of HBNGs. For this purpose, we designed a bottom-up synthetic strategy, in which starting from dihalo[n]helicenes with different lengths (dichloro[5]helicene **1a**(36) and dichloro[7]helicene **1b**(37)),^38^ it is possible to control the length of the resulting helicene in the final HBNG (**[9]HBNG** and **[11]HBNG**) and, therefore, the π-overlapping between the planar HBC layers (Scheme 1).

### Synthetic Procedure

The preparation of **[9]HBNG** and **[11]HBNG** was achieved using our previously reported synthetic strategy employed to prepare **[10]HBNG** (Scheme 1). These rapid syntheses consist of only four reaction steps starting from dichloro[5]helicene **1a** and dichloro[7]helicene **1b**. Pd-catalyzed Sonogashira cross-coupling reaction with (triisopropylsilyl)acetylene led to the protected dialkynylhelicenes **2a** and **2b**, respectively. Thereafter, a new Sonogashira reaction with 4-*tert*-butyliodobenzene, with in situ deprotection of the TIPS groups using TBAF, added a benzene ring to each triple bond affording **3a** and **3b**. Then, Diels–Alder cycloaddition of tetrakis-(4-*tert*-butylphenyl)cyclopentadienone to the alkynes, followed by the retrocheletropic liberation of carbon monoxide, led to the corresponding helicenes endowed with two pentaphenylbenzene groups **4a** and **4b**, respectively. Finally, Scholl cyclodehydrogenation using 2,3-dichloro-5,6-dicyano-1,4-benzoquinone and trifluoromethanesulfonic acid formed 12 new C–C bonds, shaping the two HBC layers and extending the helicene backbone from [5]- and [7]- to [9]- and [11]helicene, respectively, yielding the desired **[9]HBNG** and **[11]HBNG** compounds with good yields (61 and 75%, respectively). The final new molecules have been characterized by ^1^H and ^13^C NMR, Fourier-transform infrared, and high-resolution mass spectrometry. The loss of 24 units in the molecular weight of **4a** (MW = 1751) and **4b** (MW = 1851) when they afford the final products **[9]HBNG** (MW = 1727) and **[11]HBNG** (MW = 1827) clearly indicates the formation of 12 C–C bonds in the Scholl reaction step and reveals the total graphitization of the HBC moieties. Additionally, ^1^H and ^13^C NMR spectra only show the signals corresponding to a half of the molecule in both cases, revealing the presence of a *C*_2_ rotational axis (Figures S7 and S8, see the Supporting Information).

**Figure 1:**
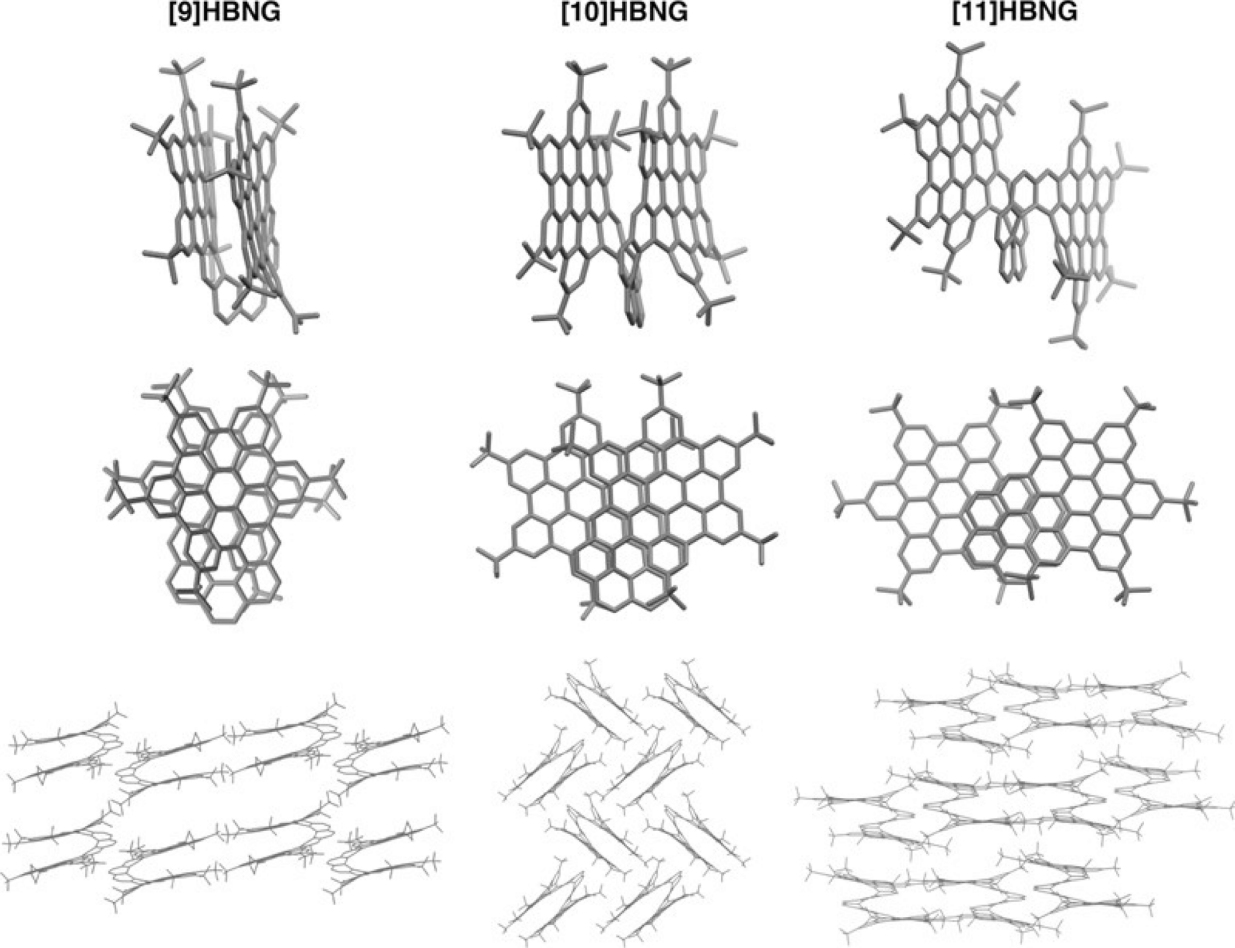
Molecular structures of compounds **[9]HBNG**, **[10]HBNG**,^34^ and **[11]HBNG** obtained by X-ray diffraction from single crystals (hydrogen atoms have been omitted for clarity). Lateral view (top), zenithal view (middle), and packing of the columns of molecules of **[9]HBNG** (along the [010] direction), **[10]HBNG** (along the [001]), and **[11]HBNG** (along [011]) (bottom).

The structures of **[9]HBNG** and **[11]HBNG** were solved by single-crystal X-ray diffraction (Figure 1). In both cases, centrosymmetric crystals containing *M* and *P* isomers were obtained from their respective racemic mixtures, unlike what happened in **[10]HBNG**, where the two isomers were spontaneously separated by crystallization, yielding enantiopure acentric crystals.^34^

The comparison of the three crystal structures reveals some interesting facts and explains the differences observed in their physical properties (see the sections below). As expected, the length of the helicene linker was found to be crucial in achieving a convenient location of the two HBC layers of the molecules and proved to influence the overlap of the two NG fragments.

Thus, a total of 26 benzene rings in **[9]HBNG** are involved in extended π–π interactions, resulting in an effectively through-space overlapped bilayer (i.e., see this effect in the zenithal view in Figure 1, middle left, and Figure S9, left). Consequently, the molecule adopts a remarkable U-shape from a side view (Figure 1, top), with a small interplanar angle of 2.37° between the layers of the HBC subunits and a distance between the pivotal atoms of opposite ^*t*^Bu groups of 5.185 Å (C45–C127). Due to geometrical constraints, the rings of the two layers are staggered, with an average distance of 3.63 Å between the centroids of one layer and the opposite plane (Figures S9 and S10 and Table S5, see the Supporting Information).

In **[10]HBNG**, the length of the helicene allows a partially overlapped AA-stacking of the two NG layers, with 14 rings participating in π–π interactions seen from a top view (Figure 1, middle, and Figure S9, middle). The similarity with an AA-type graphene stacking allowed the determination of an average distance of 3.54 Å between the matching centroids of opposite layers (Table S5, see the Supporting Information). The angle between the HBC planes has a value of 5.19°, giving a V-shape to **[10]HBNG** (Figure 1, top, and Figure S9, see the Supporting Information), while the distance between the opposing ^*t*^Bu groups is 12.436 Å (C99–C127) due to the skewed structure arising from the partial overlap between HBC subunits.

In the case of **[11]HBNG**, the extension of the helicene linker forces the two HBC layers to separate, resulting in a shifted bilayer that presents π–π interactions between 10 benzene rings (Figure S9, right). In this HBNG, only one of the peripheral rings from each layer can participate in these bonds, and the mean distance between centroids involved in π–π interactions is 3.41 Å. The length of the molecule is the largest of the three (C45–C131 = 23.368 Å), and it also displays the highest angle between the two HBC layers (11.51°), generating a structure with a distorted Z-shaped lateral view.

A consequence of the diverse shapes displayed by the three HBNGs is the different packing observed for these molecular NGs in their crystal structures (Figure 1, bottom). **[9]HBNG** molecules are closely located in pairs of *M* and *P* isomers (Figure S11, see the Supporting Information) due to the strong intermolecular π–π interactions (3.359 Å). Weak C–H···π interactions join the pairs of molecules, leaving huge voids, where disordered solvent molecules are lodged. Besides, columns of molecules are located at a perpendicular angle in the crystal structure of **[10]HBNG**, owing to C–H···π interactions. Finally, the packing of **[11]HBNG** is achieved by C–H···π supramolecular interactions not only between the HBNGs themselves but also between HBNGs and the interstitial dichloroethane molecules, yielding alternating homochiral columns in the [010] direction of *M* and *P* enantiomers (Figure S12).

### Electrochemical and Spectroelectrochemical Properties

**Figure 2:**
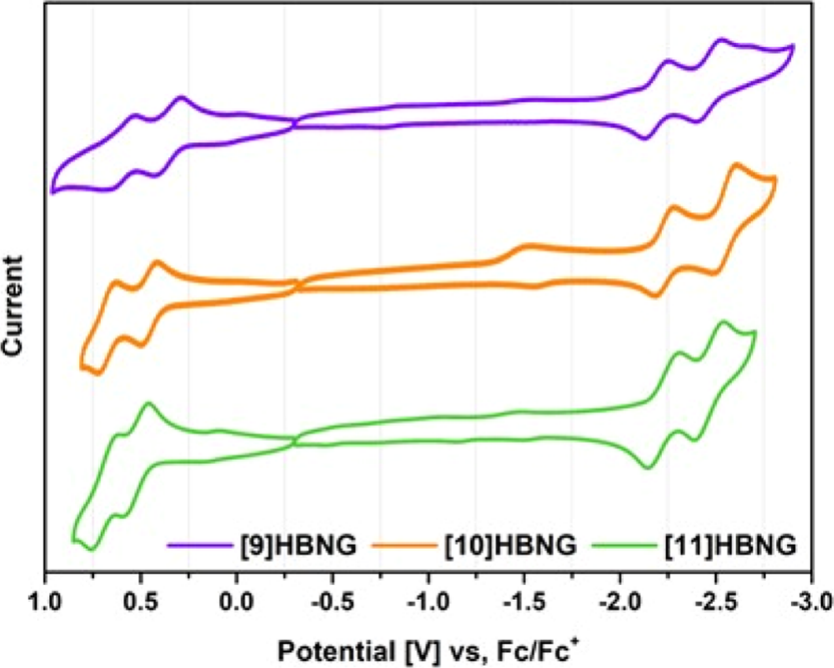
Cyclic voltammograms of HBNGs **[9]HBNG**, **[10]HBNG**, and **[11]HBNG** vs Fc/Fc^+^ in toluene/CH_3_CN (4:1).

The electrochemical properties of **[9]HBNG**, **[10]HBNG**, and **[11]HBNG** were evaluated by cyclic voltammetry (CV, Figure 2) in a 0.1 M solution of tetrabutylammonium hexafluorophosphate (i.e., Bu_4_NPF_6_) as supporting electrolyte, in a toluene/acetonitrile 4:1 mixture at room temperature. Table 1 shows the respective reduction and oxidation potentials vs Fc/Fc^+^ compared to hexa-*tert*-butylhexa-*peri*-hexabenzocoronene ^***t***^**Bu-HBC** as the reference.

**Table 1:** First Oxidation and Reduction Potential Values of ^***t***^**Bu-HBC** and Molecular NGs **[9]HBNG**, **[10]HBNG**, and **[11]HBNG** vs Fc/Fc^+^ at Room Temperature

	*E*_ox_^1^ (V)	*E*_ox_^2^ (V)	*E*_red_^1^ (V)	*E*_red_^2^ (V)
^***t***^**Bu-HBC**	0.75		–2.24	–2.40
**[9]HBNG**	0.35	0.59	–2.18	–2.46
**[10]HBNG**	0.46	0.67	–2.23	–2.55
**[11]HBNG**	0.52	0.69	–2.22	–2.46

In all three cases, two quasi-reversible oxidation waves and two quasi-reversible reduction waves are observed. The first oxidation potential follows the order **[9]HBNG** (0.35 V) < **[10]HBNG** (0.46 V) < **[11]HBNG** (0.52 V). Interestingly, the observed trend is the reverse of that expected according to the extension of the π-conjugation of the helicene moiety. Compared to ^***t***^**Bu-HBC** (0.75 V), these bilayer NGs are significantly better electron donors. The oxidation potential variation seems to indicate that the larger the π–π overlapping of the two layers, the lower the oxidation potential values. This experimental finding could be accounted for by the fact that the radical cation and the dication species (*E*_ox_^1^ and *E*_ox_^2^, respectively) are stabilized through the interaction between the two graphitized layers. This observation agrees with the location of the HOMO on the bilayer moieties.^34^ Regarding the reduction potentials, previous studies on the **[10]HBNG** bilayer show that the reduction occurs, mainly, on the helicene region where the LUMO is located,^29d^ and reduction waves at −2.18, −2.23, and −2.22 V are observed for **[9]HBNG**, **[10]HBNG**, and **[11]HBNG**, respectively.

Density functional theory calculations of the HOMO and LUMO topologies and energies have been carried out and are shown in Figure S26. A nice correlation is observed in the values of the HOMO and LUMO energies with the variation of the experimental oxidation and reduction potentials. In particular, the LUMOs of the three compounds are mainly located in the helicene moiety. In this regard, it is the scarce through bond conjugation along the helicene on the three compounds that justifies the small changes observed in their reduction potentials. On the other hand, the HOMOs are mainly located in the HBC units, and their energies vary in line with the measured oxidation potentials. Nonetheless, in order to account for the quantitative changes, through-space π-delocalization between the two layers in the radical cation needs to be considered.

**Figure 3:**
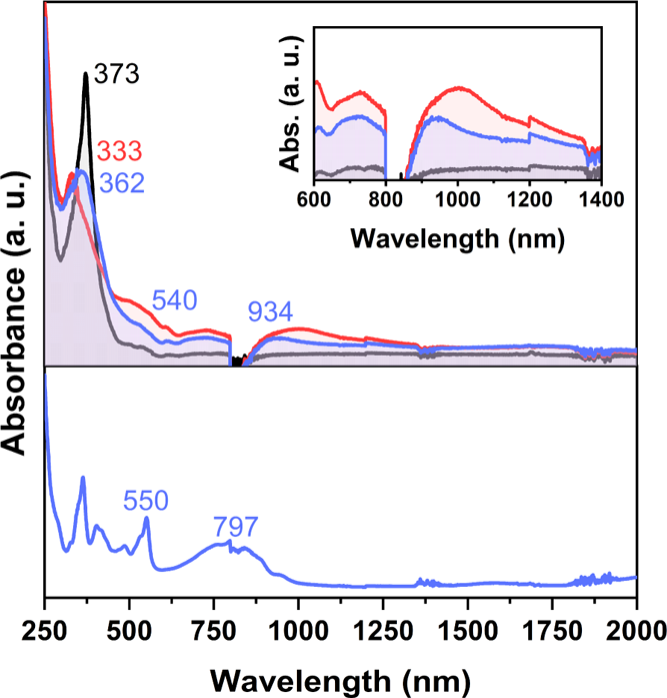
UV–vis–NIR electronic absorption spectra obtained upon electrochemical oxidation of **[9]HBNG** in a 0.1 M Bu_4_NPF_6_ solution in CH_2_Cl_2_ at room temperature by using a thin-layer spectroelectrochemical cell. Black shadowed lines correspond to neutral species; blue/red shadowed lines correspond to the first/second oxidized species. UV–vis–NIR electronic absorption spectrum of the ^***t***^**Bu-HBC** radical cation is also shown (bottom, blue line) for reference.

UV–vis–NIR spectroelectrochemical measurements of the three compounds have been carried out in a thin-layer spectroelectrochemical cell (Figures 3 and S17, see the Supporting Information). The first oxidation of **[9]HBNG** leads to the appearance of two distinctive absorption bands at 934 and 540 nm that compare with those of the radical cation of ^***t***^**Bu-HBC** at 797 and 550 nm (Figure 3, bottom, and Figure S17a, see the Supporting Information). The observed 797 → 934 nm red shift agrees with the smaller oxidation potential of **[9]HBNG** relative to ^***t***^**Bu-HBC**. The significant decrease of the oxidation potential observed for **[9]HBNG** might reveal a twofold effect: (i) the involvement of the helicene moiety in the oxidation and (ii) based on the previous wavelength red shift of the absorption bands, an inter-layer charge delocalization, which eventually contributes to lower the oxidation potential.

In the case of the second oxidation process, which is only observed in **[9]HBNG** (i.e., not in ^***t***^**Bu-HBC**), this further oxidation provokes a blue shift of the main UV–vis–NIR absorption bands, in line with the confinement of one radical cation in each HBC moiety due to charge repulsion in the dication state. The strongest absorption band of the neutral at 373 nm in **[9]HBNG** progressively blue-shifts on oxidation to 362 and 333 nm in the first and second oxidized species, respectively (Figure 3). Given that the strong band in the neutral state is composed of transitions emerging from the helicene and the HBC moieties, the notable variation of this band with oxidation supports that, upon electron extraction, the charge is jointly stabilized by the two moieties.

The electronic absorption spectra of **[11]HBNG** in the two oxidation states display a progressive red shift with increasing oxidation for the vis-NIR bands, in accordance with the charge being stabilized in a similar way in the two redox species of **[11]HBNG**.

### Photophysical Properties of the Racemic Mixture

**Figure 4:**
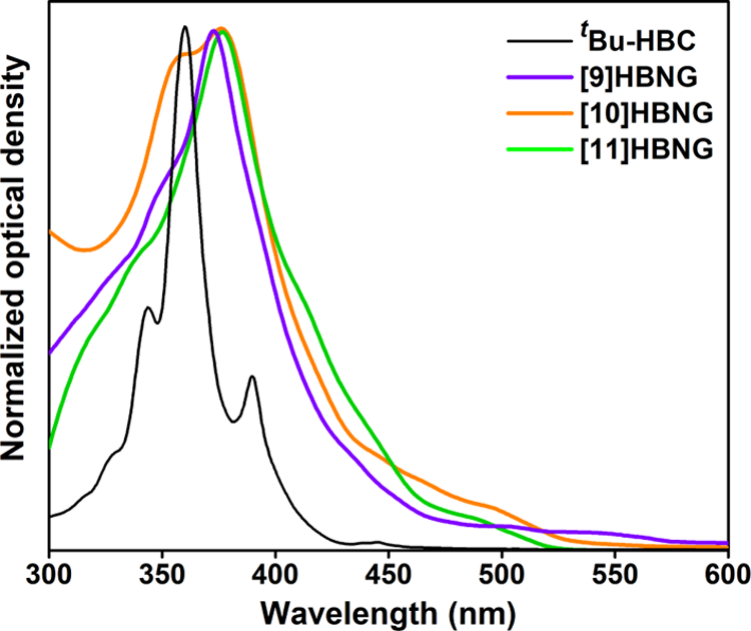
Absorption spectra of ^***t***^**Bu-HBC** and of HBNGs **[9]HBNG**, **[10]HBNG**, and **[11]HBNG** in CHCl_3_.

The absorption spectra of the HBNGs **[9]HBNG** (overlapped bilayer), **[10]HBNG** (partially overlapped), and **[11]HBNG** (shifted bilayer) and of the monolayer NG, *tert*-butylated hexabenzocoronene, ^***t***^**Bu-HBC**, are shown in Figure 4. Their corresponding peak/shoulder wavelengths and absorption coefficients are shown in Table 2. Shoulders were determined from the second derivative of the absorption spectrum. The rigid and flat ^***t***^**Bu-HBC** monolayer NG shows a structured absorption spectrum with a vibronic fine structure and a maximum at 360 nm, while the HBNGs **[9]HBNG**, **[10]HBNG**, and **[11]HBNG** display non-structured and broader absorption spectra, extended to 569, 557, and 518 nm, respectively (absorption tails with at least 1% absorption with respect to that of the maximum), with slightly bathochromically shifted maxima in the 373–377 nm interval. Interestingly, the HBNGs show allowed electronic transitions in the visible region above 400 nm, with absorption coefficients >10^4^ M^–1^ cm^–1^. Conversely, the planar ^***t***^**Bu-HBC** monolayer NG exhibits forbidden transitions in the visible region (ε ∼ 10^3^ M^–1^ cm^–1^). Therefore, the more intense and red-shifted absorption bands observed in the visible region reflect the direct involvement of both helicene and HBC moieties in the optical properties of HBNGs, in good agreement with the previous literature on curved molecular NGs containing both subunits.^27c^

**Table 2:** Data from Absorption Spectra of the HBNGs **[9]HBNG**, **[10]HBNG**, and **[11]HBNG** and of the Monolayer NG ^***t***^**Bu-HBC** in Chloroform

	λ_abs_/nm (ε/M^–^^1^cm^–^^1^)^a^
**[9]HBNG**	349 (94,600); **373** (136,000); 392 (86,300); 435 (20,600); 502 (6200); and 542 (4700)
**[10]HBNG**	360 (98,600); **377** (103,900); 415 (38,800); 446 (18,700); 462 (14,400); and 494 (8700)
**[11]HBNG**	339 (70,800); **377** (125,000); 415 (58,200); 444 (26,100); and 491 (7300)
^***t***^**Bu-HBC**	344 (63,750); **360** (141,700); 390 (46,350); 439 (1600); 441 (1650); and 445 (1800)

a λ_abs_/nm (±1 nm) (ε/M^–1^ cm^–1^) (±10%).

**Figure 5:**
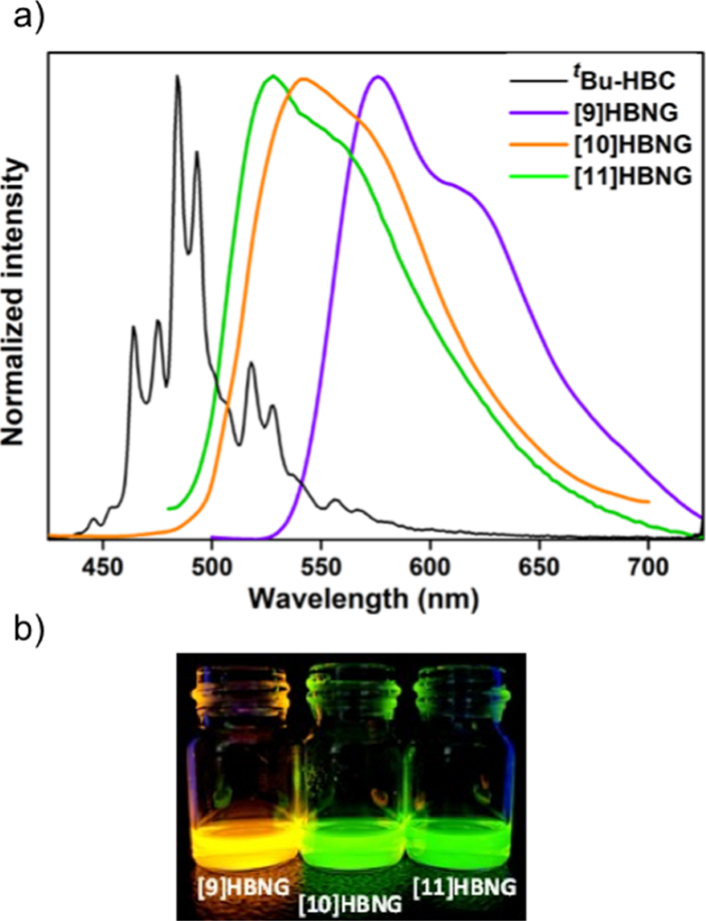
(a) Fluorescence spectra of ^***t***^**Bu-HBC** and of HBNGs **[9]HBNG**, **[10]HBNG**, and **[11]HBNG** in CHCl_3_. (b) Samples of **[9]HBNG**, **[10]HBNG**, and **[11]HBNG** in CHCl_3_ under 365 nm lamp irradiation (bottom).

Regarding the fluorescence of the HBNGs, their corresponding emission spectra are shown in Figure 5. Compared to ^***t***^**Bu-HBC**, they show broader, non-structured, and red-shifted emission bands, with full width at half-maximum (fwhm) values in the ∼2500–3000 cm^–1^ interval (cf. ∼1600 cm^–1^ for ^***t***^**Bu-HBC**). The emission spectra have two main features: (i) in shape, they resemble more the emission spectra of helicenes with one molecular band disclosing vibronic resolution and (ii) the pronounced broadening is more typical of excimer-type emissions. According to (ii), the most red-shifted excimer-like band is for **[9]HBNG**, in line with the greater propensity to form interlayer interactions. According to (i), the three emissions have clear helicene contributions. Remarkably, **[9]HBNG** (overlapped bilayer), the bilayer helicene with the lowest number of fused benzene rings (9) and most overlapped π-structure (i.e., larger π–π interactions), displays the most bathochromically shifted fluorescence (pointing to a more stable singlet excited state as compared to **[10]HBNG** and **[11]HBNG**). It is likely that structural differences among the bilayer NGs considering the increasing number of benzene rings within the helicene framework, at the level of their torsional angle or their interplanar angle, resulting in different interlayer interactions, might influence the distinct fluorescence shifts and, therefore, the general photophysics of the HBNGs. Emission data of the monolayer NG ^***t***^**Bu-HBC** and of the HBNGs **[9]HBNG**, **[10]HBNG**, and **[11]HBNG** are shown in Table 3. The Stokes’ shifts of the bilayer NGs are 9419 cm^–1^ (204 nm) for **[9]HBNG**, 8109 cm^–1^ (187 nm) for **[10]HBNG**, and 7585 cm^–1^ (154 nm) for **[11]HBNG**, while the planar and more rigid monolayer NG ^***t***^**Bu-HBC** shows the lowest Stokes’ shift (7117 cm^–1^, 124 nm). This result agrees with the lower molecular stiffness of the bilayer NGs studied and, notably, points to the higher singlet excited state reorganization energy of **[9]HBNG** compared to **[10]HBNG** and, in particular, **[11]HBNG** (the one with the lowest degree of overlap).

**Table 3:** Data from the Excitation and Fluorescence Spectra of the HBNGs **[9]HBNG**, **[10]HBNG**, and **[11]HBNG** and of the Monolayer NG ^***t***^**Bu-HBC** in Chloroform

	λ_em_^max^/nm (shoulder)	fwhm/cm^–^^1^ (eV)	Stokes’ shift/cm^–^^1^ (eV)	*E*_0–0_/eV	Φ_em_
**[9]HBNG**	575 (611)	2547 (0.32)	9419 (1.17)	2.35 ± 0.09	0.22 ± 0.02
**[10]HBNG**	543 (567)	2943 (0.36)	8109 (1.00)	2.49 ± 0.09	0.10 ± 0.01
**[11]HBNG**	528 (551)	3012 (0.37)	7585 (0.94)	2.58 ± 0.09	0.11 ± 0.01
^***t***^**Bu-HBC**	445, 454, 464, 475, 484, 493, 518, 528, (537), 556, and 567	1613 (0.20)^27c^	7117 (0.88)	2.65 ± 0.13^27c,39^	0.02 ± 0.01

The estimated optical energy gaps were obtained from the intersections between the normalized excitation and emission spectra of the HBNGs **[9]HBNG**, **[10]HBNG**, and **[11]HBNG**, as shown in Table 3. The values found are between 2.3 and 2.6 eV. The fluorescence quantum yields (Φ_em_) of **[9]HBNG**, **[10]HBNG**, and **[11]HBNG** (0.22, 0.10, and 0.11, respectively) were determined at room temperature using N_2_-purged solutions with optical densities <0.1 at 395 nm, by comparison with the emission spectra of riboflavin in ethanol (Φ_em_ = 0.30 ± 0.03). Φ_em_ variation of the three HBNGs does not follow the energy gap law; however, they can be accounted for, in part, by the increasing contribution of the excimer-like emission which enlarges the fluorescence quantum yield in **[9]HBNG** despite having the smallest optical gap.

Regarding the time-resolved emission of HBNGs **[9]HBNG**, **[10]HBNG**, and **[11]HBNG**, fluorescence decay profiles in chloroform at room temperature are shown in Figure S18, see the Supporting Information. While **[9]HBNG** and **[10]HBNG**, the HBNGs with strongly and partially overlapped bilayers, required multiexponential fittings of the decay function, **[11]HBNG**, the one with a shifted bilayer, exhibited a monoexponential behavior. The discrete lifetime components (τ_i_), pre-exponential factors (*B*_i_), zero-time intensities (*I*_i_), and averaged lifetimes (intensity-weighted, τ_Int_, or amplitude-weighted, τ_Amp_) are shown in Table S6, see the Supporting Information. The intensity-weighted average lifetimes of **[9]HBNG** (13.4 ns) and **[10]HBNG** (12.2 ns) determined at the wavelength of the emission intensity maximum display longer lifetimes than **[11]HBNG** (8.7 ns), which exhibits the most blue-shifted emission among the HBNGs, according to the less stable nature of its singlet excited state of higher energy.

From the amplitude-weighted fluorescence average lifetimes and fluorescence quantum yields, the radiative (*k*_r_) and non-radiative (*k*_nr_) deactivation rate constants of the respective singlet excited states were calculated (Table S7, see the Supporting Information).^40^ Interestingly, while all the *k*_r_ values are rather similar among the studied HBNGs (ca 1–2 × 10^7^ s^–1^), **[10]HBNG** and **[11]HBNG**, the molecular NGs with less bilayer overlaps, show the largest *k*_nr_ values (1–1.5 × 10^8^ s^–1^), which could be due to their higher structural mobility because of their less overlapped nature compared to their more π-stacked and constrained counterpart **[9]HBNG**. As a result, it could be concluded that the greater the structural overlap between NG layers, the more the relative radiative deactivation is favored, leading to higher emission quantum yields and longer fluorescence lifetimes.

**Figure 6:**
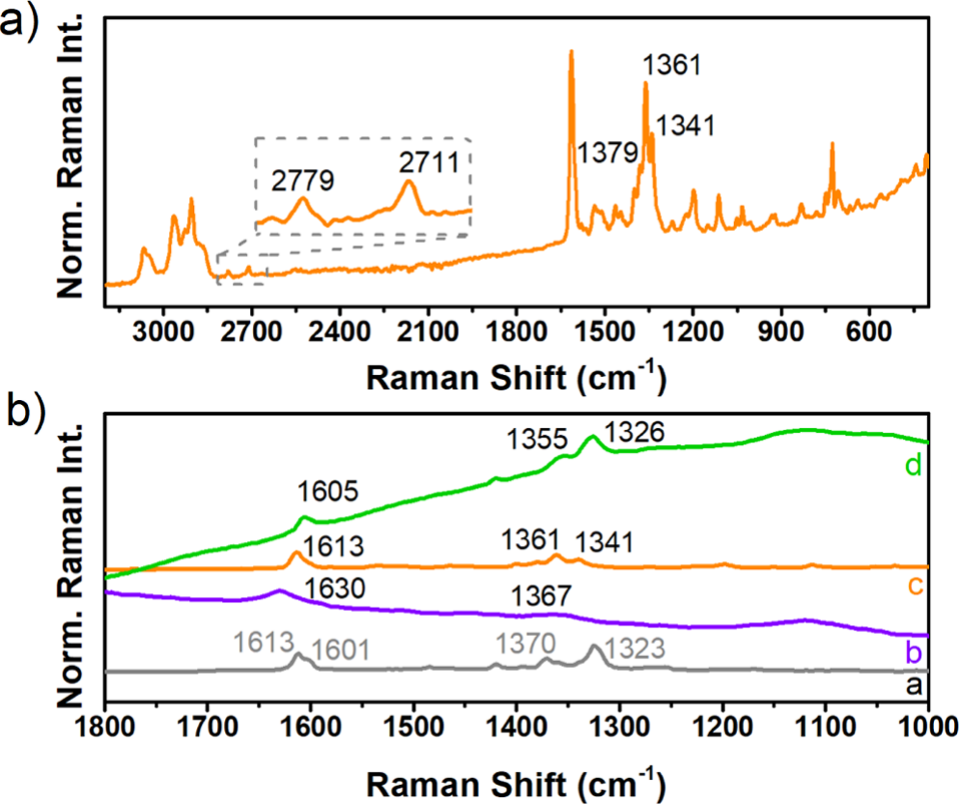
(a) Solid-state Raman spectrum at room temperature of **[10]HBNG** (λ_exc_ = 1064 nm). (b) Raman spectra of (a) ^***t***^**Bu-HBC** (λ_exc_ = 1064 nm), (b) **[9]HBNG** (λ_exc_ = 325 nm), (c) **[10]HBNG** (λ_exc_ = 1064 nm), and (d) **[11]HBNG** in the solid state at room temperature.

The Raman spectra of the three bilayer compounds (Figure 6) have been measured and obtained in the solid state by using different excitation laser wavelengths to avoid the adverse effect of fluorescence. Indeed, for **[10]HBNG** and **[11]HBNG**, the 1064 nm Raman spectra were obtained, whereas for **[9]HBNG**, large fluorescence and reabsorption in the NIR region precludes to obtain Raman spectra of quality. In contrast, however, these were obtained in **[9]HBNG** by excitation in the UV region with the 325 nm laser Raman line. The Raman spectra are all characterized by the presence of two main groups of bands at 1600 and 1350–1300 cm^–1^ which are the typical G and D Raman bands which feature the vibrational Raman spectra of graphene and graphitic-like systems (see Figure S19 and Figure S20 in the Supporting Information for the theoretical Raman spectra and assignments of the most representative Raman bands in terms of vibrational normal modes). In particular, the 1064 nm Raman spectrum of **[10]HBNG** represents the molecular version of the complete Raman spectrum of graphene and multilayer graphene. The packing of graphene layers, one on top of the other, leads to the activation and appearance in the Raman spectra of new bands associated with the double and triple wavenumbers of the fundamental phonon Raman vibrations. The occurrence of such multiphonon (i.e., 2D and 2G) bands is an intrinsic characteristic observed on passing from graphene to multilayer graphene. In the Raman spectrum of **[10]HBNG**, the presence of overtones and combination bands is clearly observed, which are the molecular equivalents of the multiphonon Raman bands in multilayered graphene.

Among the Raman spectra of the three bilayer compounds, that of **[10]HBNG** shows the largest similarity with that of ^***t***^**Bu-HBC** in the wavenumber region of the G Raman band at 1613 cm^–1^, whereas the G band of **[9]HBNG** at 1630 cm^–1^ is that with the largest difference relative to ^***t***^**Bu-HBC**. The variation 1613 (^***t***^**Bu-HBC**) → 1630 (**[9]HBNG**) cm^–1^ can result by the vibrational mixing of the C–C bond stretching of the HBC moiety with that of the helicene (Figure S20), an effect that might account for the wavenumber upshift of these Raman bands.

### Chiroptical Properties of Enantioenriched HBNGs

Racemic HBNGs **[9]HBNG**, **[10]HBNG**, and **[11]HBNG** were resolved by means of semipreparative CSP HPLC using the Chiralpak IE column and a mixture of heptane and 1% isopropyl alcohol in toluene. The purity and optical rotation of the separated enantiomers of **[9]HBNG**, **[10]HBNG**, and **[11]HBNG** are shown in Table 4. All enantioenriched samples show excellent enantiomeric excess (ee) except for the case of **(+)-[10]HBNG** that shows 73% ee. Additionally, the chiroptical circular dichroism for the six enantioenriched samples was measured in THF (2.5 × 10^–5^ M).

**Table 4:** Data from the Separation of the Racemic HBNGs by HPLC

	OR^a^ [α]_D_^20^	10^2^ × *g*_abs_	HPLC ee (%)
**(+)-[9]HBNG**	+6706	+3.6	98
**(−)-[9]HBNG**	–7120	–2.8	99
**(+)-[10]HBNG**	+2447	+1.4^b^	73
**(−)-[10]HBNG**	–4480	–1.6	99
**(+)-[11]HBNG**	+4033	+1.0	99
**(−)-[11]HBNG**	–4270	–1.0	99

a Optical rotation in THF. In addition to the loss of the material during chromatography, especially in the case of **[10]HBNG**, there were residual solvents present in the resolved samples, mainly toluene and/or dichloromethane (Section 9, see the Supporting Information). The larger discrepancy in the absolute values of the optical rotations of the opposite enantiomers is due to the presence of residual solvents after HPLC separation, see the Supporting Information for details.

b Corrected to optical purity (i.e., scaled by a factor of ∼1.4).

**Figure 7:**
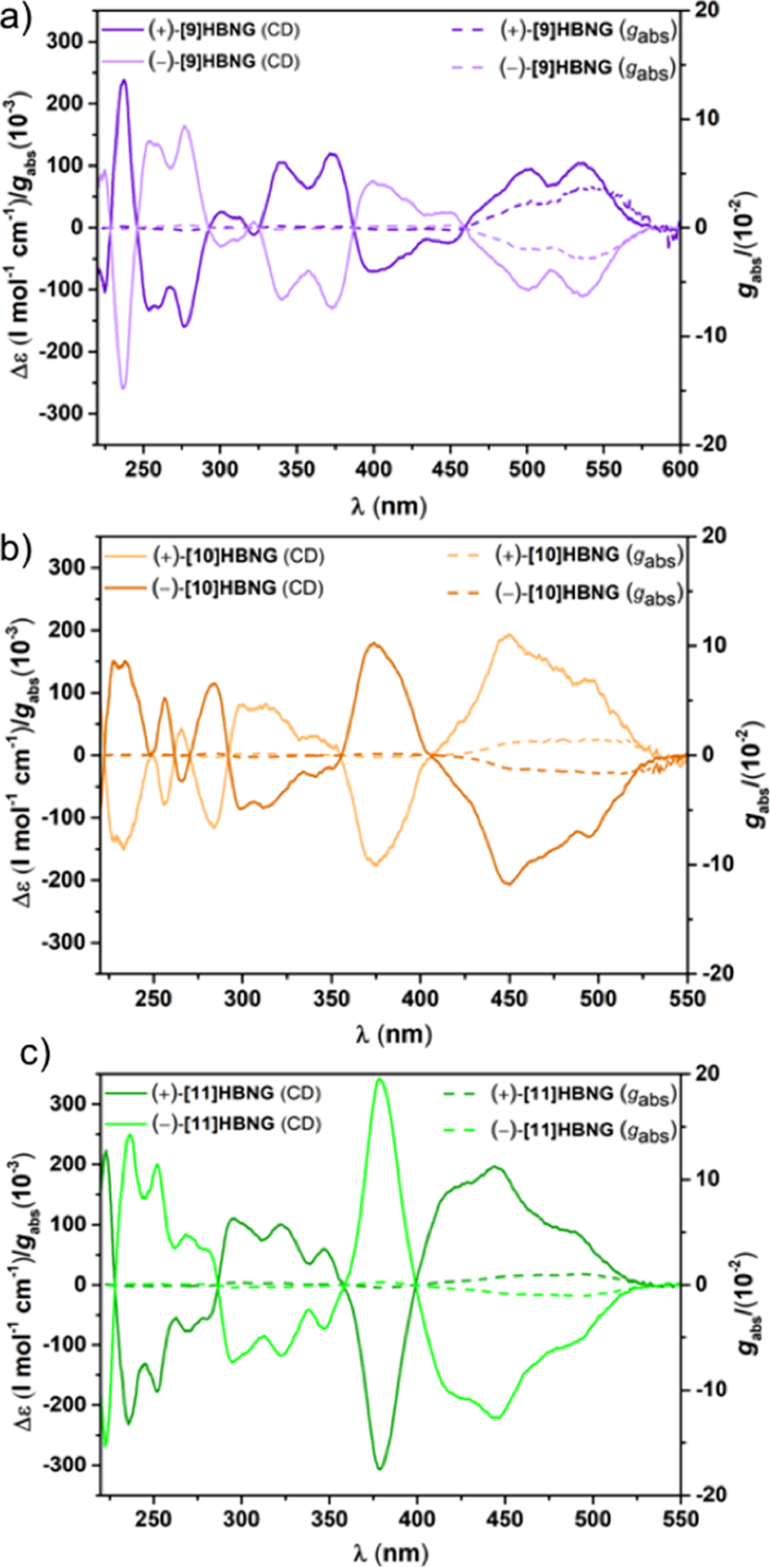
Circular dichroism spectra of **(+)-[9]HBNG** and **(−)-[9]HBNG** (a), **(+)-[10]HBNG** and **(−)-[10]HBNG** (b), and **(+)-[11]HBNG** and **(−)-[11]HBNG** (c). The CD spectrum of **(+)-[10]HBNG** (including *g*_abs_ factor) is corrected to optical purity (i.e., scaled by a factor of ∼1.4).

The CD spectra of **[10]HBNG** and **[11]HBNG** show mainly two critical points in the visible region at 380 and 450 nm, arising from the helicene linker and the HBC layers, respectively (Figure 7). However, CD spectra of **[9]HBNG** show three maxima or minima in the same region at 380, 400, and 530 nm; in this case, the contributions of the helicene linker and the HBC are mixed, indicating that the total overlap of the HBC layers can play an important role in the chiroptical properties (see below). Comparing the CD spectra of **[10]HBNG** with the previously described spectrum of ***M*-[10]HBNG**,^34^ the absolute configuration of both enantiomers of **[10]HBNG** can be assigned as (*P*) for the dextrorotatory enantiomer and (*M*) for the levorotatory enantiomer.

Note that the absorption dissymmetry factors (*g*_abs_) are quite high (1–3.6 × 10^–2^) and of the same order as the *g*_lum_ values (see below), reflecting small structural and electronic reorganization of the excited state prior to the emission process. In addition to the optical rotation and circular dichroism of chiral molecules, a particular focus has recently been put on their circularly polarized emission (CPL).^41^ Helicenes have shown to display rather strong CPL activity with emission dissymmetry factor (i.e., *g*_lum_) values as high as 10^–2^.^42^ Recently, the possibility to incorporate heteroatoms (Si, S, B, N, and P) into helicene frameworks by using different synthetic strategies has attracted a special attention with the aim of creating structural diversity and tuning the photophysical properties of such systems.^43^ However, CPL-active all-carbon PAHs are still scarce, and the preparation of PAHs with strong CPL activity is still challenging.^44^

**Figure 8:**
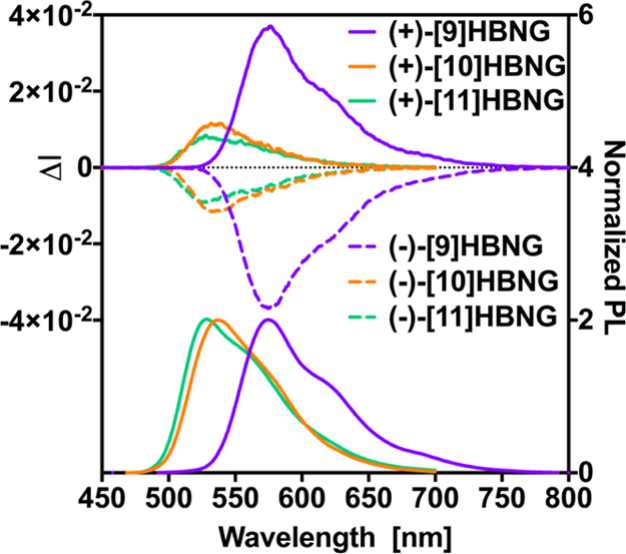
CPL/PL spectra of (*P*) and (*M*) enantiomers of **[9]HBNG**, **[10]HBNG**, and **[11]HBNG** in THF at room temperature and at concentrations around 10^–5^ M.

Interestingly, **[10]HBNG** and **[11]HBNG** exhibit intense CPL spectra, positive for the (*P*) enantiomers and negative for the (*M*) ones. Remarkable *g*_lum_ values (i.e., *g*_lum_ = 2(*I*_L_ – *I*_R_)/(*I*_L_ + *I*_R_), *I*_L_ and *I*_R_ being the left- and right-handed luminescent emissions, respectively) were obtained. Indeed, *g*_lum_ values of +1.1 × 10^–2^/–1 × 10^–2^ at 540 nm for (*P*)-**[10]HBNG** and (*M*)-**[10]HBNG** and of +8.4 × 10^–3^/–8.9 × 10^–3^ at 535 nm for (*P*)-**[11]HBNG** and (*M*)-**[11]HBNG** were measured, respectively. Note that higher absolute values were found for the (*M*) enantiomers as compared to the (*P*) ones, probably due to higher chemical and enantiomeric purities. Remarkably, much higher *g*_lum_ values were obtained for (*P*)-**[9]HBNG** and (*M*)-**[9]HBNG**, that is, +3.6 × 10^–2^/–3.6 × 10^–2^ at 580 nm, respectively (Figure 8).

The experimental values of *g*_lum_ agree very well with the theoretical values in Table 5 calculated from the quantum chemical quantities, such as the electric transition dipole moment (ETDM, μ) and the magnetic transition dipole moments (MTDM, *m*) according to the equations
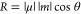

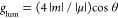


**Table 5:** TD-DFT/B3LYP/6-31G** Quantum Chemical Calculations of the Relevant Quantities Defining the *g*_abs_ for the Three Compounds

	wavelength^a^	oscillator strength (*f*)^b^	ETDM (μ) module^c^	MTDM (*m*) module^d^	ETDM/MTDM angle (θ)^e^	rotational strength (*R*)^f^	10^2^ × dissymmetry factor (*g*_lum_)^g^
**[9]HBNG**	406.6	0.0771	1.0160	2.6270	3.4	2.664·× 10^–^^39^	1.03
**[10]HBNG**	404.4	0.0671	0.9453	1.4014	17.78	1.261·× 10^–^^39^	0.56
**[11]HBNG**	389.5	0.0548	0.8385	1.7485	63.51	0.654·× 10^–^^39^	0.37

a In nanometers.

b In atomic units.

c Electric transition dipole moments in 10^–18^ esu·cm.

d Magnetic transition dipole moments in 10^–21^ erg/Gauss.

e In degrees.

f In erg esu cm/Gauss.

g Dimensionless values.

**Figure 9:**
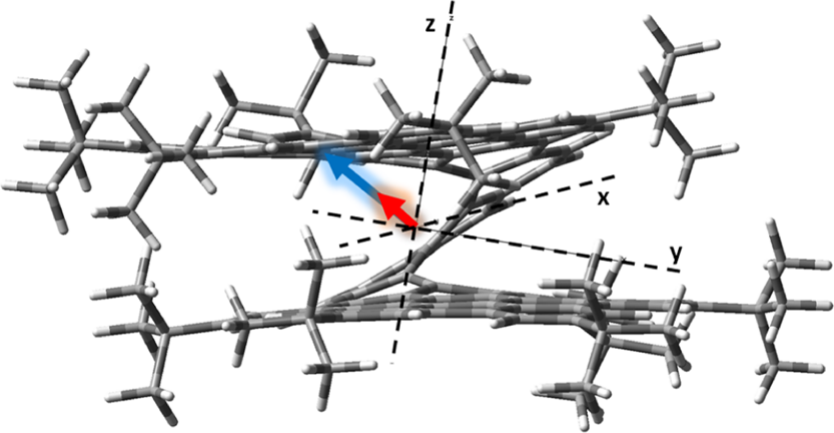
Directions and moduli of the ETDM (blue arrow) and MTDM (red arrows) in **[9]HBNG**.

Table 5 summarizes the main values obtained with TD-DFT quantum chemical calculations for the three compounds. We adopt the accepted approximation of discussing the experimental *g*_lum_ compared with the computationally available *g*_abs_ when emission and absorption correspond to the same lowest energy electronic transition. The dissection of the *g*_abs_ values in terms of the microscopic quantum chemical quantities allows us to provide guidelines to account for the different chiroptical performance of the three compounds. First, TD-DFT calculations nicely predict the experimental variation of the *g*_lum_ in the three compounds as well as their order of magnitude. The largest value is found out in **[9]HBNG** with a value above 10^–2^ which is very remarkable for organic pure hydrocarbon dyes. On the other hand, the values for **[10]HBNG** and **[11]HBNG** are smaller by a factor close to 4 in good agreement with experiments. We have found out that the moduli of the ETDM and MTDM transition vectors are the largest in **[9]HBNG**, whereas displaying the smallest/largest angle/cosine between them; thus, the three factors add to produce the largest *g*_abs_ for **[9]HBNG** (Figures 9 and S25, see the Supporting Information, show the direction and moduli of the ETDM and MTDM for the three systems). This fact points out to the design in **[9]HBNG** to be optimal for *g*_abs_. The largest value for ETDM in **[9]HBNG** comes from a significant extension of the optical HOMO-to-LUMO one-electron excitation over the more planar HBC moiety, a situation that is more favorable in **[9]HBNG** because of the smallest helicene segment. On the other hand, the largest MTDM module, conversely, arises because there is a significant extension, among the three compounds, of the HOMO–LUMO excitation over the helicene curvature, thus promoting a pseudo-local rotation of the electron density during HOMO–LUMO excitation at the origin of the MTDM. This interpretation is supported by the electrochemical data: (i) the smallest oxidation potential of the three compounds in **[9]HBNG** indicates an important extension of the HOMO, among the three compounds, in the HBC units further helped by the inter-layer interaction. (ii) The smallest potential for the first reduction in **[9]HBNG** supports that the LUMO is greatly extended over the helicene but also with participation of the HBC unit. This synergistic combination of the frontier HOMO and LUMO orbitals between the helicene and the HBC produces the optimal situation for the increment of *g*_lum_.

As proposed by Zinna and co-workers,^45^ one may consider the brightness, defined as *B*_CPL_ = ε × Φ × *g*_lum_/2. By this way, we take into account the overall process through absorption coefficient, emission quantum yield, and dissymmetry factor efficiencies. Average values of 81, 20, and 27 for **[9]HBNG**, **[10]HBNG**, and **[11]HBNG**, respectively, are obtained (Table 6). These values are significantly higher than the average *B*_CPL_ values of helicene derivatives (*B*_CPL_ 14.10 for [9]helicenes and 1.35 for [11]helicenes)^45^ and highlight the fact that a balanced π-extension of the chromophore between the planar HBC (promoting large ETDM) and the helicene (promoting large MTDM) is an appealing alternative to obtain good emission and chiroptical properties.

**Table 6:** Experimental Data from the Circular Polarized Luminescence of the Enantioenriched HBNGs **[9]HBNG**, **[10]HBNG**, and **[11]HBNG**^c^

	ε (λ_exc_)^a^^,^^b^	Φ_Fluo_^a^	10^2^ × g_lum_	*B*_CPL_^b^
**(+)-[9]HBNG**	20,600 (440)	22	+3.60	81
**(−)-[9]HBNG**			–3.60	81
**(+)-[10]HBNG**	38,800 (420)	10	+1.10^c^	21^c^
**(−)-[10]HBNG**			–1.00	19
**(+)-[11]HBNG**	58,200 (420)	11	+0.84	27
**(−)-[11]HBNG**			–0.89	28

a ε and Φ_Fluo_ were obtained from the racemic mixture.

b M^–1^ cm^–1^.

c *g*_lum_ and *B*_CPL_ of **(+)-[10]HBNG** are corrected to optical purity (i.e., scaled by a factor of ∼1.4).

## Conclusions

In summary, we have carried out the bottom-up synthesis of two new HBNGs, namely, **[9]HBNG** and **[11]HBNG**, following a similar methodology to that used for previously synthesized **[10]HBNG**. A full comparative spectroscopic (electronic, vibrational Raman, and chiroptical), electrochemical, and solid-state study of the three compounds is carried out. Interestingly, comparison of **[9]HBNG**, **[10]HBNG**, and **[11]HBNG** helical bilayers endowed with [9], [10], and [11]helicenes embedded in their structure, respectively, afford distinctive and unexpected properties, depending upon the overlapping degree between the two HBCs, determined from the X-ray data obtained for the three NGs.

The CV reveals, unexpectedly, the strongest electron donor character for the lesser π-extended system **[9]HBNG**, which is also supported by the spectroelectrochemical studies. The significant decrease of the oxidation potential observed for **[9]HBNG** and the red shift observed in the absorption bands of the radical cation of **[9]HBNG** have been accounted for by a charge delocalization between the two HBC layers, which eventually contributes to a lower oxidation potential. Actually, this inter-layer communication could also underpin the differences found in the fluorescence spectrum measured for **[9]HBNG** when compared to compounds **[10]HBNG** and **[11]HBNG**.

The differences observed for **[9]HBNG**, due to its strong overlapping between the two layers, is also found in the exceptional chiroptical properties that it exhibits, with values of *g*_lum_ as high as 3.6 × 10^–2^.

Thus, the experimental findings show that this type of HBNG, formed by two π–π stacked HBCs covalently connected through a (chiral) helicene, resembles vdW layered 2D materials, where the overlapping between the layers defines the properties of the materials. These findings pave the way to new NGs whose properties can be controlled by the length of the embedded helicene and the inter-layer interactions.

## Supporting Information Available

Synthetic procedures, additional figures/schemes of physical properties, and characterization data (PDF)
