# Supplementary material for: Helical Bilayer Nanographenes: Impact of the Helicene Length on the Structural, Electrochemical, Photophysical, and Chiroptical Properties
Source: J Am Chem Soc. 2023 May 2;145(21):11599–610. doi: 10.1021/jacs.3c01088 (PMC10236438; doi:10.1021/jacs.3c01088)
Supplement: Supplementary file 1 — ja3c01088_si_001.pdf [file ja3c01088_si_001.pdf]

Helical Bilayer Nanographenes: Impact of the Helicene Length on the Structural, Electrochemical, Photophysical and Chiroptical Properties

Patricia Izquierdo-García,[a] Jesús M. Fernández-García,[a] Samara Medina Rivero,[b][c] Michal Šámal,[f] Jiří Rybáček,[f] Lucie Bednárová,[f]Sergio Ramírez-Barroso,[a] Francisco J. Ramírez,[b] [c] Rafael Rodríguez,[e] Josefina Perles,[d] David García-Fresnadillo,[a] Jeanne Crassous,[e] Juan Casado,[b] Irena G. Stará,[f] Nazario Martín* [a] [g]

[a] Departamento de Química Orgánica I, Facultad de Ciencias Químicas, Universidad Complutense de Madrid 28040, Spain.

[b] Departament of Physical Chemistry, Facultad de Ciencias, Universidad de Málaga, 29071 Málaga, Spain.

[c] Department of Physics & Astronomy, University of Sheffield, Sheffield S3 7RH, United Kingdom.

[d] Laboratorio DRX Monocristal, SIdI, Universidad Autónoma de Madrid, 28049 Madrid, Spain.

[e] Institut des Sciences Chimiques de Rennes (ISCR), UMR 6226 CNRS - Univ Rennes, 35000 Rennes, France

[f] Institute of Organic Chemistry and Biochemistry, Czech Academy of Sciences, Flemingovo nám. 2, 166 10 Prague 6, Czech Republic

[g] IMDEA-Nanociencia, C/ Faraday, 9, Campus de Cantoblanco, Madrid 28049, Spain.

Table of Contents

1. General 2
2. Synthetic procedures 6
3. NMR and mass spectra 11
4. Crystal structure description 21
5. Electrochemical measurements 27
6. UV-vis-NIR spectroelectrochemistry 30
7. Photophysical characterization 31
8. Vibrational Raman spectroscopy 33
9. HPLC resolution and spectral characterization 35
10. CD spectra 42
11. Quantum Chemistry Calculations 44

1. General

Unless otherwise noted, all materials including solvents were obtained from commercial suppliers and used without further purification. 97% 9,10-dichlorooctafluoroanthracene from Aldrich, 96% 4-*tert*-butylphenylacetylene, 99% CuI and 98% DDQ from Aldrich, dichloro[1,1'-bis(diphenylphosphino)ferrocene] palladium(II) complex with dichloromethane (1:1) Pd 13% and 98% TfOH from Alfa Aesar. Tetra-2,3,4,5-tetrakis[4-(1,1-dimethylethyl)phenyl]2,4-cyclopentadien-1-one was prepared according to the procedure reported in the literature.[1] The starting materials: 2,13-dichloro[5]helicene **1a**[2] and 2,17-dichloro[7]helicene **1b**[3] were synthesized according to the literature procedures. Unless otherwise noted, all reactions were performed with dry solvents (dried by filtration through alumina according to the method described)[4] and under an atmosphere of argon in dried glassware with standard vacuum-line techniques.

Microwave reactions were performed in an Anton-Parr Monowave 300 microwave reactor.

All work-up and purification procedures were carried out with reagent-grade solvents in air. Silica column chromatography was conducted with Scharlau 40-60 µm silica gel. Analytical thin-layer chromatography (TLC) was performed using E. Silica gel 60 F_{254}-coated aluminum sheets (Merck). Flash chromatography was performed on Silica gel 60 (0.040-0.063 mm, Merck). The developed chromatogram was analyzed by solution of $Ce(SO_4)_2 . 4 H_2O$ (1%) and $H_3P(Mo_3O_{10})_4$ (2%) in sulfuric acid (10%) and UV lamp (254 nm and 365 nm).

IR spectra were recorded on a FT-IR Nicolet Magna 750 spectrometer and in KBr cell in $CHCl_3$ solution on Nicolet 6700 FT-IR spectrometer (Thermo Fisher Scientific, USA) equipped with a standard mid-IR source, a KBr beam-splitter, and a DTGS detector and with a cell compartment purged by dry nitrogen.

MALDI-ToF matrices were trans-2-[3-(4-*tert*-butylphenyl)-2-methyl-2-propenylidene]-malononitrile (DCTB) or 1,8-dihidroxi-9,10-dihydroanthracen-9-ona (dithranol) and mass analysis were performed in a Bruker Ultraflex II using a LTB MNL 106 laser source.

The **APCI mass** spectra were recorded using an LTQ Orbitrap XL (Thermo Fisher Scientific) hybrid mass spectrometer equipped with an APCI ion source. The APCI vaporizer and heated capillary temperatures were set to 400 °C and 200 °C, respectively; the corona discharge current was 3.5 µA. Nitrogen served both as the sheath and

---

[1] Lungerich, D.; Hitzenberger, J. F.; Marcia, M.; Hampel, F.; Drewello, T.; Jux, N. *Angewandte Chemie International Edition* **2014**, 53 (45), 12231.

[2] Nejedlý, J.; Šámal, M.; Rybáček, J.; Gay Sánchez, I.; Houska, V.; Warzecha, T.; Vacek, J.; Sieger, L.; Buděšínský, M.; Bednárová, L.; Fiedler, P.; Císařová, I.; Starý, I.; Stará, I. G., *J. Org. Chem.* **2020**, *85*, 248.

[3] Stetsovych, O.; Mutombo, P.; Švec, M.; Šámal, M.; Nejedlý, J.; Císařová, I.; Vázquez, H.; Moro-Lagares, M.; Berger, J.; Vacek, J.; Stará, I. G.; Starý, I.; Jelínek, P., *J. Am. Chem. Soc.* **2018**, *140*, 940.

[4] Pangborn, A. B.; Giardello, M. A.; Grubbs, R. H.; Rosen, R. K.; Timmers, F. J. *Organometallics* **1996**, 15 (5), 1518.

auxiliary gas at flow rate 55 and 5 arbitrary units, respectively. The ionization conditions were the same for low-resolution as well as high-resolution experiment. The HR spectra were acquired at a resolution of 100 000.

^{1}H **NMR** spectra were recorded at 700 (Bruker AVIII), 500 (Bruker AV), 400 (Bruker AVIII) or 300 (Bruker AVIII) MHz and ^{13}C NMR spectra were recorded at 176, 126 or 101 (Bruker AV) MHz. Chemical shifts for ^{1}H NMR and ^{13}C NMR are expressed in parts per million (ppm). For referencing of NMR spectra, the residual solvent signal ($CDCl_3$ δ 7.26 ppm for ^{1}H and δ 77.16 ppm for ^{13}C) was used. Data are reported as follows: chemical shift, multiplicity (s = singlet, d = doublet, dd = doublet of doublets, t = triplet, q = quartet, m = multiplet, bs = broad singlet), coupling constant (Hz), and integration.

Electrochemical measurements were performed using a standard one-compartment, three-electrode electrochemical cell connected to an electrochemical analyzer (Metrohm Autolab). The working electrode was a glassy carbon electrode (3 mm diameter) that was freshly polished with a suspension of Al_2O_3 in distilled water and sonically rinsed with acetone before each measurement. Silver (Ag/0.1 M $AgNO_3$ in CH_3CN) and platinum wires were used as reference and counter electrodes, respectively. Electrochemical grade (Aldrich) tetrabutylammonium hexaflurophosphate 0.1 M in toluene:acetonitrile (4:1) was used as supporting electrolyte. All measurements were conducted under dry argon. Solutions were saturated with argon for deoxygenation and to maintain an argon blanket for at least 10 minutes prior to each measurement. All measurements are referenced to Fc/Fc^{+} added as internal reference.

Spectroelectrochemical Measurements. *In situ* UV-vis-NIR spectroelectrochemical oxidations were conducted on a Varian Cary 5000 UV-vis-NIR spectrophotometer. A C3 epsilon potentiostat from BASi was used for the electrolysis using a thin layer cell from a demountable omni cell from Specac. In this cell a three electrodes system was coupled to conduct in situ spectroelectrochemistry. A Pt gauze was used as the working electrode, a Pt wire was used as the counter electrode, and an Ag wire was used as the pseudo-reference electrode. The spectra were collected at constant electrolysis potential and the potentials were changed in interval of 10 mV. The electrochemical medium used was 0.1 M tetrabutylammonium hexafluorophosphate, Bu_4NPF_6, in fresh distilled dichloromethane, at room temperature with sample concentrations of 10^{-3} M.

Photophysical characterization was performed as follows. UV-vis absorption spectra were recorded on a UV-Vis spectrophotometer (Varian Cary 50) using 1 cm quartz cuvette (Suprasil). Room-temperature fluorescence spectra were acquired on a spectrofluorimeter (Horiba FluoroLog 3) equipped with a high-pressure Xenon lamp and a Hamamatsu R928P photomultiplier tube. Fluorescence spectra were corrected for the characteristics of the lamp source and of the detection system. Fluorescence lifetimes (τ) were measuring via time-correlated single photon counting (TCSPC) measurements employing an Acton SP2500 spectrometer equipped with a PMA 06 photomultiplier (PicoQuant) and a HydraHarp-400 TCSPC event timer with 1 ps time resolution. The excitation source was a 405 nm picosecond pulsed diode laser (LDH-D-C-405, PicoQuant)

driven by a PDL828 driver (PicoQuant) with FWHM < 70 ps. Fluorescence decays were analyzed using PicoQuant Fluofit v4.6.5 data analysis software.

Vibrational Raman Spectroscopy. The 1064 nm Raman spectra of ***t*Bu-HBC**, **[10]HBNG** and **[11]HBNG** in solid state at room temperature were measured using the RAMII FT-Raman module of a VERTEX 70 FT-IR spectrometer (Bruker). A continuous–wave Nd–YAG laser working at 1064 nm was employed for excitation, at a laser power in the sample not exceeding 10 mW. Raman scattering radiation was collected in a back–scattering configuration with a standard spectral resolution of 4 cm^{-1}. 10000 scans were averaged for each spectrum.

The 325 nm Raman spectrum of **[9]HBNG** in solid state at room temperature was obtained with a JASCO NRS-5000 dispersive laser Raman spectrometer with a confocal microscope and equipped with a Peltier cooled CCD detector operating at –60 ^{o}C. Raman spectrum was recorded by averaging spectra during 16 min with a resolution of 1-2 cm^{-1}.

Optical rotations were measured using an Autopol IV instrument (Rudolph Research Analytical).

HPLC analyses were performed on Knauer Smartline 1000/2500 isocratic system with polarimetric detector (Chiralyser MP, IBZ Messtechnik) connected to Clarity chromatography station. Semipreparative HPLC resolutions were performed on PuriFlash 5.250 (Interchim) quaternary gradient HPFC/HPLC system equipped with DAD detector.

The **ECD** spectra were measured on a Jasco 1500 spectropolarimeter (JASCO International Co. Ltd.) equipped with a fluorescence emission monochromator (FMO522) and separate fluorescence emission detector (FDT-538). The ECD spectra were measured over a spectral range of 210 nm to 550 nm in tetrahydrofuran (10^{-4} M solutions). Measurements were made in a quartz cell with a 0.1 cm path length using a scanning speed of 20 nm/min, a response time of 4 seconds and standard instrument sensitivity. After a baseline correction, the CD spectra were expressed in terms of differential molar extinction (Δε).

The **circularly polarized luminescence** (**CPL**) measurements were performed using a home-built CPL spectrofluoropolarimeter (constructed with the help of the JASCO Company). The samples were excited using a 90° geometry with a Xenon ozone-free lamp 150 W LS. The following parameters were used: emission slit width ≈ 10 mm, integration time = 8 sec, scan speed = 50 nm/min, accumulations = 5. The concentration of all the samples was ca. 10^{-6} M.

Quantum Chemistry Calculations. Density Functional Theory (DFT) quantum chemical calculations were performed with the Gaussian'16 suite of programs.[5] The Becke's three parameter (B3)[6] gradient-corrected exchange functional was used, and the non-local

---

[5] M. J. Frisch et al. Gaussian 16, Revision A.03, Gausian, INc. Wallingford CT, **2016**.

[6] Becke, A.D. *J. Chem. Phys.* **1993**, *98*, 5648.

correlation was provided by the Lee-Yang-Parr (LYP) expressions.[7-8] To achieve an improved description of long-range interactions we used the Coulomb-attenuating hybrid method (CAM-B3LYP),[9] which includes the Hartree-Fock and the Becke exchanges as a variable ratio depending of the intermolecular distance. Structural optimizations and spectroscopic features were calculated using the split-valence 3-21g(d) basis set.[10-11] Raman spectra were obtained from the calculated DFT intensities, and the vibrational wavenumbers uniformly scaled by 0.96.[12] Every band was represented by a Gaussian function of 10 cm^{-1} half-height width. Electronic excitation energies were obtained by using the time-dependent DFT (TDDFT) formalism[13][14] for which up to the fifty low-lying energy states were considered.

---

[7] Stephens, P.J.; Devlin, F.J.; Chabalowski, C.F.; Frisch, M.J. *J. Phys. Chem.* **1994,** *98,* 11623.
[8] Kim, K.; Jordan, K.D. *J. Phys. Chem.* **1994,** *98,* 10089.
[9] T. Yanai, D.P. Tew, N. C. Nicholas, *Chem. Phys. Lett.* **2004**, *393*, 51.
[10] Hariharan, P.C.; Pople, J.A. *Theor. Chim. Acta* **1973**, *28*, 213.
[11] Clark, T.; Chandrasekhar, J.; Spitznagel, G.W.; Schleyer, P.V.R. *J. Comput. Chem*. **1983**, *4*, 294.
[12] A.P. Scott, L. Radom *J. Phys. Chem.* **1996**, *100*, 16502.
[13] E. Runge, E.K.U. Gross *Phys. Rev. Lett.* **1984**, *52*, 997.
[14] E.K.U. Gross, W. Kohn *Adv. Quant. Chem.* **1990**, *21*, 255.

2. Synthetic procedures

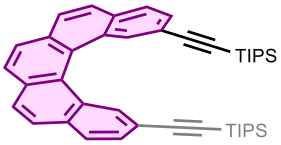

(Pentahelicene-2,13-diyldiethyne-2,1-diyl)bis[tris(1-methylethyl)silane] 2a. A pressure tube was charged with dichlorohelicene **1a**[2] (300 mg, 0.865 mmol, 1.0 equiv.), $Pd_2(dba)_3$ (16 mg, 0.017 mmol, 2 mol%), XPhos (33 mg, 0.0688 mmol, 8 mol%), potassium carbonate (713 mg, 5.160 mmol, 6.0 equiv.), flushed with nitrogen, and degassed (by three freeze-pump-thaw cycles) DMF (15 mL) was added. Then (triisopropylsilyl)acetylene (770 µL, 3.44 mmol, 4.0 equiv.) was added and the reaction mixture was stirred at 120 °C for 16 h. The solvent was evaporated under reduced pressure and the residue was purified by flash chromatography on silica gel (hexane to hexane-ethyl acetate 20:1) to afford diyne **2a** (418 mg, 76%) as an amorphous solid.

^{1}H NMR (400 MHz, $CDCl_3$) δ= 8.64 (bd, *J* = 1.3 Hz, 2H), 7.83 – 7.90 (m, 8H), 7.53 (dd, *J* = 8.3, 1.5 Hz, 2H), 1.02 – 1.09 (m, 42H).
^{13}C NMR (101 MHz, $CDCl_3$) δ= 133.2, 132.6, 132.1, 130.4, 128.9, 127.7, 127.5, 127.4, 127.1, 126.6, 120.0, 107.7, 90.4, 18.9, 18.8, 11.5.
IR ($CHCl_3$): 3064 w, 3049 w, 2959 s, 2944 s, 2923 m, sh, 2891 m, 2865 vs, 2152 m, 1606 w, 1502 w, 1463 m, 1438 w, 1383 w, 1367 w, 1301 w, 1189 w, 1072 w, 996 m, 883 m, 849 vs, 678 m, 660 m, 634 w cm^{-1}.
APCI MS: 639 ([M+H]$^+$).
HR APCI MS: calcd for $C_{44}H_{55}Si_2$ 639.3842, found 639.3835.

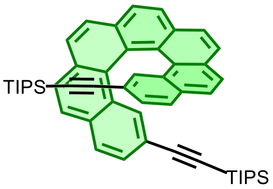

(Heptahelicene-2,17-diyldiethyne-2,1-diyl)bis[tris(1-methylethyl)silane] 2b. A pressure tube was charged with helicene **1b**[3] (450 mg, 1.011 mmol, 1.0 equiv.), $Pd_2(dba)_3$ (18 mg, 0.0202 mmol, 2 mol%), XPhos (38 mg, 0.0807 mmol, 8 mol%), potassium carbonate (837 mg, 6.060 mmol, 6.0 equiv.), flushed with nitrogen, and degassed (by three freeze-pump-thaw cycles) DMF (20 mL) was added. Then (triisopropylsilyl)acetylene (910 µL, 4.041 mmol, 4.0 equiv.) was added and the reaction mixture was stirred at 120 °C for 16 h. The solvent was evaporated under reduced pressure and the residue was purified by flash chromatography on silica gel (hexane to hexane-ethyl acetate 20:1) to afford diyne **2b** (530 mg, 71%) as an amorphous solid.
^{1}H NMR (400 MHz, $CDCl_3$) δ= 8.04 (s, 2H), 8.00 (d, *J* = 8.2 Hz, 2H), 7.87 (d, *J* = 8.2 Hz, 2H), 7.73 (d, *J* = 8.5 Hz, 2H), 7.50 (d, *J* = 8.5 Hz, 2H), 7.30 – 7.33 (m, 2H), 7.27 (d, *J* = 8.3 Hz, 2H), 6.99 (dd, *J* = 8.2, 1.6 Hz, 2H), 1.09 – 1.16 (m, 42H).
^{13}C NMR (101 MHz, $CDCl_3$) δ= 132.2, 131.53, 131.46, 129.1, 128.8, 128.1, 127.8, 127.7, 127.4, 127.1, 127.0, 126.8, 126.7, 125.0, 118.9, 107.6, 89.0, 18.9, 11.5.
IR ($CHCl_3$): 3055 w, 2959 m, 2944 m, 2925 m, sh, 2892 w, 2865 m, 2152 w, 1606 w, 1518 w, 1495 w, 1463 w, 1428 w, 1390 w, sh, 1383 w, 1366 w, 1308 w, 1191 w, 1073 w, 996 w, 883 w, 847 vs, 679 w, 660 w, 641 w cm^{-1}.
APCI MS: 739 ([M+H]$^+$).
HR ESI MS: calcd for $C_{52}H_{59}Si_2$ 739.4155, found 739.4148.

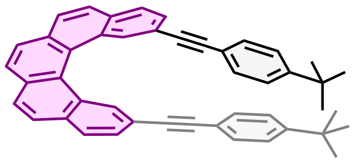

3a. A 25 mL dry round bottom flask provided with a magnetic stir bar was charged with helicene **2a** (100 mg, 0.156 mmol, 1 equiv.), $Pd(PPh_3)_2Cl_2$ (12 mg, 0.018 mmol, 0.1 equiv.) and CuI (125 mg, 0.656 mmol, 4 equiv.). The flask was evacuated and backfilled with dry Ag three times to exclude moisture and air. Dry THF (1 mL), freshly distilled DIPA (1.5 mL) and 4-*tert*-butyliodobenzene (86 mg, 0.329 mmol, 2.0 equiv) were added to the flask and stirred for 5 minutes before adding tetrabutylammoniumfloride (TBAF 1.0 M in THF, 0.5 mL, 0.5 mmol, 3.0 equiv) in one portion. The mixture was stirred at 30 °C for 16 hours. Silica was poured into the flask and solvent was removed under reduced pressure to adsorb the mixture which was then purified by silica gel column chromatography with hexane:DCM 10:1 affording the product as a white solid (83 mg, 86%).

^{1}H NMR (300 MHz, $CDCl_3$) δ= 8.79 (s, 2H), 7.95 – 7.85 (m, 8H), 7.65 (dd, *J* = 8.3, 1.4 Hz, 2H), 7.37 (d, *J* = 8.0 Hz, 4H), 7.30 (d, *J* = 8.5 Hz, 4H), 1.30 (d, *J* = 0.9 Hz, 18H).

^{13}C NMR (75 MHz, $CDCl_3$) δ= 151.4, 132.8, 132.2, 132.1, 131.5, 130.4, 129.2, 128.1, 127.7, 127.5, 127.2, 126.7, 125.3, 120.6, 120.0, 89.7, 89.4, 34.9, 31.3.

IR (CH_2Cl_2): 3044, 2958, 2863, 2149, 1907, 1793, 1698, 1607, 1517, 1463, 1363, 1266, 846, 727, 674, 561.

HR-MS MALDI-ToF: calcd for $C_{46}H_{38}$ 590.2974, found 590.2994.

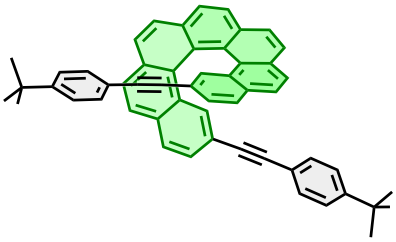

3b. A 25 mL dry Schlenk provided with a magnetic stir bar was charged with helicene **2b** (100 mg, 0.135 mmol, 1 equiv.), $Pd(PPh_3)_2Cl_2$ (10 mg, 0.013 mmol, 0.1 equiv.) and CuI (100 mg, 0.541 mmol, 4 equiv.). The Schlenk was evacuated and backfilled with dry Ag two times to exclude moisture and air. Dry THF (5 mL), freshly distilled DIPA (1.4 mL) and 4-*tert*-butyliodobenzene (140 mg, 0.541 mmol, 4.0 equiv) were added to the Schlenk and stirred for 5 minutes before adding tetrabutylammoniumfloride (TBAF 1.0 M in THF, 0.41 mL, 0.41 mmol, 3.0 equiv) in one portion. The mixture was stirred at 30 °C for 20 hours. The crude was poured into a flask and silica was added, solvent was removed under reduced pressure to adsorb the mixture which was then purified by silica gel column chromatography with hexane:DCM 100:20 affording the product **3b** as a white solid (63 mg, 67%).

^{1}H NMR (300 MHz, $CDCl_3$) δ= 8.07 (s, 2H), 8.07 (d, *J* = 8.2 Hz, 2H), 8.00 (d, *J* = 8.2 Hz, 2H), 7.79 (d, *J* = 8.5 Hz, 2H), 7.54 (d, *J* = 8.5 Hz, 2H), 7.44 – 7.38 (m, 10H), 7.32 (d, *J* = 8.2 Hz, 2H), 7.04 (dd, *J* = 8.2, 1.6 Hz, 2H), 1.37 (s, 18H).

^{13}C NMR (126 MHz, $CDCl_3$) δ= 151.3, 132.2, 131.7, 131.5, 131.3, 129.1, 128.8, 127.8, 127.6, 127.5, 127.4, 127.1, 127.04, 127.01, 126.9, 125.5, 125.1, 120.9, 118.9, 89.7, 88.8, 35.0, 31.4.

IR (CH_2Cl_2): 3048, 2959, 2923, 2858, 1907, 1732, 1605, 1503, 1462, 1363, 1264, 1102, 1017, 834, 549.

HR-MS MALDI-ToF: calcd for $C_{54}H_{42}$ 690.3287, found 690.3267.

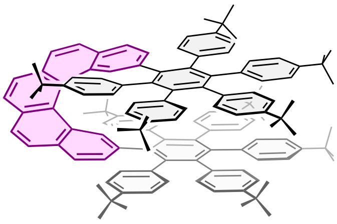

4a To a 30 mL microwave vial provided with a magnetic stir bar was added **3a** (40 mg, 0.068 mmol, 1.0 equiv) and tetra-2,3,4,5-tetrakis[4-(1,1-dimethylethyl)phenyl]2,4-cyclopen tadien-1-one (128 mg, 0.210 mmol, 3.1 equiv). The vial was placed in the microwave reactor and heated to 280 °C with a hold time of 40 minutes and stirring at 300 rpm, same conditions were repeated for 6 times. After cooling, methanol was added and the crude was sonicated to obtained a suspension. The solid was filtered to reach to compound **4a** as a nude solid (33 mg, 28%).

^{1}H NMR (300 MHz, $CDCl_3$) δ= 8.42 (s, 2H), 7.55 (d, *J* = 8.5 Hz, 2H), 7.46 (d, *J* = 8.5 Hz, 2H), 7.34 (s, 2H), 7.23 (s, 2H), 7.05 – 6.57 (m, 32H), 6.51 (d, *J* = 8.7 Hz, 2H), 6.11 (dd, *J* = 8.1, 2.0 Hz, 2H), 5.99 (d, *J* = 9.1 Hz, 2H), 5.92 (d, *J* = 8.3 Hz, 2H), 5.84 (d, *J* = 8.3 Hz, 2H), 1.24 (s, 18H), 1.14 (s, 18H), 1.09 (s, 18H), 1.01 (s, 18H), 0.57 (s, 18H).

^{13}C NMR (126 MHz, $CDCl_3$) δ= 148.1, 147.3, 147.2, 147.1, 146.7, 141.3, 141.0, 140.9, 140.2, 139.7, 138.5, 138.4, 138.2, 138.0, 137.9, 137.9, 135.7, 133.1, 132.0, 131.6, 131.2, 130.7, 130.4, 130.1, 129.4, 128.9, 127.2, 126.1, 126.0, 125.7, 124.9, 123.8, 123.0, 122.8, 122.2, 122.1, 34.4, 34.0, 34.0, 33.9, 33.3, 31.5, 31.3, 31.2, 31.1, 30.8.

IR (CH_2Cl_2): 3088, 3053, 2962, 2905, 2870, 1910, 1746, 1615, 1514, 1462, 1393, 1361, 1270, 1120, 1020, 832, 575.

HR-MS MALDI-ToF: calcd for $C_{134}H_{142}$ 1751.1111, found 1751.1151.

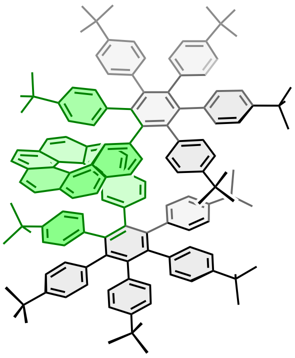

4b. To a 10 mL microwave vial provided with a magnetic stir bar was added **3b** (30 mg, 0.043 mmol, 1.0 equiv) and tetra-2,3,4,5-tetrakis[4-(1,1-dimethylethyl)phenyl]2,4-cyclopenta dien-1-one (87 mg, 0.14 mmol, 3.1 equiv). The vial was placed in the microwave reactor and heated to 280 °C with a hold time of 45 minutes and stirring at 600 rpm. After cooling, methanol was added and the crude was sonicated to obtained a suspension. The solid was filtered to reach to compound **4b** as a nude solid (36 mg, 45%).

^{1}H NMR (500 MHz, $CDCl_3$) δ= 7.89 (s, 2H), 7.81 (d, *J* = 8.2 Hz, 2H), 7.72 (d, *J* = 8.2 Hz, 2H), 7.59 z(d, *J* = 8.6 Hz, 2H), 7.55 (d, *J* = 8.6 Hz, 2H), 7.13 (d, *J* = 8.2 Hz, 2H), 6.88 – 6.50 (m, 34H), 6.47 (d, J = 8.1 Hz, 2H), 5.96 (d, J = 8.1 Hz, 2H), 5.85 (d, J = 8.0 Hz, 2H), 5.65 (s, 4H), 1.14 (s, 18H), 1.12 (s, 18H), 1.09 (s, 18H), 1.06 (s, 18H), 0.43 (s, 18H).

^{13}C NMR (126 MHz, $CDCl_3$) δ= 147.5, 147.4, 147.3, 147.2, 146.2, 140.6, 140.3, 140.3, 139.1, 138.7, 138.4, 138.2, 137.6, 137.2, 136.9, 132.6, 132.2, 132.0, 131.7, 131.3, 131.2, 131.1, 131.0, 130.4, 129.5, 129.2, 129.0, 128.4, 127.9, 127.4, 126.2, 125.7, 125.4, 124.7, 124.6, 123.2, 123.1, 123.0, 122.9, 122.3, 122.1, 34.2, 34.2, 34.1, 34.1, 33.3, 31.6, 31.4, 31.4, 31.3, 30.7.

IR (CH_2Cl_2): 3051, 2961, 2904, 2866, 1899, 1695, 1610, 1510, 1461, 1361, 1270, 831, 572.

HR-MS MALDI-ToF: calcd for $C_{142}H_{146}$ 1851.1424, found 1851.1429.

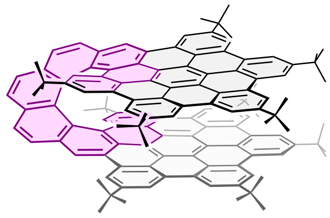

[9]HBNG. To a 100 mL flask with magnetic stir bar was added polyphenylene **4a** (20 mg, 0.011 mmol, 1 equiv.). The flask was purged and backfilled with argon and dry diclorometHane (50 mL) was added. A glass Pasteur pipette was attached to an argon inlet and dipped into the solution. Argon was bubbled continuously through the solution, while stirring, for the duration of the reaction. The mixture was cooled to 0°C in an ice water bath and 2,3-dichloro-5,6-dicyano-1,4-benzoquinone (DDQ) (360 mg, 1.586 mmol, 140 equiv.) was added in one portion. The mixture was stirred for 5 minutes and trifluoromethanesulfonic acid (1.20 mL, 2.052 g, 13.67 mmol, 1200 equiv) was added dropwise. The mixture was stirred at 0 °C for 20 minutes. After that time a $HNaCO_3$ saturated solution was added to the reaction flask and the crude was extracted with DCM and washed twice with a $HNaCO_3$ saturated solution (50 mL) and twice with brine (50 mL). From this step the organic phases were dried with $MgSO_4$ and filtered, the solvent was removed under reduced pressure. The mixture was purified by silica gel column in hexane:DCM 10:1, affording compound **[9]HBNG** as a yellow solid (12 mg, 61%).

^{1}H NMR (500 MHz, $CDCl_3$) δ= 9.18 (s, 2H), 9.13 (s, 2H), 9.06 (s, 2H), 8.87 (s, 2H), 8.75 (s, 2H), 8.71 (s, 2H), 8.39 (s, 2H), 8.21 (s, 2H), 8.20 (s, 2H), 8.14 (s, 2H), 8.13 (s, 2H), 7.96 (d, *J* = 8.1 Hz, 2H), 7.86 (d, *J* = 8.1 Hz, 2H), 7.61 (s, 2H), 1.93 (s, 18H), 1.86 (s, 18H), 1.82 (s, 18H), 1.43 (s, 18H), 1.21 (s, 18H).

^{13}C NMR (126 MHz, $CDCl_3$) δ= 147.9, 147.7, 147.4, 146.8, 146.5, 130.4, 130.3, 130.3, 130.2, 129.9, 129.4, 129.3, 129.0, 128.9, 128.8, 128.7, 127.9, 127.9, 127.4, 126.8, 126.6, 125.7, 123.7, 123.6, 123.5, 123.4, 122.9, 122.6, 122.3, 119.8, 119.8, 119.6, 119.5, 119.3, 119.2, 119.0, 118.9, 118.7, 118.3, 118.0, 117.3, 117.2, 117.2, 116.7, 35.9, 35.8, 35.6, 35.2, 34.6, 32.6, 32.3, 32.3, 32.0, 31.6.

IR (CH_2Cl_2): 3102, 3046, 2953, 2906, 2868, 1745, 1606, 1575, 1478, 1361, 1261, 1201, 867.

HR-MS MALDI-ToF: calcd for $C_{134}H_{118}$ 1726.9233, found 1726.9267.

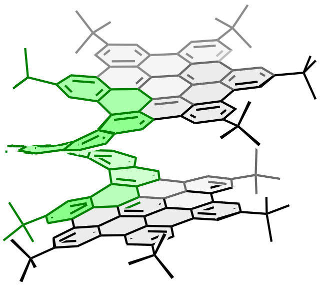

[11]HBNG. To a 250 mL flask with magnetic stir bar was added polyphenylene **4b** (55 mg, 0.094 mmol, 1 equiv.). The flask was purged and backfilled with argon and dry diclorometHane (90 mL) was added. A glass Pasteur pipette was attached to an argon inlet and dipped into the solution. Argon was bubbled continuously through the solution, while stirring, for the duration of the reaction. The mixture was cooled to 0 °C in an ice water bath and 2,3-dichloro-5,6-dicyano-1,4-benzoquinone (DDQ) (320 mg, 1.409 mmol, 44 equiv) was added in one portion. The mixture was stirred for 5 minutes and trifluoromethanesulfonic acid (1.16 mL, 1.984 g, 13.22 mmol, 141 equiv) was added dropwise. The mixture was stirred at 0 °C for 1 hour, after that time a $HNaCO_3$ saturated solution was added to the reaction flask and the crude was extracted with DCM and washed twice with a $HNaCO_3$ saturated solution (90 mL) and twice with brine (90 mL). From this step the organic phases were dried with $MgSO_4$ and filtered, the solvent was removed under reduced pressure. The mixture was purified by silica gel column in hexane:DCM 80:20, affording compound **[11]HBNG** as a yellow solid (42 mg, 75%).

^{1}H NMR (700 MHz, $CDCl_3$) δ = 9.47 (s, 4H), 9.46 (d, *J* = 7.4 Hz, 4H), 9.38 (d, *J* = 7.4 Hz, 4H), 9.12 (s, 2H), 8.95 (s, 2H), 8.44 (s, 2H), 8.20 (s, 2H), 7.35 (s, 2H), 6.93 (d, *J* = 1.6 Hz, 2H), 6.90 (d, *J* = 7.5 Hz, 2H), 6.11 – 6.08 (m, 4H), 5.32 (d, *J* = 7.9 Hz, 2H), 1.96 (s, 18H), 1.95 (s, 18H), 1.94 (s, 18H), 1.87 (s, 18H), 0.89 (s, 18H).
^{13}C NMR (176 MHz, $CDCl_3$) δ = 149.3, 149.2, 149.1, 146.4, 131.6, 131.1, 131.0, 130.9, 130.8, 130.7, 130.7, 130.6, 130.4, 130.4, 129.1, 128.1, 127.5, 126.9, 126.2, 125.9, 125.2, 125.2, 124.9, 124.9, 124.6, 124.5, 124.2, 123.9, 123.7, 123.0, 121.3, 121.1, 120.6, 120.5, 120.5, 120.2, 119.9, 119.9, 119.8, 119.2, 119.1, 119.0, 119.0, 118.7, 118.5, 118.3, 118.0, 36.0, 36.0, 36.0, 34.4, 32.6, 32.3, 31.3.
IR (CH_2Cl_2): 3077, 3045, 2955, 2906, 2870, 1605, 1572, 1479, 1369, 1257, 870.
HR-MS MALDI-ToF: calcd for $C_{142}H_{122}$ 1826.9547, found 1826.9566.

3. NMR and mass spectra

SAM, $CDCl_3$, 400 MHz, 32 scans, 300 K, 22/02/2022

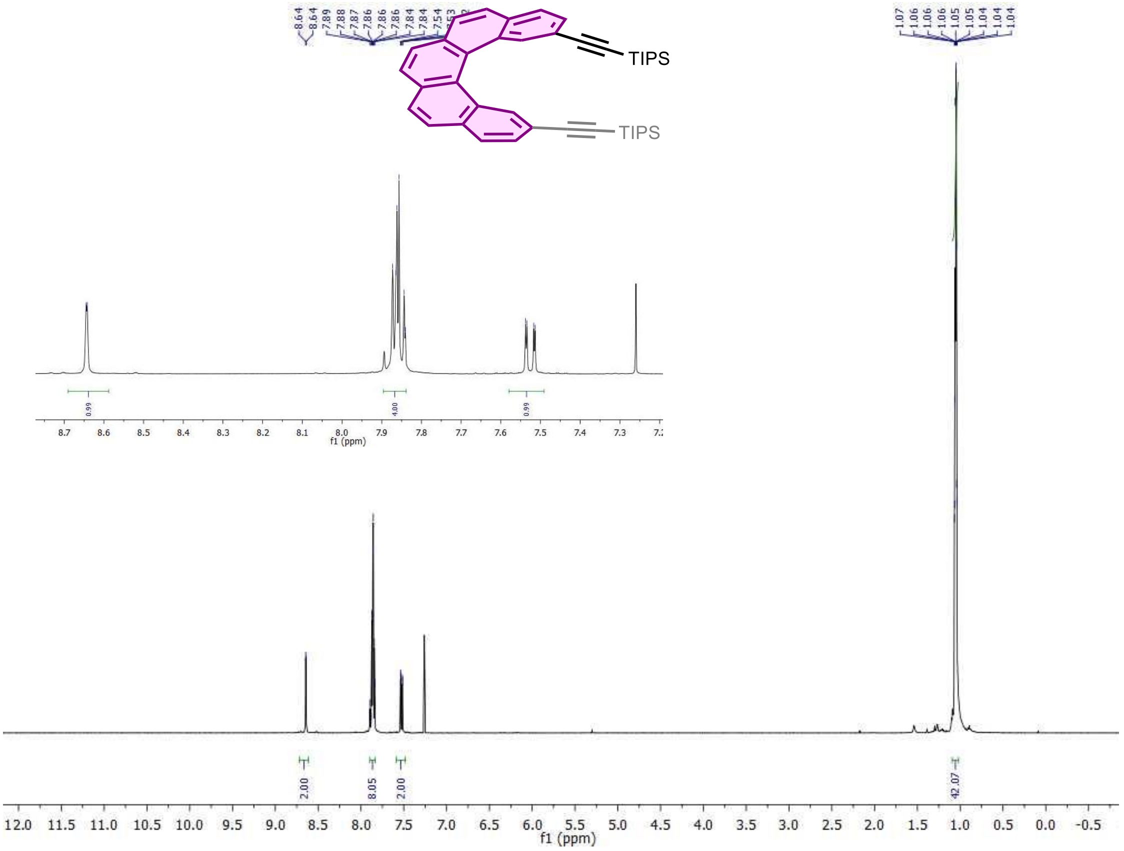

SAM, $CDCl_3$, 100 MHz, 1536 scans, 300 K, 24/02/2022

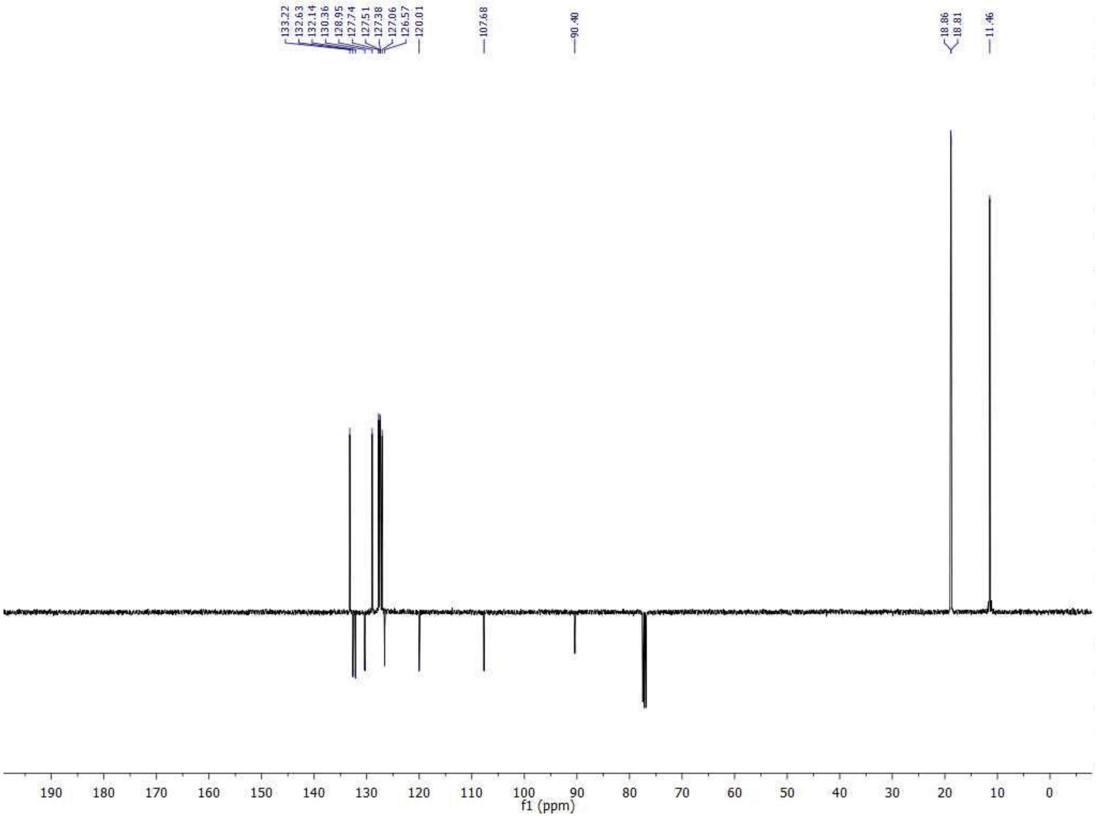

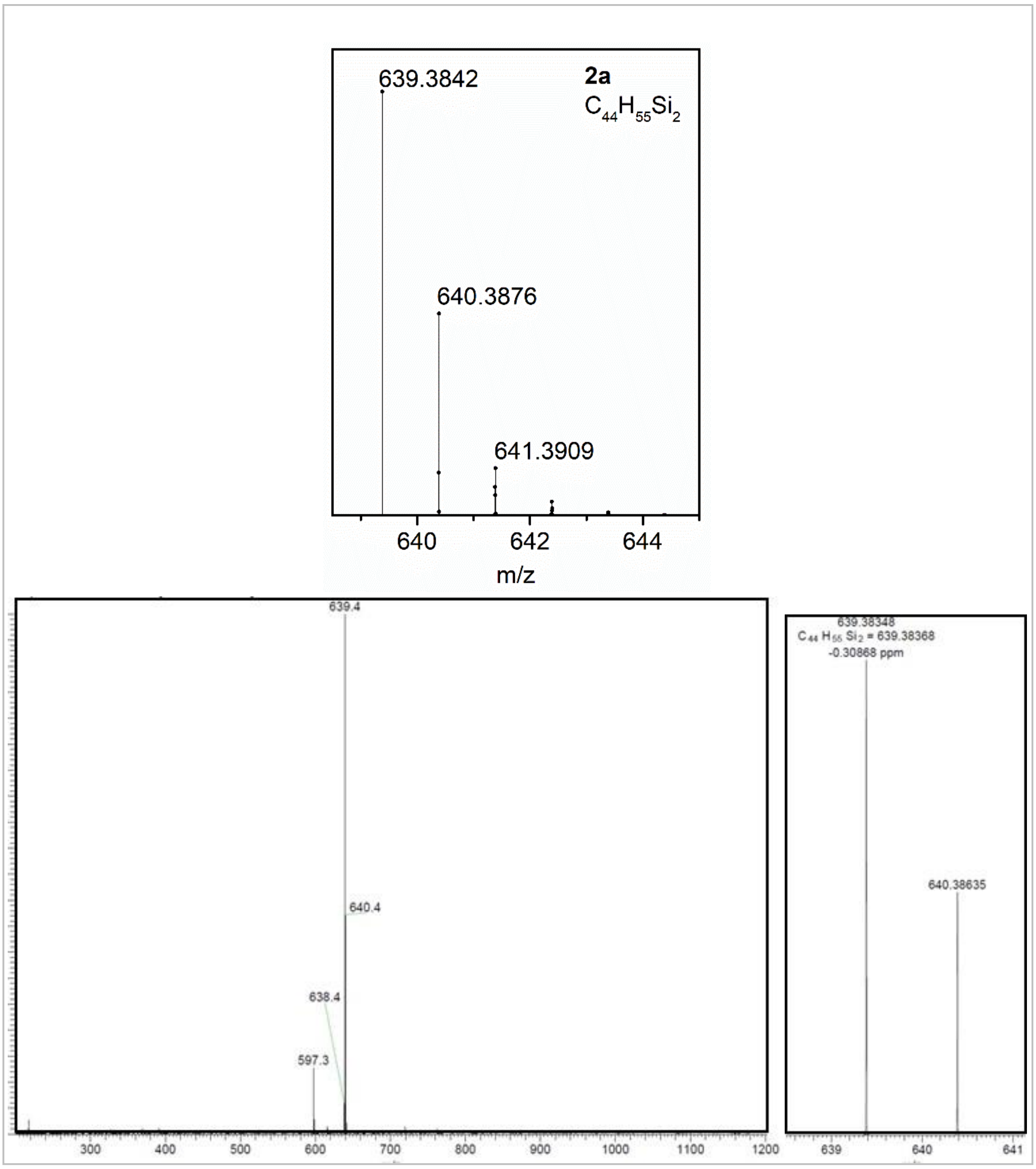

Figure S1. Compound ***rac*-2a** ^{1}H, ^{13}C-DEPT and mass spectra: theoretical (top), HR ESI MS (bottom, left) and peak zoom (bottom, right).

SAM, $CDCl_3$, 400 MHz, 32 scans, 300 K, 22/02/2022

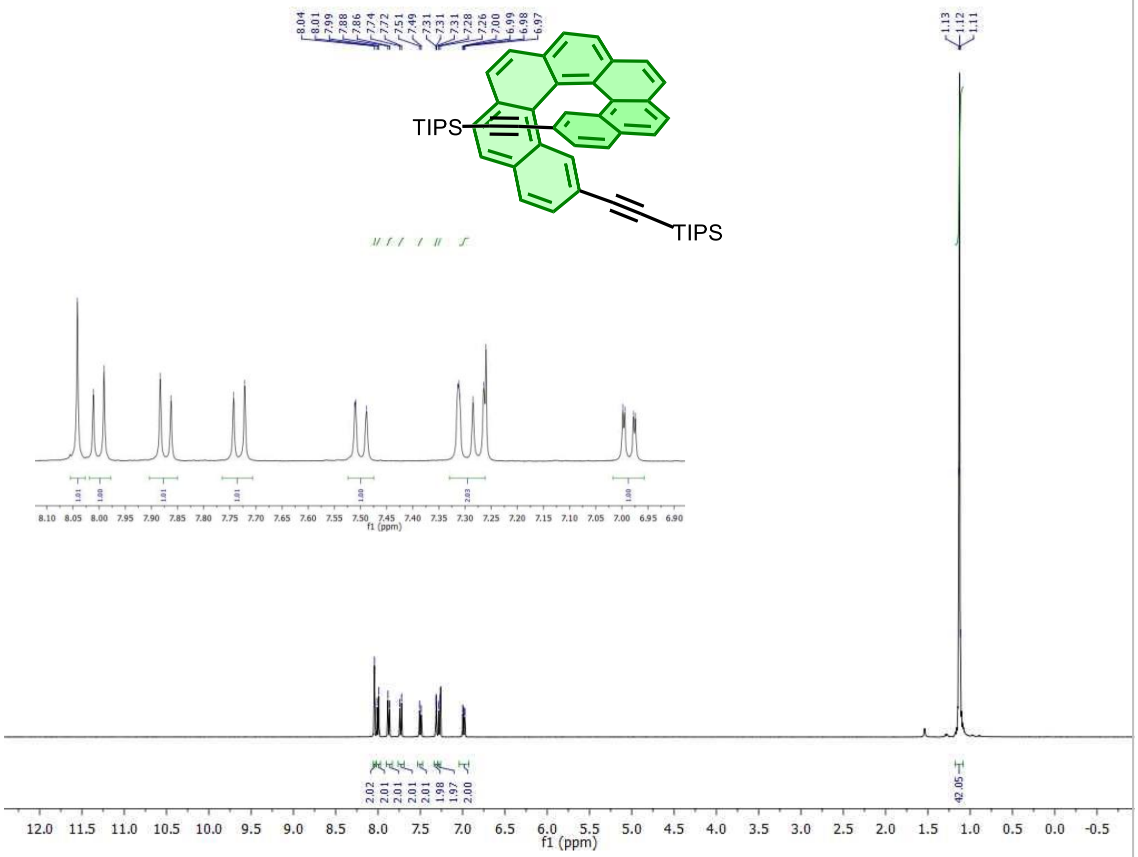

SAM, $CDCl_3$, 100 MHz, 1536 scans, 300 K, 24/02/2022

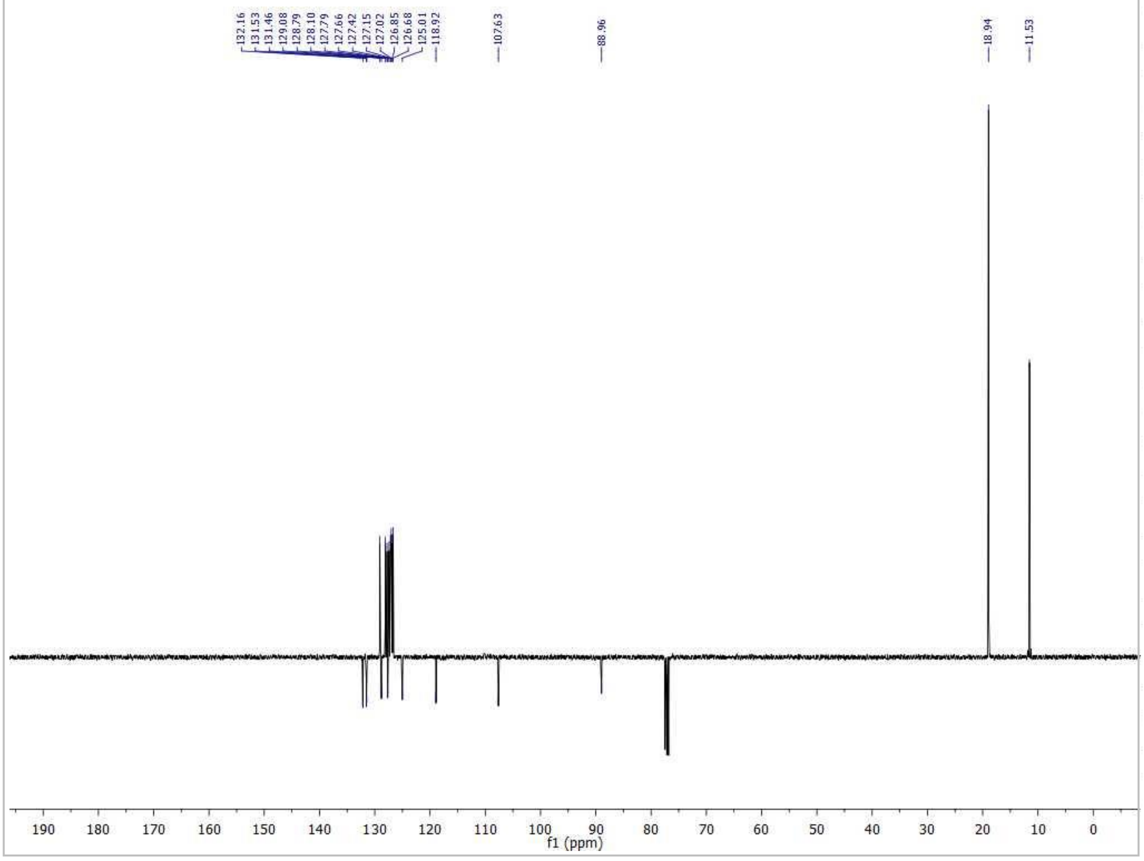

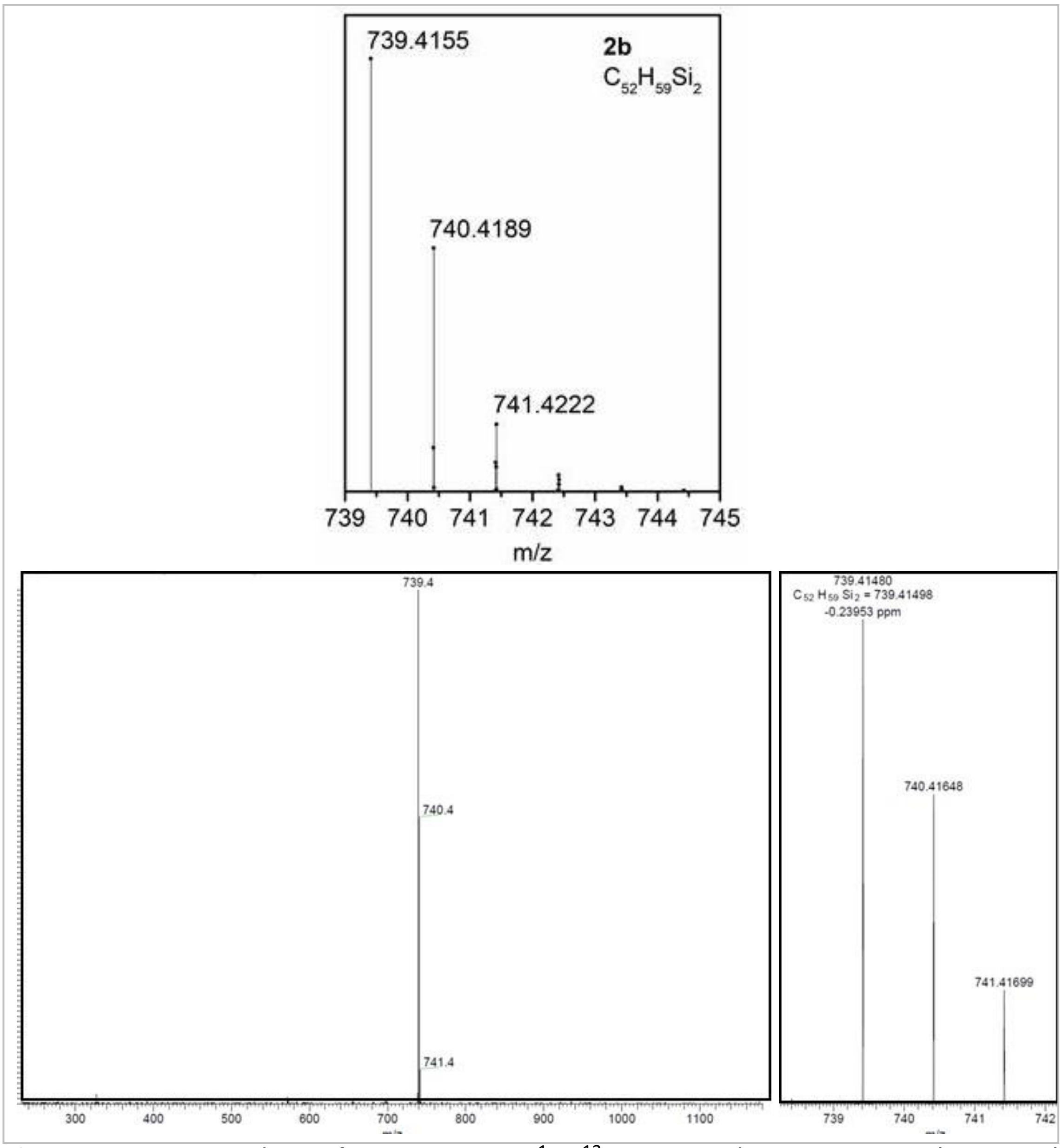

Figure S2. Compound ***rac*-2b** NMR spectra: ^{1}H, ^{13}C-DEPT and mass spectra: theoretical (top), HR ESI MS (bottom, left) and peak zoom (bottom, right).

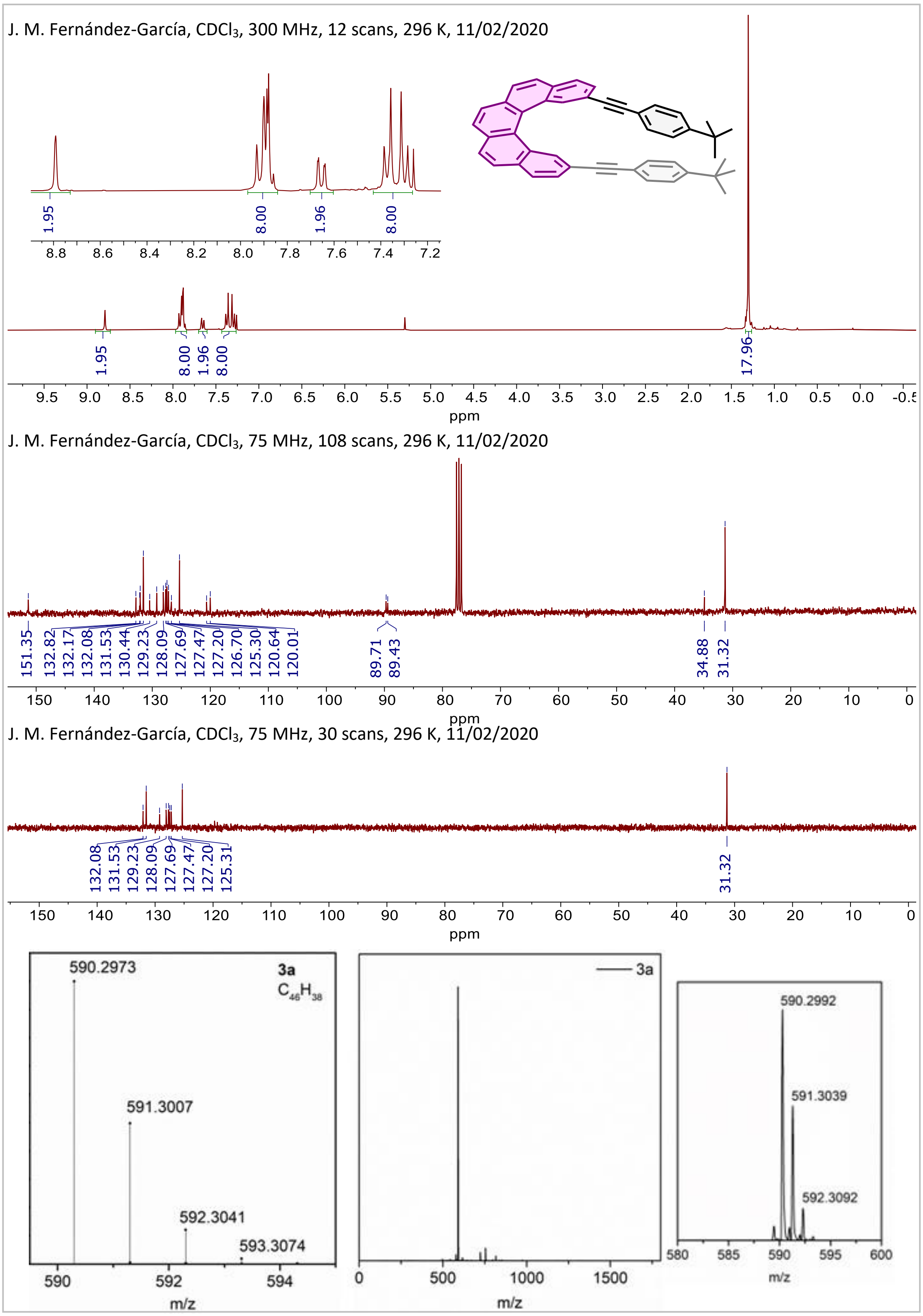

Figure S3. Compound ***rac*-3a** NMR spectra: ^{1}H, ^{13}C and DEPT and mass spectra: theoretical (left), MALDI-ToF (middle)and peak zoom (right).

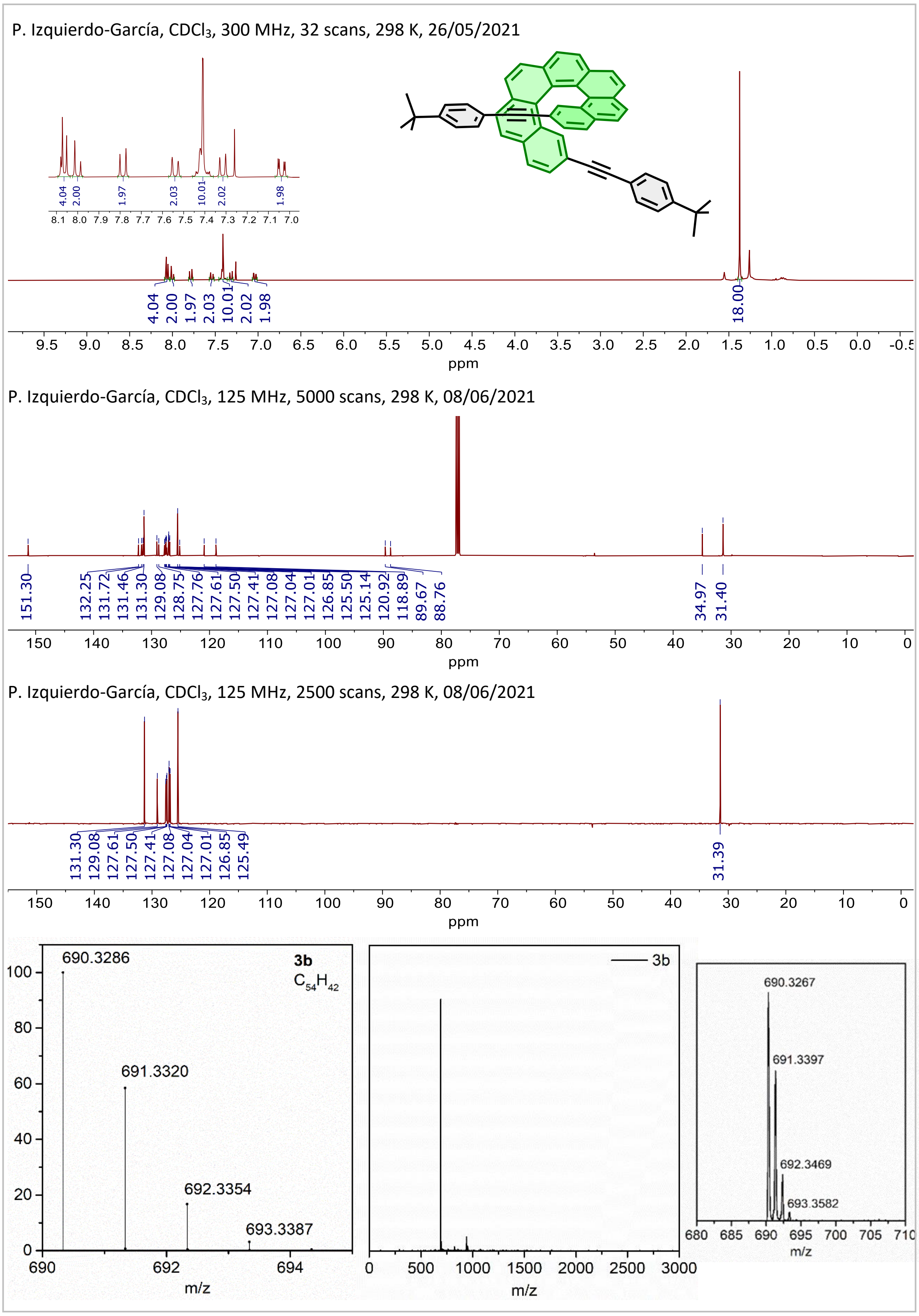

Figure S4. Compound ***rac*-3b** NMR spectra: ^{1}H, ^{13}C and DEPT and mass spectra: theoretical (left), MALDI-ToF (middle)and peak zoom (right).

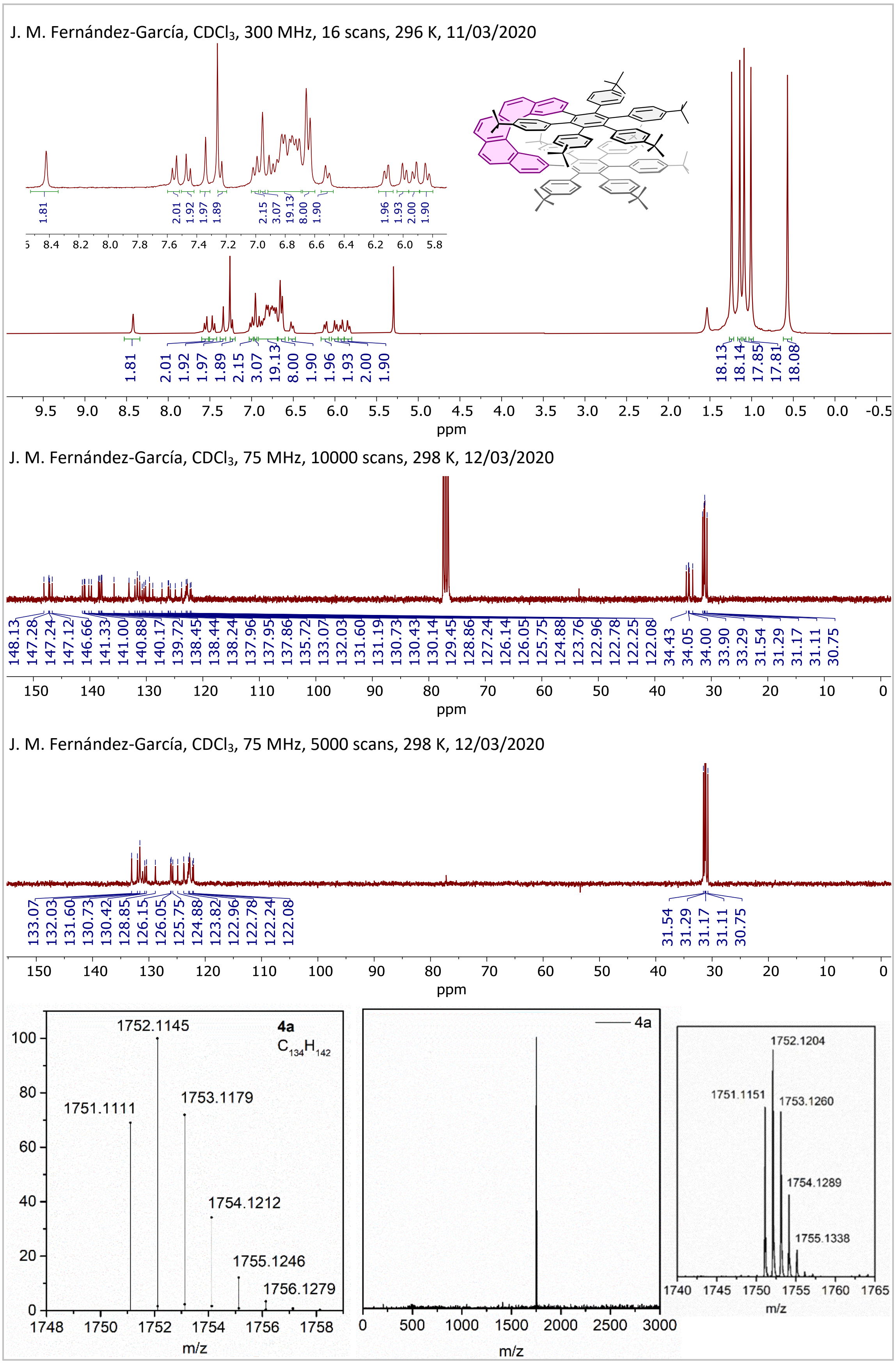

Figure S5. Compound ***rac*-4a** NMR spectra: ^{1}H, ^{13}C and DEPT and mass spectra: theoretical (left), MALDI-ToF (middle) and peak zoom (right).

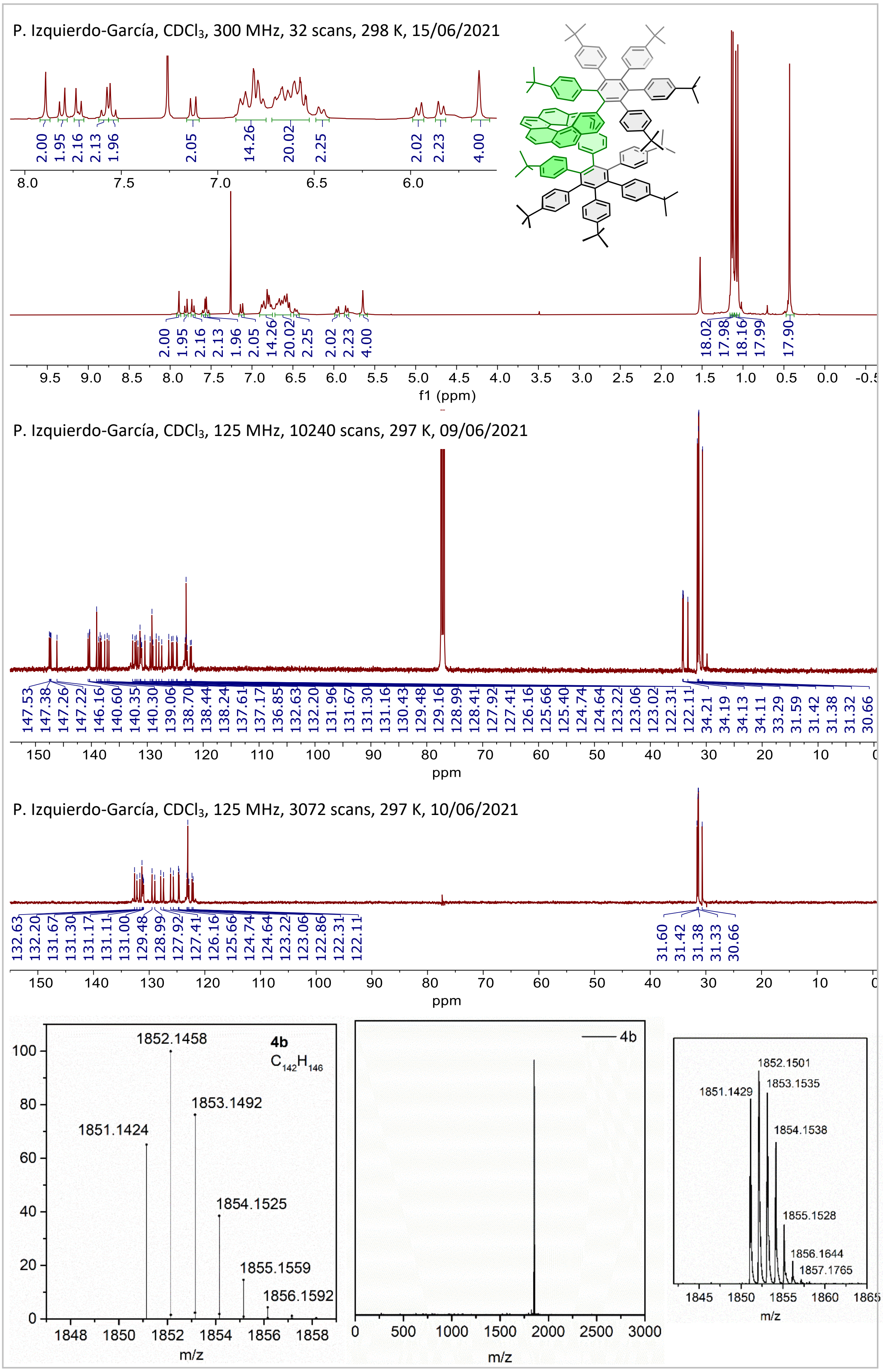

Figure S6. Compound ***rac*-4b** NMR spectra: ^{1}H, ^{13}C and DEPT and mass spectra: theoretical (left), MALDI-ToF (middle) and peak zoom (right).

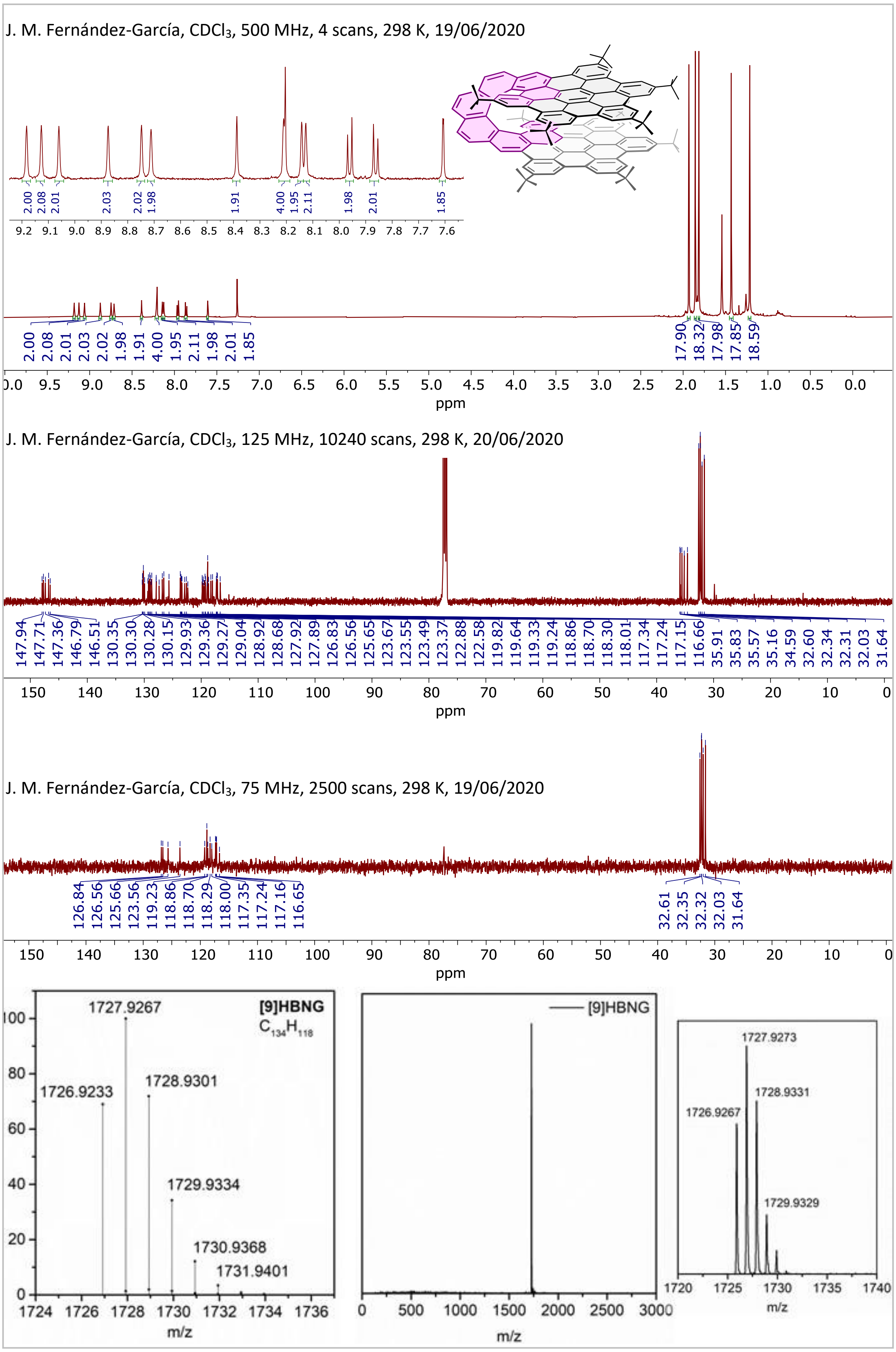

Figure S7. Compound **[9]HBNG** NMR spectra: ^{1}H, ^{13}C and DEPT and mass spectra: theoretical (left), MALDI-ToF (middle) and peak zoom (right).

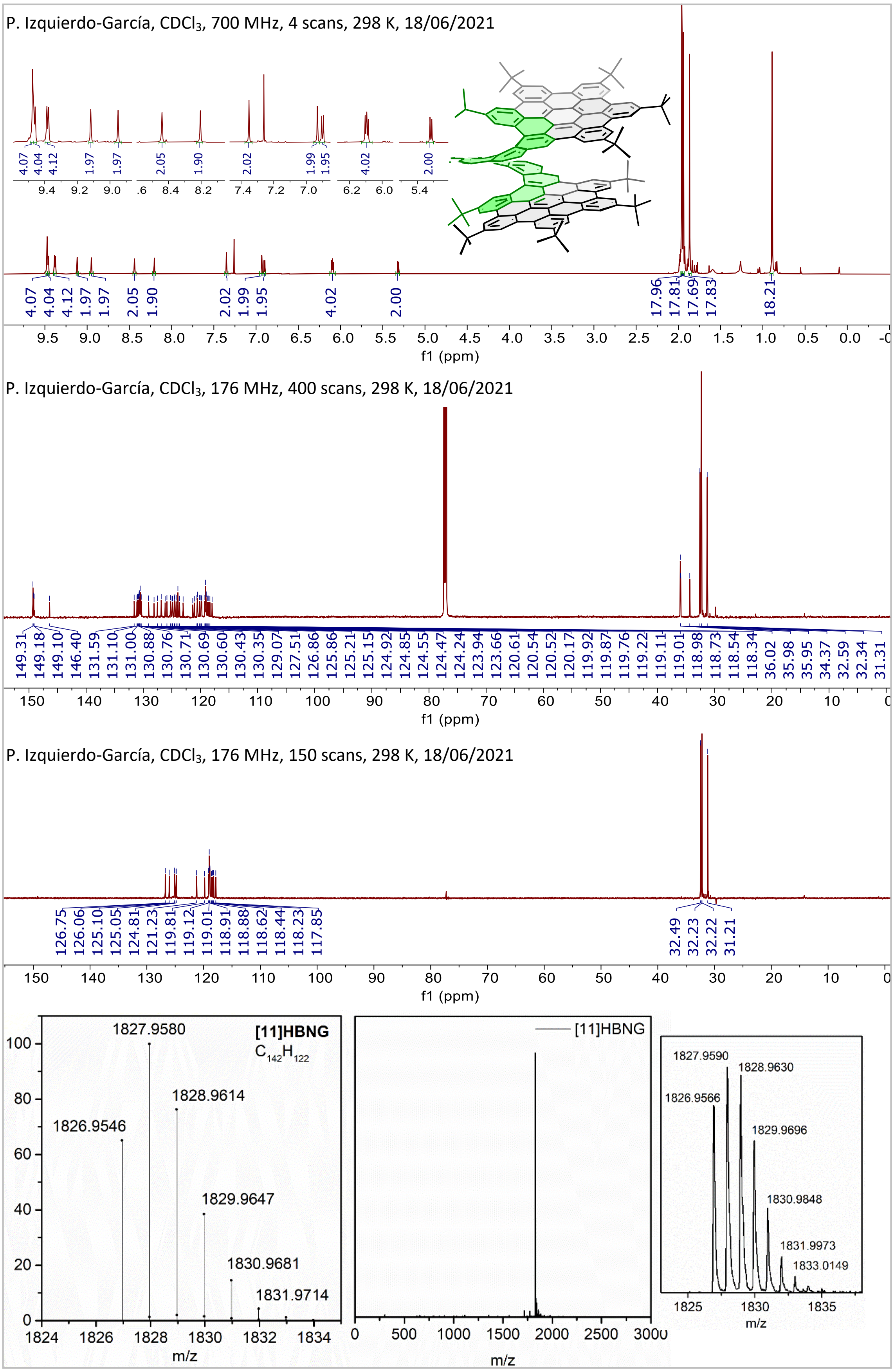

Figure S8. Compound **[11]HBNG** NMR spectra: ^{1}H, ^{13}C and DEPT and mass spectra: theoretical (left), MALDI-ToF (middle) and peak zoom (right).

4. Crystal structure description for compounds [9]HBNG and [11]HBNG and comparison with [10]HBNG

Single crystals suitable for X ray diffraction analysis were obtained from the respective racemic mixtures containing *P* and *M* isomers of compounds **[9]HBNG** and **[11]HBNG**. In both cases, the resulting crystals belonged to the centrosymmetric triclinic *P*-1 space group, and consequently contained both isomers.

For both nanographenes, the packing displays voids between the HBNGs that contain highly disordered solvent molecules; some of them could be modelled as oxygen atoms from water molecules in **[9]HBNG**, and dichloroethane and oxygen atoms from water in **[11]HBNG**. The crystals were found to be extremely unstable out of their mother liquors due to the loss of these interstitial solvent molecules. Additionally, many of the *tert*-butyl groups are also heavily disordered and were modelled with two alternative sets of positions.

Table S1. Sample and crystal data for **[9]HBNG.**

**CCDC code**	2233478	
**Chemical formula**	$(C_{134}H_{118})\cdot 4.38\ H_2O$	
**Formula weight**	1807.30 g/mol	
**Temperature**	250(2) K	
**Wavelength**	0.71073 Å	
**Crystal size**	0.026 x 0.236 x 0.507 mm	
**Crystal habit**	dark brown-orange plate	
**Crystal system**	triclinic	
**Space group**	*P* -1	
**Unit cell dimensions**	*a* = 13.382(4) Å	α = 98.887(11)°
	b = 17.848(5) Å	β = 92.864(13)°
	c = 28.398(8) Å	γ = 109.273(11)°
**Volume**	6288.(3) $Å^3$	
**Z**	2	
**Density (calculated)**	0.955 g/cm^3	
**Absorption coefficient**	0.056 mm^{-1}	
**F(000)**	1914	

Table S2. Data collection and structure refinement for **[9]HBNG.**

**Theta range for data collection**	1.54 to 25.36°
**Index ranges**	-15<=h<=15, -20<=k<=21, -34<=l<=34
**Reflections collected**	217729
**Independent reflections**	22608 [R(int) = 0.0631]
**Coverage of independent reflections**	98.0%

**Absorption correction**	Multi-Scan	
**Max. and min. transmission**	1.0000 and 0.9300	
**Refinement method**	Full-matrix least-squares on F^2	
**Refinement program**	SHELXL-2019/1 (Sheldrick, 2019)	
**Function minimized**	$\Sigma w(F_o^2 - F_c^2)^2$	
**Data / restraints / parameters**	22608 / 1461 / 1386	
**Goodness-of-fit on F^2**	1.028	
**Final R indices**	13523 data; I>2σ(I)	$R_1 = 0.1277$, $wR_2 = 0.3571$
	all data	$R_1 = 0.1900$, $wR_2 = 0.4151$
**Weighting scheme**	$w=1/[\sigma^2(F_o^2)+(0.2000P)^2+22.7000P]$; where $P=(F_o^2+2F_c^2)/3$	
**Largest diff. peak and hole**	1.687 and -0.662 eÅ^{-3}	
**R.M.S. deviation from mean**	0.251 eÅ^{-3}	

Table S3. Sample and crystal data for **[11]HBNG.**

**CCDC code**	2233479	
**Chemical formula**	$(C_{142}H_{122})\cdot1.5(C_2H_4Cl_2)\cdot5\ H_2O$	
**Formula weight**	2091.02 g/mol	
**Temperature**	150(2) K	
**Wavelength**	0.71073 Å	
**Crystal size**	0.189 x 0.223 x 0.235 mm	
**Crystal habit**	dark brown prism	
**Crystal system**	triclinic	
**Space group**	*P* -1	
**Unit cell dimensions**	*a* = 18.7384(8) Å	α = 111.0735(15)°
	b = 20.1754(8) Å	β = 108.7063(15)°
	c = 20.9971(8) Å	γ = 102.5524(16)°
**Volume**	6500.0(5) Å^3	
**Z**	2	
**Density (calculated)**	1.069 g/cm^3	
**Absorption coefficient**	0.126 mm^{-1}	
**F(000)**	2182	

Table S4. Data collection and structure refinement for **[11]HBNG.**

**Theta range for data collection**	1.68 to 25.35°
**Index ranges**	-22<=h<=22, -24<=k<=24, -25<=l<=25
**Reflections collected**	101650
**Independent reflections**	23790 [R(int) = 0.0383]
**Coverage of independent reflections**	99.9%
**Absorption correction**	Multi-Scan
**Max. and min. transmission**	0.9760 and 0.9710
**Structure solution technique**	direct methods
**Structure solution program**	XT, VERSION 2018/2
**Refinement method**	Full-matrix least-squares on F^2
**Refinement program**	SHELXL-2019/1 (Sheldrick, 2019)
**Function minimized**	$\Sigma w(F_o^2 - F_c^2)^2$
**Data / restraints / parameters**	23790 / 1542 / 1538
**Goodness-of-fit on F^2**	1.052
**Final R indices**	15860 data; I>2σ(I) $R_1 = 0.1182$, $wR_2 = 0.3678$
	all data $R_1 = 0.1630$, $wR_2 = 0.4228$
**Weighting scheme**	$w=1/[\sigma^2(F_o^2)+(0.3000P)^2+7.9001P]$; where $P=(F_o^2+2F_c^2)/3$
**Largest diff. peak and hole**	2.195 and -0.667 eÅ^{-3}
**R.M.S. deviation from mean**	0.220 eÅ^{-3}

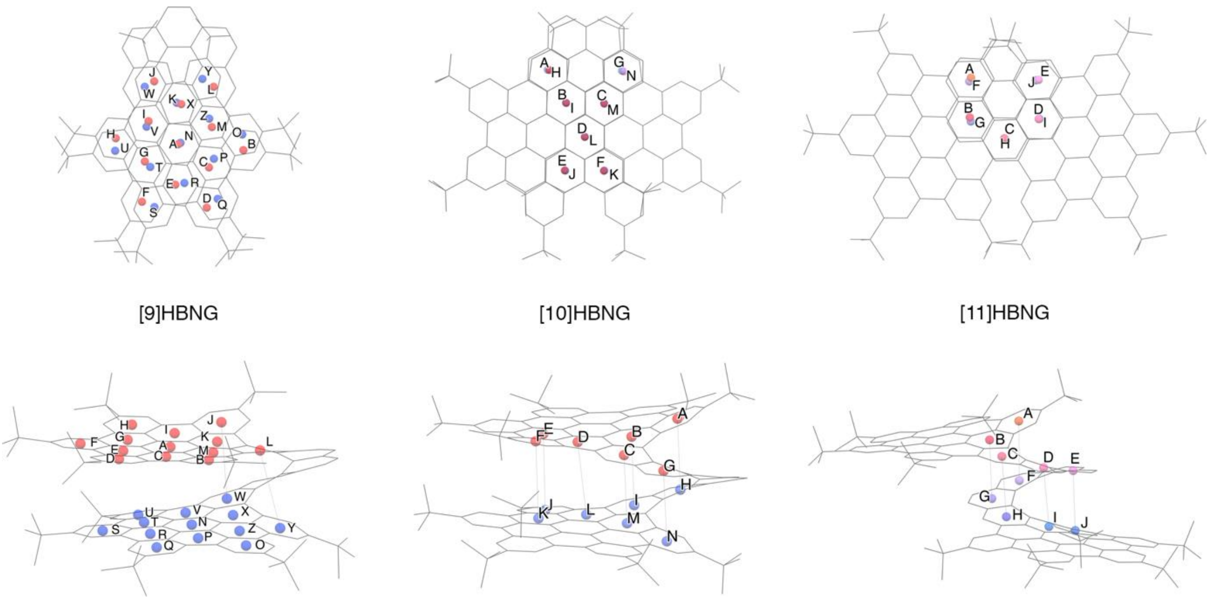

Figure S9. Centroids in the overlapping rings in molecules **[9]HBNG, [10]HBNG** and **[11]HBNG**. Top, zenithal view; bottom, lateral view.

Table S5. Relevant distances for intermolecular interactions in **[9]HBNG, [10]HBNG** and **[11]HBNG.** In the case of compound **[9]HBNG**, the distances were measured from the centroid of a ring to the plane in the opposite layer, calculated as the mean plane for the central ring at each HBC and the six adjacent ones (ie: rings with centroids ACEGIKM for plane 1 and NPRTVXZ for plane 2) except for those rings that were bent out of their respective planes (with centroids labelled J and Y).

Distance between centroids and opposite plane (Å)				
**[9]HBNG**	A—plane 2	3.490	N—plane 1	3.526
	B—plane 2	3.821	O—plane1	3.749
	C—plane 2	3.660	P—plane 1	3.615
	D—plane 2	3.875	Q—plane 1	3.728
	E—plane 2	3.622	R—plane 1	3.630
	F—plane 2	3.649	S—plane 1	3.711
	G—plane 2	3.512	T—plane 1	3.644
	H—plane 2	3.600	U—plane 1	3.838
	I—plane 2	3.509	V—plane 1	3.512
	J—W*		3.648	
	K—plane 2	3.412	X—plane 1	3.397
	L—Y*		3.822	
	M—plane 2	3.535	Z—plane 1	3.604
	In the case of the pairs of centroids J-W and L-Y, the distances were measured centroid-to-centroid because the rings containing J and W are bent out of plane (distance J-plane 1 = 0.349 Å; Y-plane 2 = 0.362 Å).			
**Distance between centroids (Å)**				
**[10]HBNG**	A—H		3.508	
	B—I		3.368	
	C—M		3.355	
	D—L		3.580	
	E—J		3.738	
	F—K		3.781	
	G—N		3.481	
**[11]HBNG**	A—F		3.404	
	B—G		3.330	
	C—H		3.445	
	D—I		3.390	
	E—J		3.472	

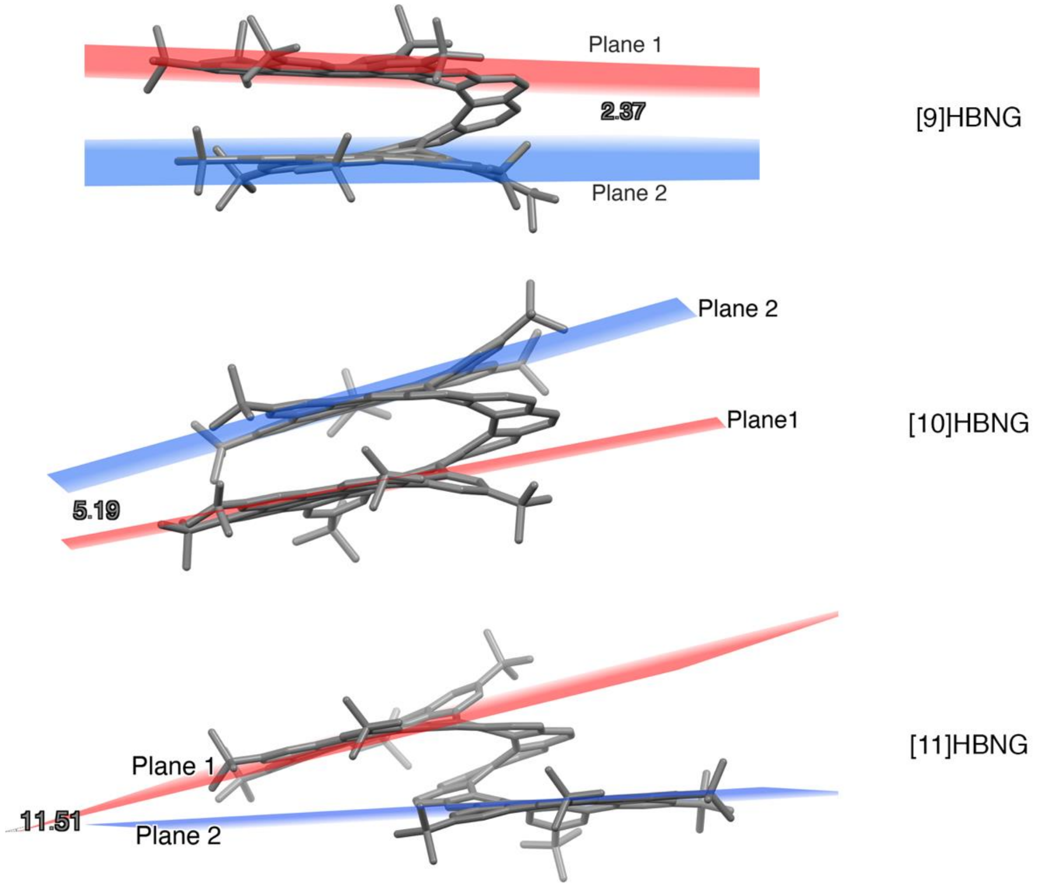

Figure S10. Angles between planes in **[9]HBNG, [10]HBNG** and **[11]HBNG**, calculated as the mean planes considering the central ring and the six adjacent ones from each layer.

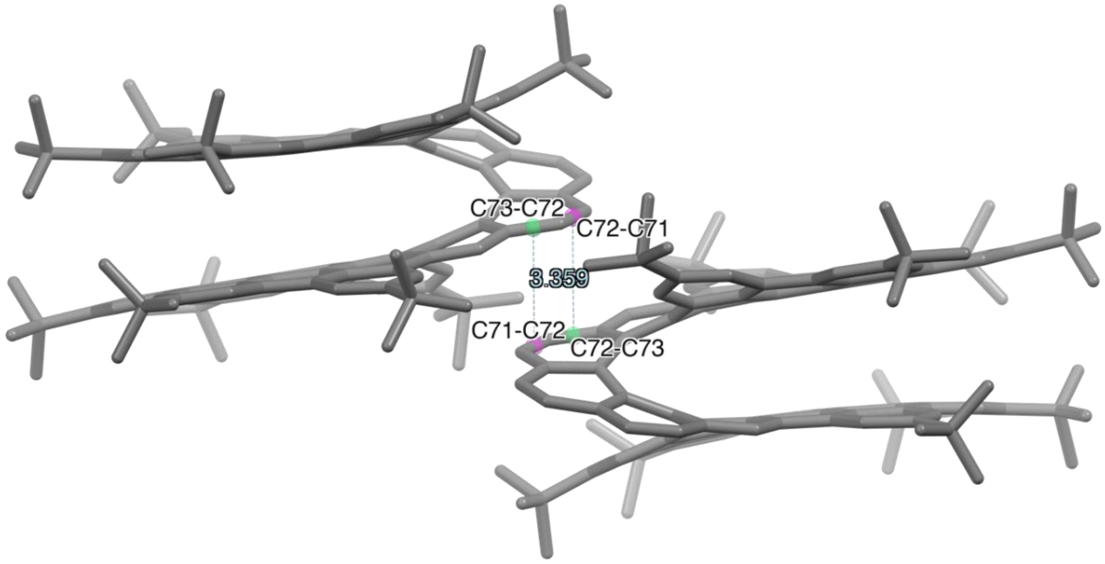

Figure S11. Distance between centroids of C-C bonds in a pair of neighbor M and P **[9]HBNG** molecules in the [100] direction.

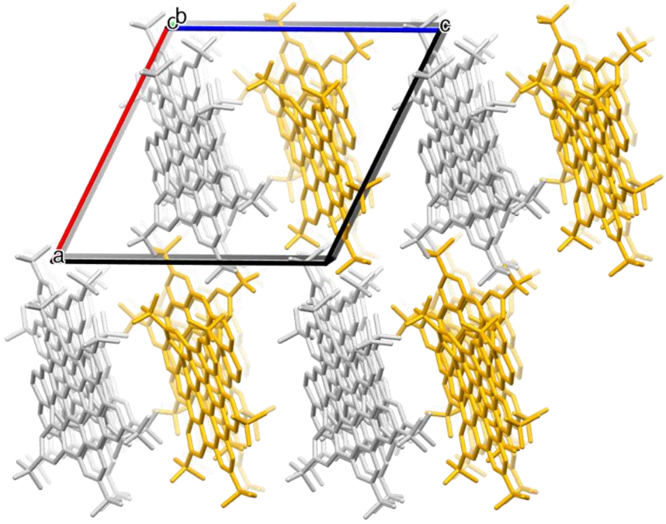

Figure S12. Packing of homochiral columns of M and P enantiomers of **[11]HBNG** molecules (depicted in grey and yellow respectively) in the [010] direction.

5. Electrochemical measurements

Electrochemical measurements were performed using a standard one-compartment, three-electrode electrochemical cell connected to an electrochemical analyzer (Metrohm Autolab). The working electrode was a glassy carbon electrode (3 mm diameter) that was freshly polished with a suspension of Al_2O_3 in distilled water and sonically rinsed with acetone before each measurement. Silver (Ag/0.1 M $AgNO_3$ in CH_3CN) and platinum wires were used as reference and counter electrodes, respectively. Electrochemical grade (Aldrich) tetrabutylammonium hexaflurophosphate 0.1 M in toluene:acetonitrile (4:1) was used as supporting electrolyte. All measurements were conducted under dry argon. Solutions were saturated with argon for deoxygenation and to maintain an argon blanket for at least 10 minutes prior to each measurement. All measurements are referenced to Fc/Fc^+ added as internal reference.

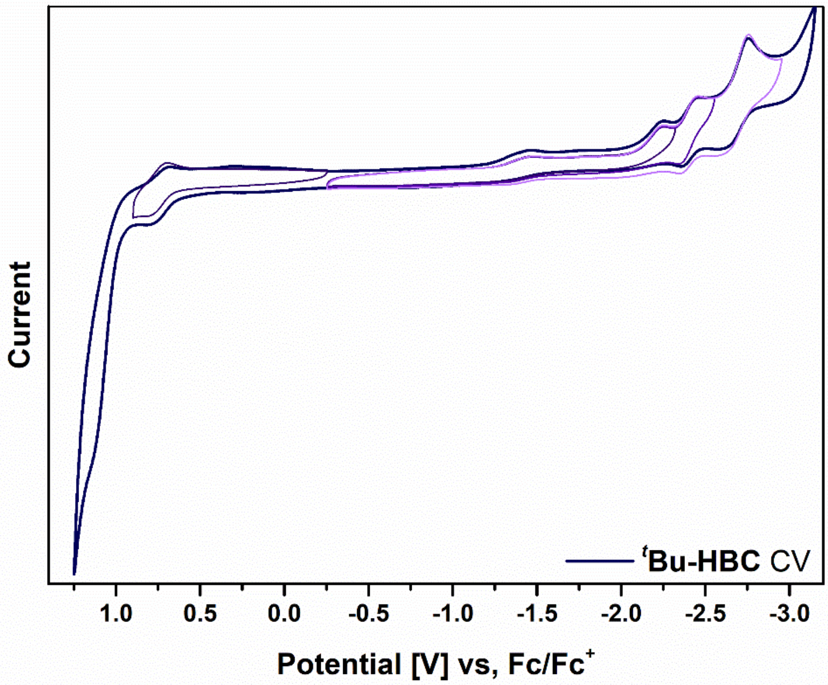

Figure S13. Cyclic voltammograms of ***t*****Bu-HBC**.

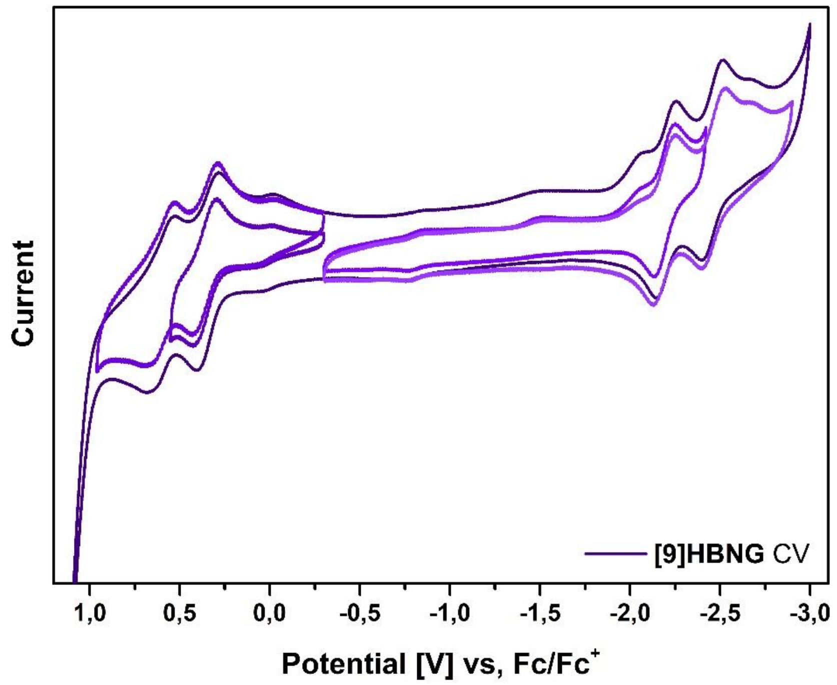

Figure S14. Cyclic voltammograms of **[9]HBNG**.

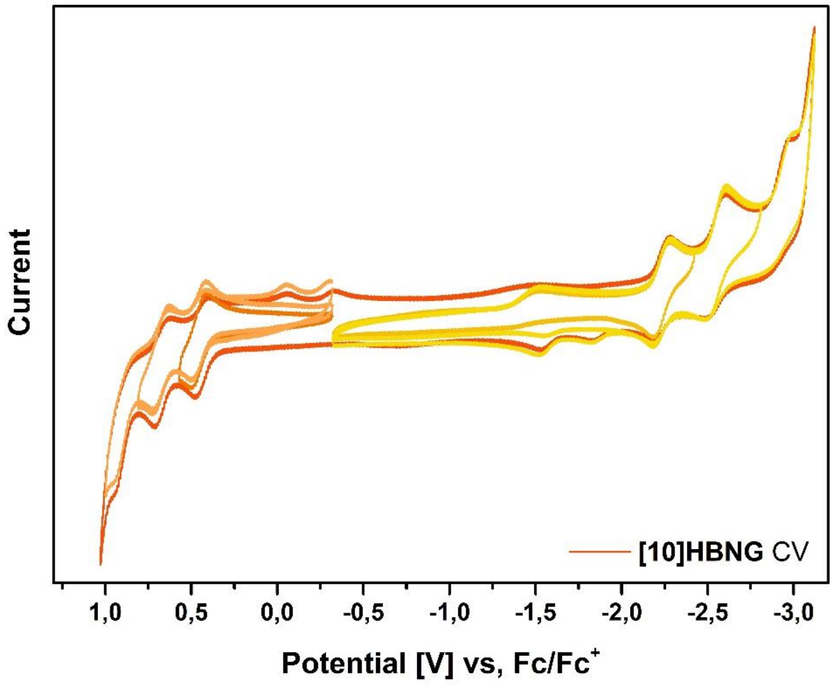

Figure S15. Cyclic voltammograms of **[10]HBNG**.

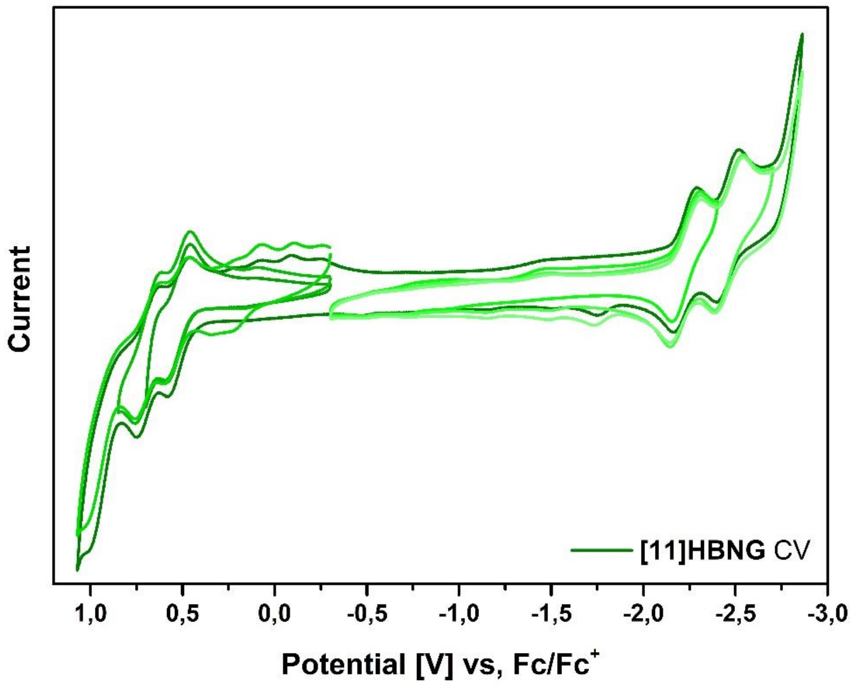

Figure S16. Cyclic voltammograms of **[11]HBNG**.

6. UV-vis-NIR spectroelectrochemistry

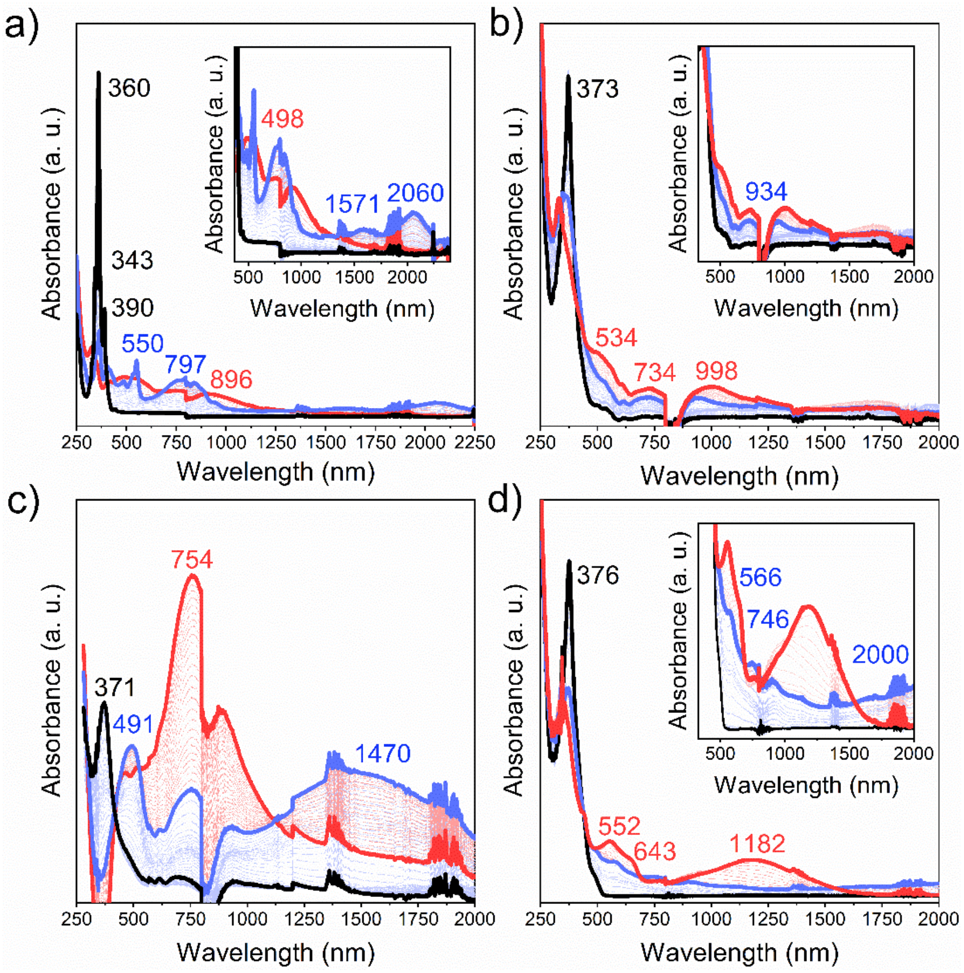

Figure S17. UV-Vis-NIR electronic absorption spectra obtained upon electrochemical oxidation of a) **^{t}Bu-HBC**, b) **[9]HBNG**, c) **[10]HBNG**, and d) **[11]HBNG**, in a 0.1 M solution of Bu_4NPF_6 in CH_2Cl_2 at room temperature. Black lines correspond to neutral species, blue lines correspond to the first oxidized species and red lines correspond to the second oxidized species. Light colour lines correspond to the intermediate spectra of the former species.

7. Photophysical characterization

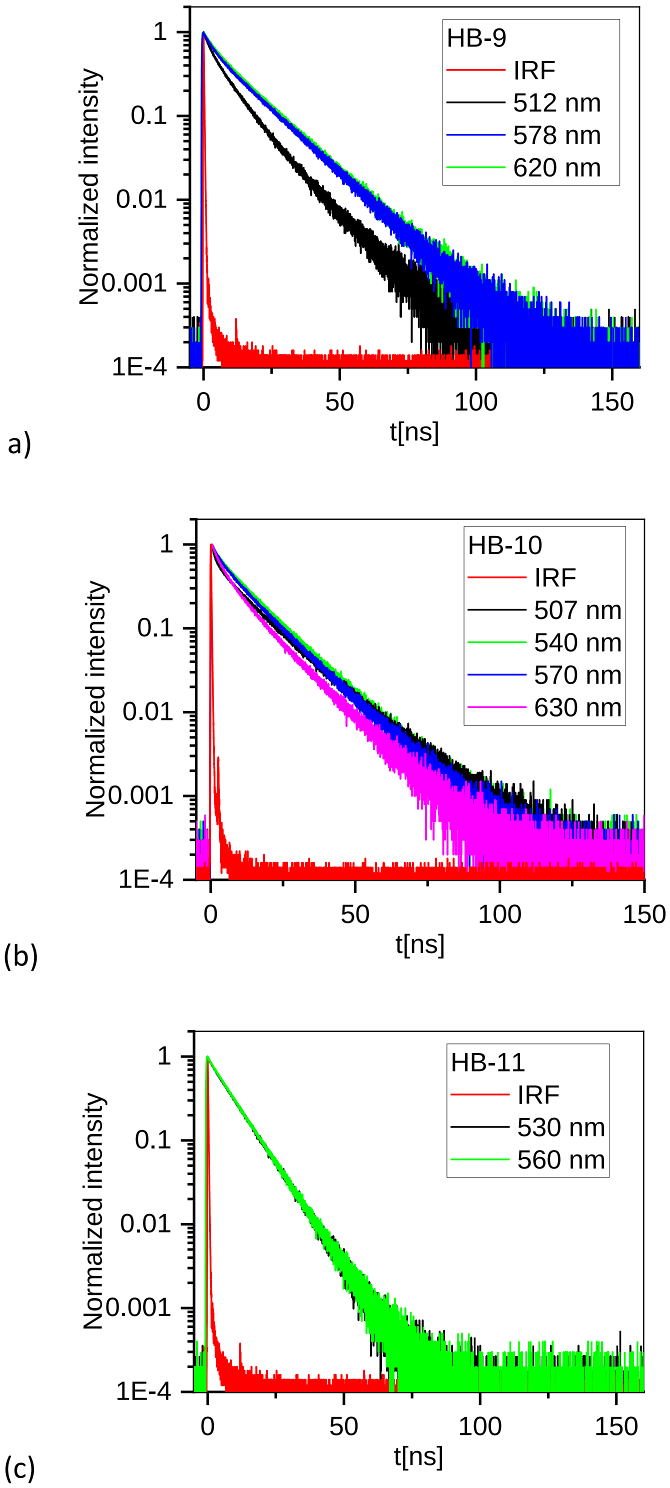

Figure S18. Fluorescence decay profiles of helical bilayer nanographenes **[9]HBNG** (a), **[10]HBNG** (b) and **[11]HBNG** (c) in chloroform, obtained at different emission detection wavelengths with excitation at 405 nm. Instrument response functions (IRF) are shown in red.

Table S6. Data from the (multi)exponential fittings of the fluorescence decays of helical bilayer nanographenes **[9]HBNG**, **[10]HBNG** and **[11]HBNG**, obtained at different emission detection wavelengths (peaks or shoulders, sh) with excitation at 405 nm.

**Compound**	$\lambda_{detection}$ (nm)	τ_1 **(ns)**	B_1	I_1	τ_2 **(ns)**	B_2	I_2	τ_3 **(ns)**	B_3	I_3	τ_{Int} **(ns)**	τ_{Amp} **(ns)**
**[9]HBNG**												
	512 (sh)	11.2	0.51	0.79	3.2	0.49	0.21	-	-	-	9.5	7.3
λ_{em}^{max} = 575 nm	578 (peak)	**14.3**	0.71	0.91	**3.2**	0.29	0.09	-	-	-	**13.4**	**11.1**
	620 (sh)	14.3	0.66	0.90	3.1	0.34	0.10	-	-	-	13.2	10.5
**[10]HBNG**												
	507 (sh)	14.6	0.31	0.78	5.1	0.20	0.18	0.52	0.48	0.04	12.3	5.8
λ_{em}^{max} = 543 nm	540 (peak)	**13.2**	0.40	0.90	**3.4**	0.15	0.09	**0.11**	0.45	0.01	**12.2**	**5.8**
	570 (sh)	13.0	0.48	0.86	3.9	0.24	0.13	0.29	0.28	0.01	11.7	7.3
	630 (sh)	12.3	0.25	0.75	3.7	0.28	0.24	0.12	0.47	0.01	10.1	4.2
**[11]HBNG**												
λ_{em}^{max} = 528 nm	530 (peak)	**8.7**	1.0	-	-	-	-	-	-	-	-	-
	560 (sh)	8.7	1.0	-	-	-	-	-	-	-	-	-

Table S7. Estimated radiative (k_r) and nonradiative (k_{nr}) deactivation rate constants of the singlet excited states calculated with data from the (multi)exponential fitting of the fluorescence decays of the helical bilayer nanographenes **[9]HBNG**, **[10]HBNG** and **[11]HBNG**, and their respective emission quantum yields.

**Compound**	τ_{Amp} **(ns)**	Φ_{em}	$k_r^{S1\rightarrow S0}$ **(s^{-1})^a**	$k_{nr}^{S1\rightarrow S0}$ **(s^{-1})^b**
**[9]HBNG**	11.1	0.22	2.0×10^7	7.0×10^7
**[10]HBNG**	5.8	0.10	1.7×10^7	1.5×10^8
**[11]HBNG**	8.7	0.11	1.3×10^7	1.0×10^8

$^a k_r^{S1\rightarrow S0} = \Phi_{em}/\tau_{em}$. $^b k_{nr}^{S1\rightarrow S0} = (1/\tau_{em}) - k_r^{S1\rightarrow S0}$

8. Vibrational Raman spectroscopy

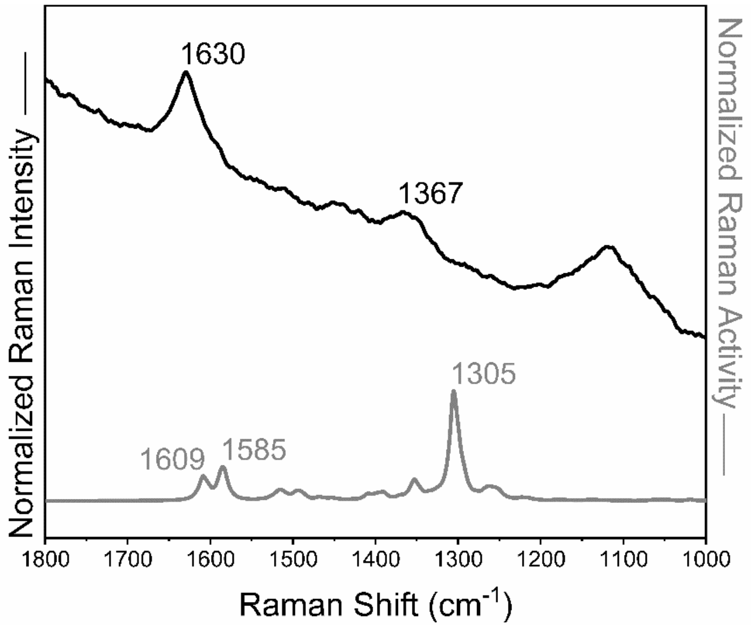

Figure S19. Experimental (black solid line; λ_{exc} = 325 nm) and theoretical (grey solid line) Raman spectra of **[9]HBNG**. Theoretical Raman spectrum was calculated at the optimized CAM-B3LYP/3-21G* geometry and scaled down uniformly by a factor of 0.96.

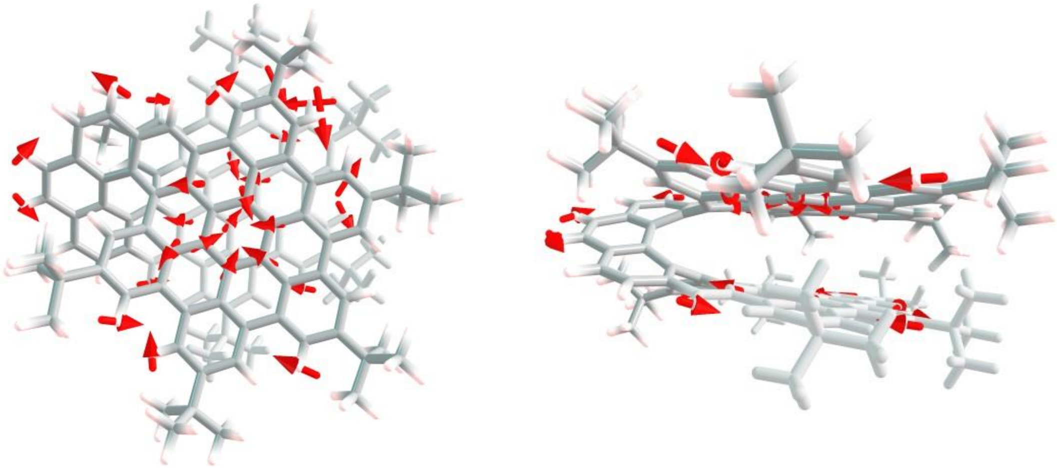

D Band 1305 cm^{-1}

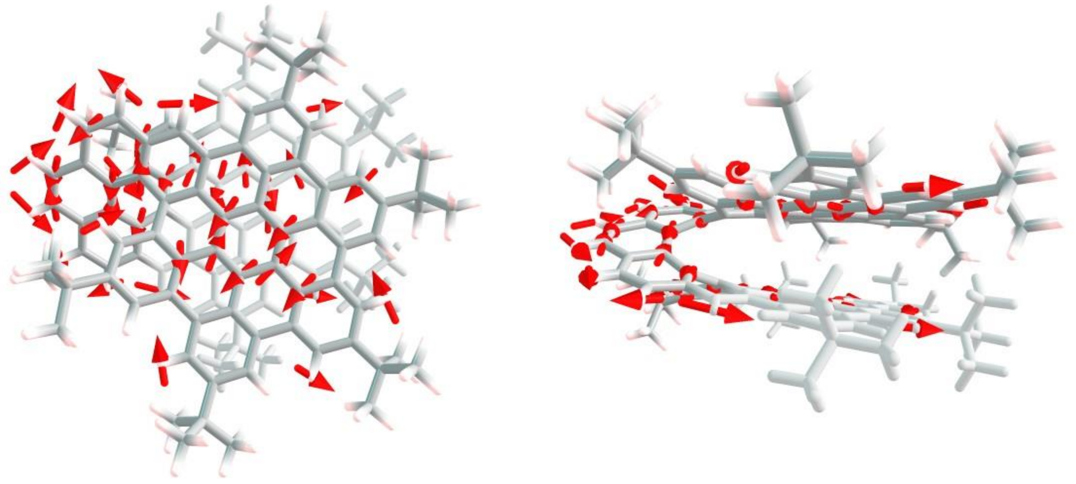

G Band 1585 cm^{-1}

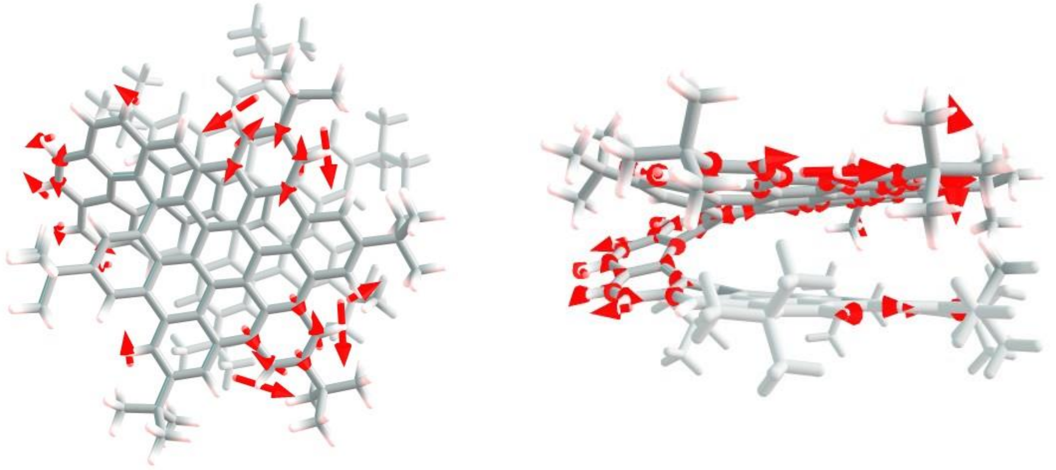

G Band 1609 cm^{-1}

Figure S20. Vibrational normal coordinates corresponding to G and D bands in the computed Raman spectrum of **[9]HBNG** (**Figure S19**). Bottom graphene layer is showed in light grey for clarity.

9. HPLC resolution and spectral characterization

Racemic bilayer helicenes (prepared from [5]helicene precursor – **[9]HBNG**, [6]helicene precursor – **[10]HBNG**, and [7]helicene precursor – **[11]HBNG**) were resolved by means of semipreparative CSP HPLC using Chiralpak IE column using a mixture of heptane and 1% isopropyl alcohol in toluene as mobile phase. Unfortunately, a high loss of material was observed during the chromatographic separations, which indicates either decomposition or irreversible adhesion on the column. Below, the experimental conditions of the HPLC separations and characterization of the obtained enantioenriched samples are summarized.

Analytical separations were performed on Chiralpak IE (250 x 4.6 mm, 5 µm, DAICEL) column using isocratic elution with heptane – 5% THF in toluene 60:40 at 1 mL/min, semi-preparative separations were done using a Chiralpak IE (250 x 20 mm, 5 µm, DAICEL) column and a gradient elution with heptane – 1% isopropyl alcohol in toluene [80:20] to [50:50] at 20 mL/min. Injections of cca 10 mg / 1.5 mL of the racemic material in a mixture of 1,1,2,2-tetrachloroethane and heptane (1:2) per run were performed.

<u>Racemic mixtures:</u>

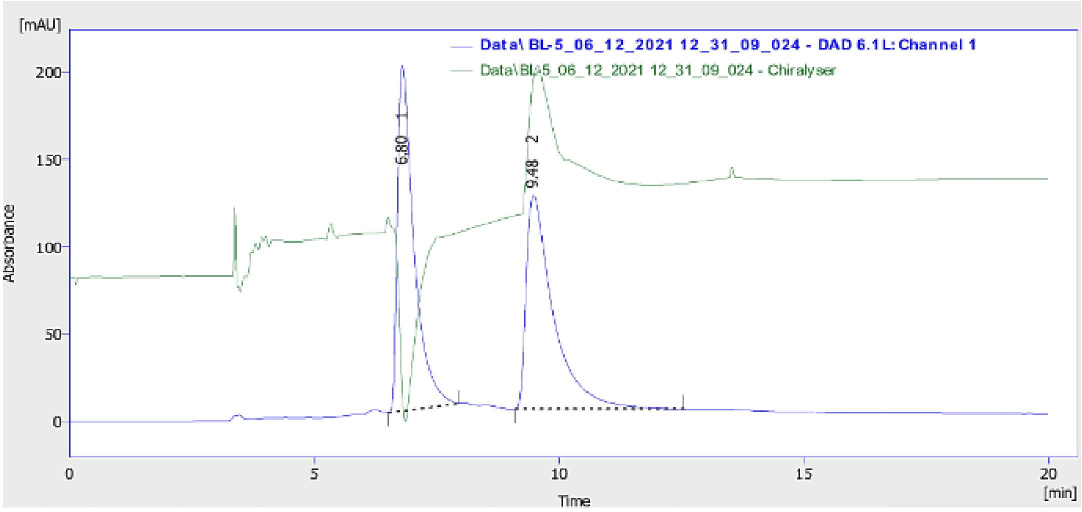

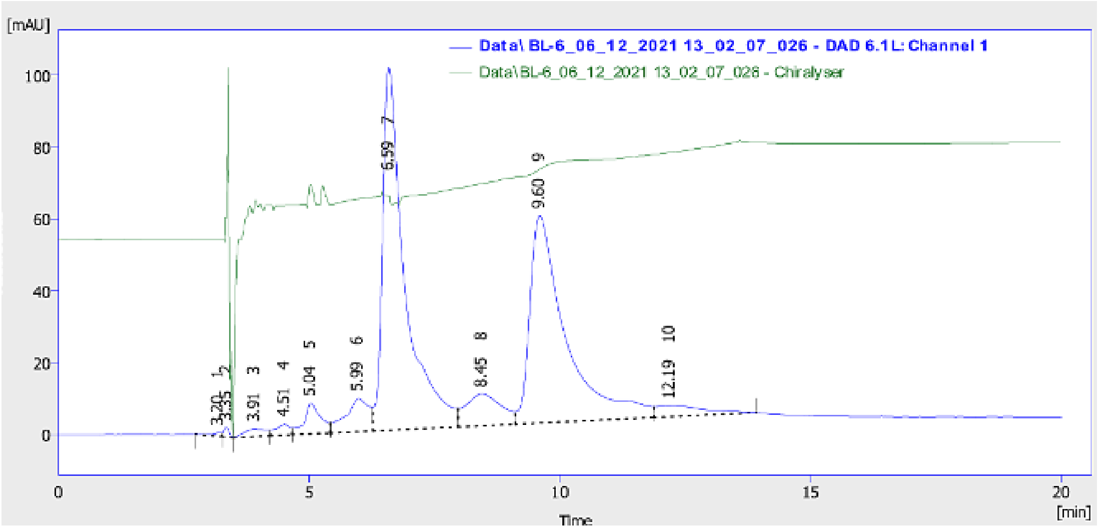

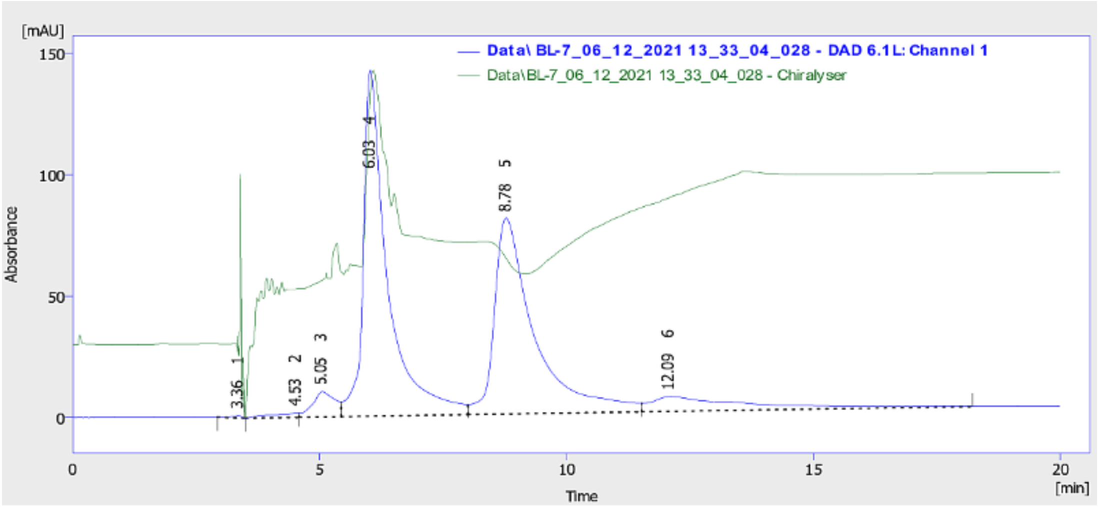

Figure S21. Chromatograms of **[9]HBNG**, **[10]HBNG** and **[11]HBNG** as racemic mixtures respectively.

<u>After resolution:</u>

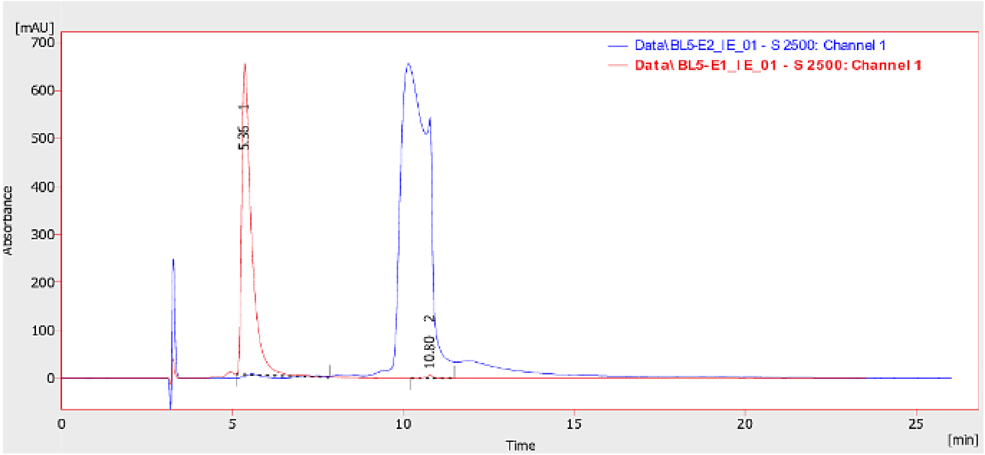

Result Table (Uncal - Data\BL5-E2_IE_01 - S 2500: Channel 1)

	Reten. Time [min]	Area [mAU.s]	Height [mAU]	Area [%]	Height [%]	W05 [min]	Compound Name
1	5.590	45.691	1.694	0.7	1.3	0.39	
2	10.157	6838.477	127.304	99.3	98.7	0.97	
	Total	6884.168	128.998	100.0	100.0		

Result Table (Uncal - Data\BL5-E2_IE_01 - RI detector)

Reten. Time [min]	Area [V.s]	Height [V]	Area [%]	Height [%]	W05 [min]	Compound Name
No peak to report						

Result Table (Uncal - Data\BL5-E1_IE_01 - S 2500: Channel 1)

	Reten. Time [min]	Area [mAU.s]	Height [mAU]	Area [%]	Height [%]	W05 [min]	Compound Name
1	5.363	12895.413	648.494	99.4	99.0	0.28	
2	10.797	79.619	6.364	0.6	1.0	0.14	
	Total	12975.032	654.858	100.0	100.0		

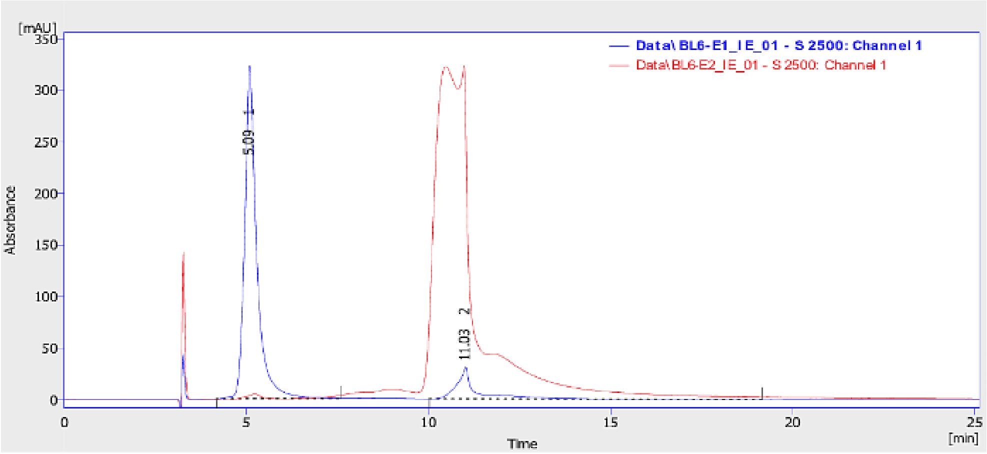

Result Table (Uncal - Data\BL6-E1_IE_01 - S 2500: Channel 1)

	Reten. Time [min]	Area [mAU.s]	Height [mAU]	Area [%]	Height [%]	W05 [min]	Compound Name
1	5.093	7411.494	322.723	86.5	91.3	0.30	
2	11.027	1154.289	30.939	13.5	8.7	0.30	
	Total	8565.783	353.661	100.0	100.0		

Result Table (Uncal - Data\BL6-E1_IE_01 - RI detector)

Reten. Time [min]	Area [V.s]	Height [V]	Area [%]	Height [%]	W05 [min]	Compound Name
No peak to report						

Result Table (Uncal - Data\BL6-E2_IE_01 - S 2500: Channel 1)

	Reten. Time [min]	Area [mAU.s]	Height [mAU]	Area [%]	Height [%]	W05 [min]	Compound Name
1	5.230	48.968	1.640	0.7	1.5	0.44	
2	10.980	7394.899	104.423	99.3	98.5	0.96	
	Total	7443.867	106.063	100.0	100.0		

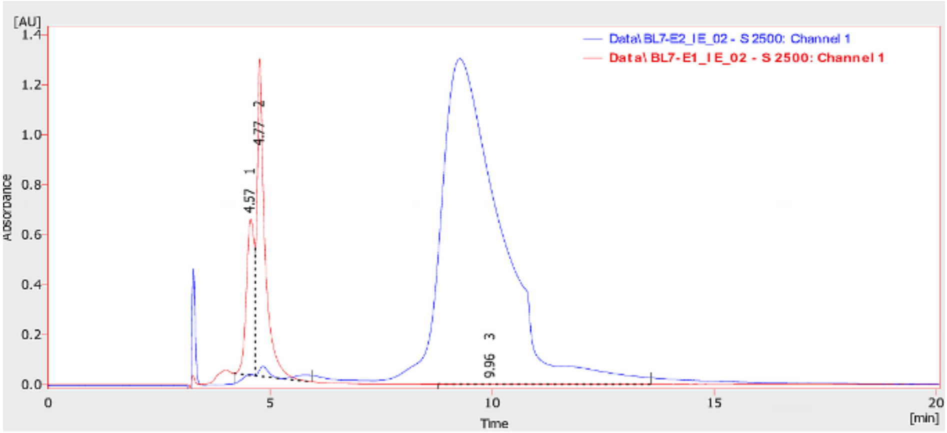

Result Table (Uncal - Data\BL7-E2_IE_02 - S 2500: Channel 1)

	Reten. Time [min]	Area [mAU.s]	Height [mAU]	Area [%]	Height [%]	W05 [min]	Compound Name
1	4.550	53.539	3.176	0.4	2.3	0.27	
2	4.637	99.634	5.811	0.8	4.1	0.28	
3	9.270	12150.342	131.962	98.8	93.6	1.31	
	Total	12303.515	140.949	100.0	100.0		

Result Table (Uncal - Data\BL7-E2_IE_02 - RI detector)

Reten. Time [min]	Area [V.s]	Height [V]	Area [%]	Height [%]	W05 [min]	Compound Name
No peak to report						

Result Table (Uncal - Data\BL7-E1_IE_02 - S 2500: Channel 1)

	Reten. Time [min]	Area [mAU.s]	Height [mAU]	Area [%]	Height [%]	W05 [min]	Compound Name
1	4.567	7607.123	625.851	32.4	33.0	0.20	
2	4.767	15765.669	1270.064	67.1	66.9	0.16	
3	9.963	127.576	1.323	0.5	0.1	1.44	
	Total	23500.357	1897.237	100.0	100.0		

Figure S22. Chromatograms of **[9]HBNG**, **[10]HBNG** and **[11]HBNG** after resolution, respectively.

Table S8. Material balance and optical rotation values of the enantioenriched samples

Compound	Original sample weight (mg)	Amount of sample after resolution (mg)	Optical rotation[a] $[\alpha]_D^{20}$	HPLC enantiomeric purity *ee* (%)	NMR purity[b] (wt%)
(+)-[9]HBNG	30	8	+6706	98	96
(-)-[9]HBNG		8	-7120	99	97
(+)-[10]HBNG	20	3	+2447	73	94
(-)-[10]HBNG		3	-4480	99	93
(+)-[11]HBNG	30	8	+4033	99	92
(-)-[11]HBNG		8	-4270	99	92

[a] Optical rotations were measured in THF solutions (c = 0.078 – 0.095 g/100mL)

[b] Despite drying *in vacuo*, residual solvents (mainly toluene and/or dichloromethane) were found to be present in the resolved samples by ^{1}H NMR (in THF-d_8).

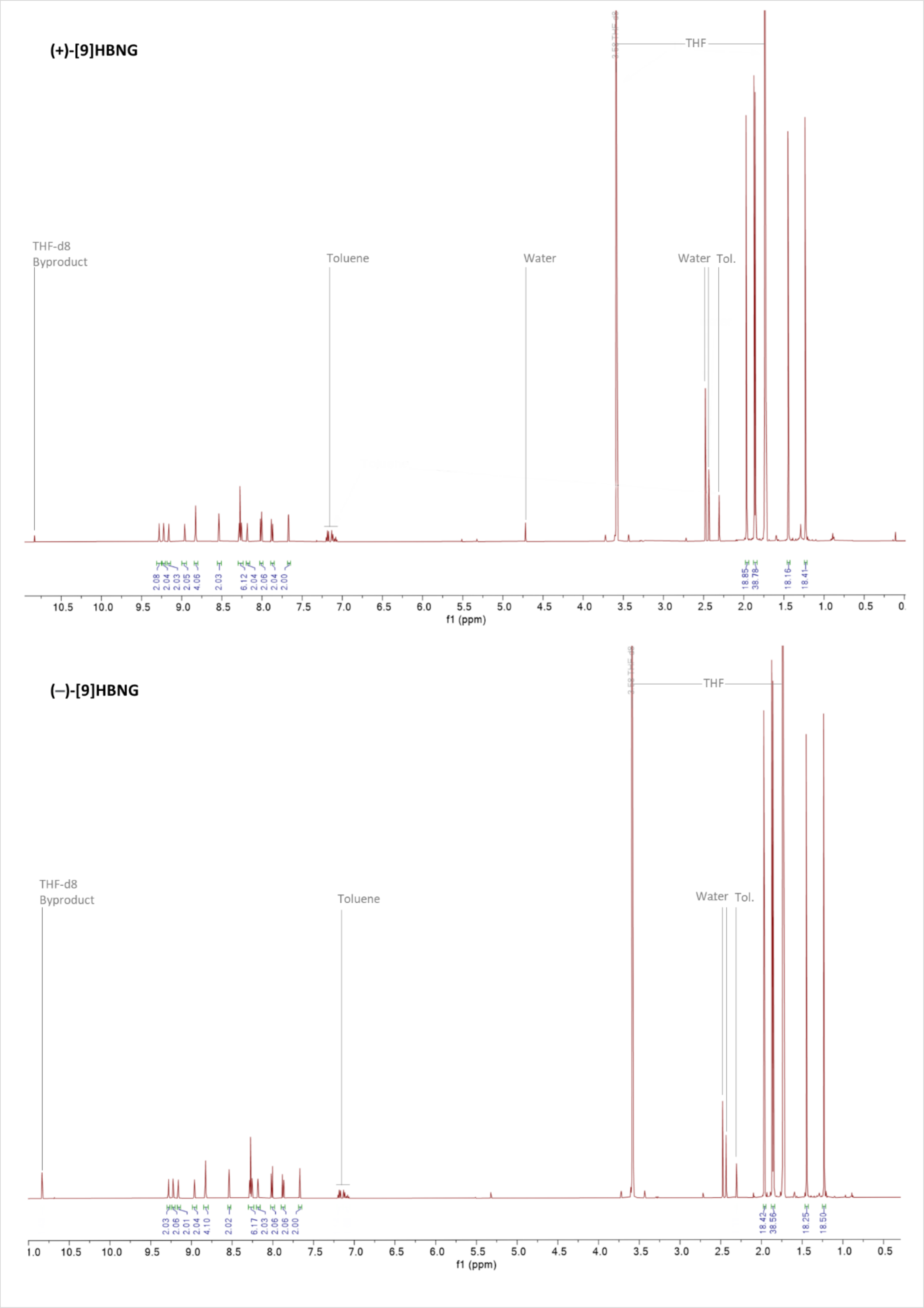

(+)-[9]HBNG
THF
THF-d8
Byproduct
Toluene
Water
Water
Tol.
f1 (ppm)
(–)-[9]HBNG
THF
THF-d8
Byproduct
Toluene
Water
Tol.
f1 (ppm)

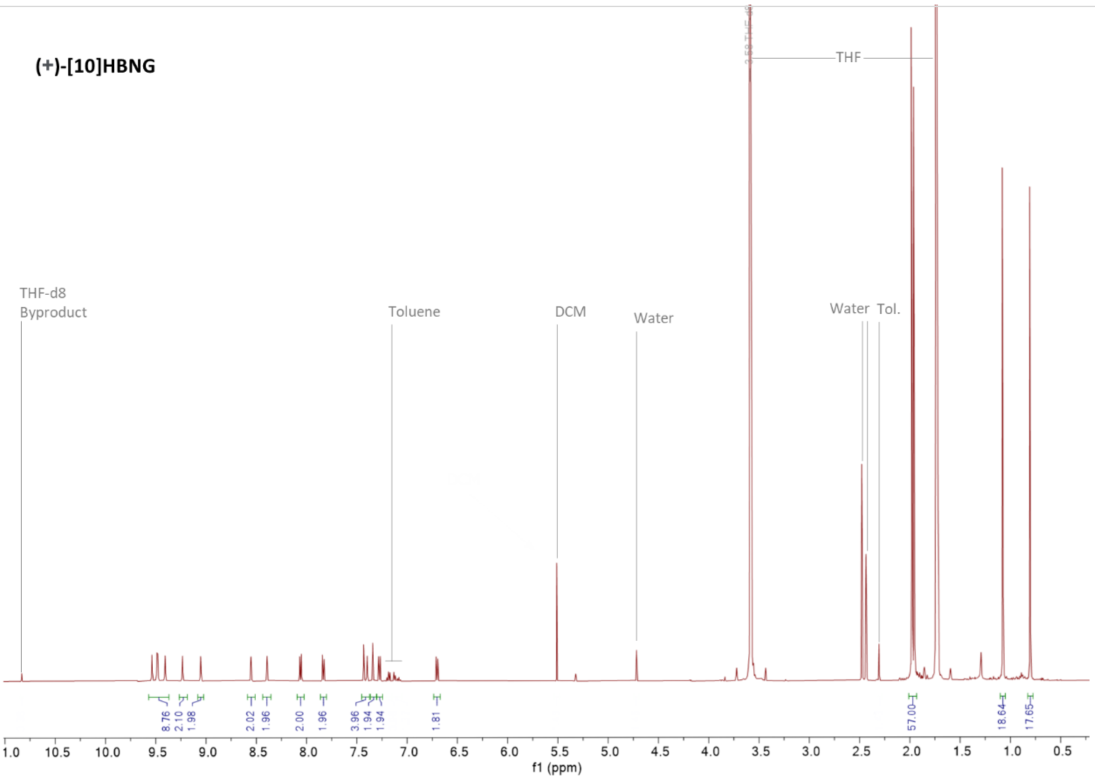
(+)-[10]HBNG
THF
THF-d8
Byproduct
Toluene
DCM
Water
Water Tol.
8.76
2.10
1.98
2.02
1.96
2.00
1.96
3.96
1.94
1.94
1.81
57.00
18.64
17.65
f1 (ppm)

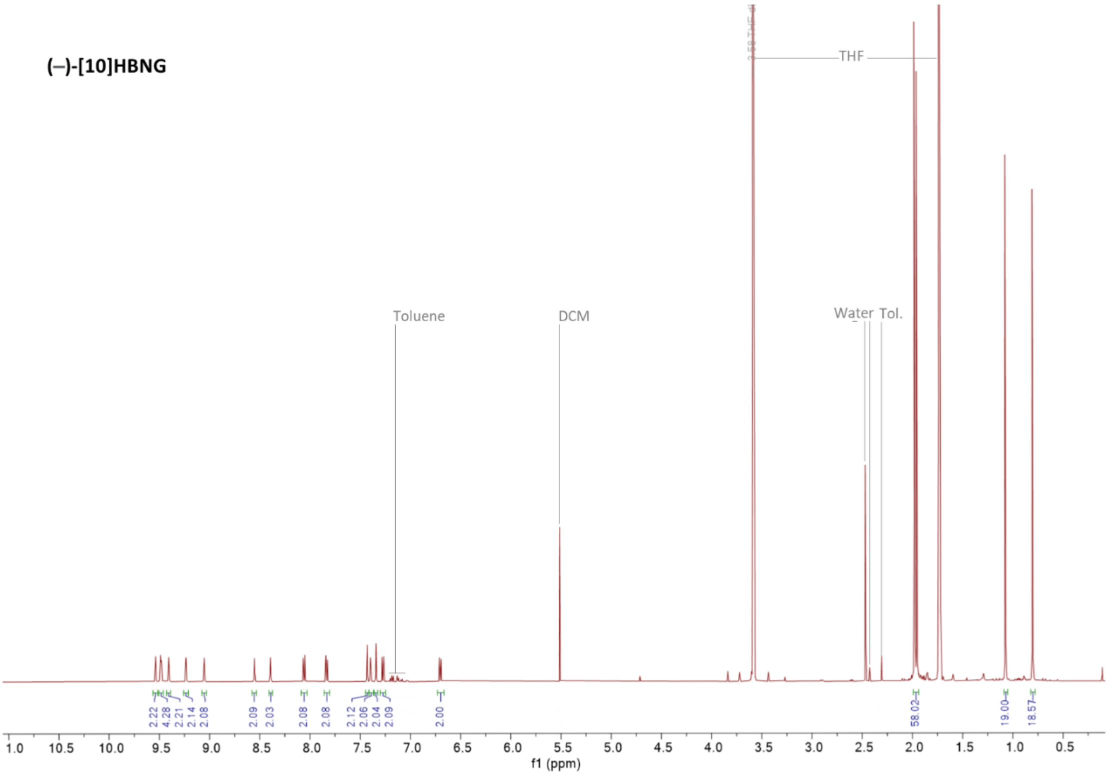
(–)-[10]HBNG
THF
Toluene
DCM
Water Tol.
2.22
4.28
2.21
2.14
2.08
2.09
2.03
2.08
2.08
2.12
2.06
2.04
2.09
2.00
58.02
19.00
18.57
f1 (ppm)

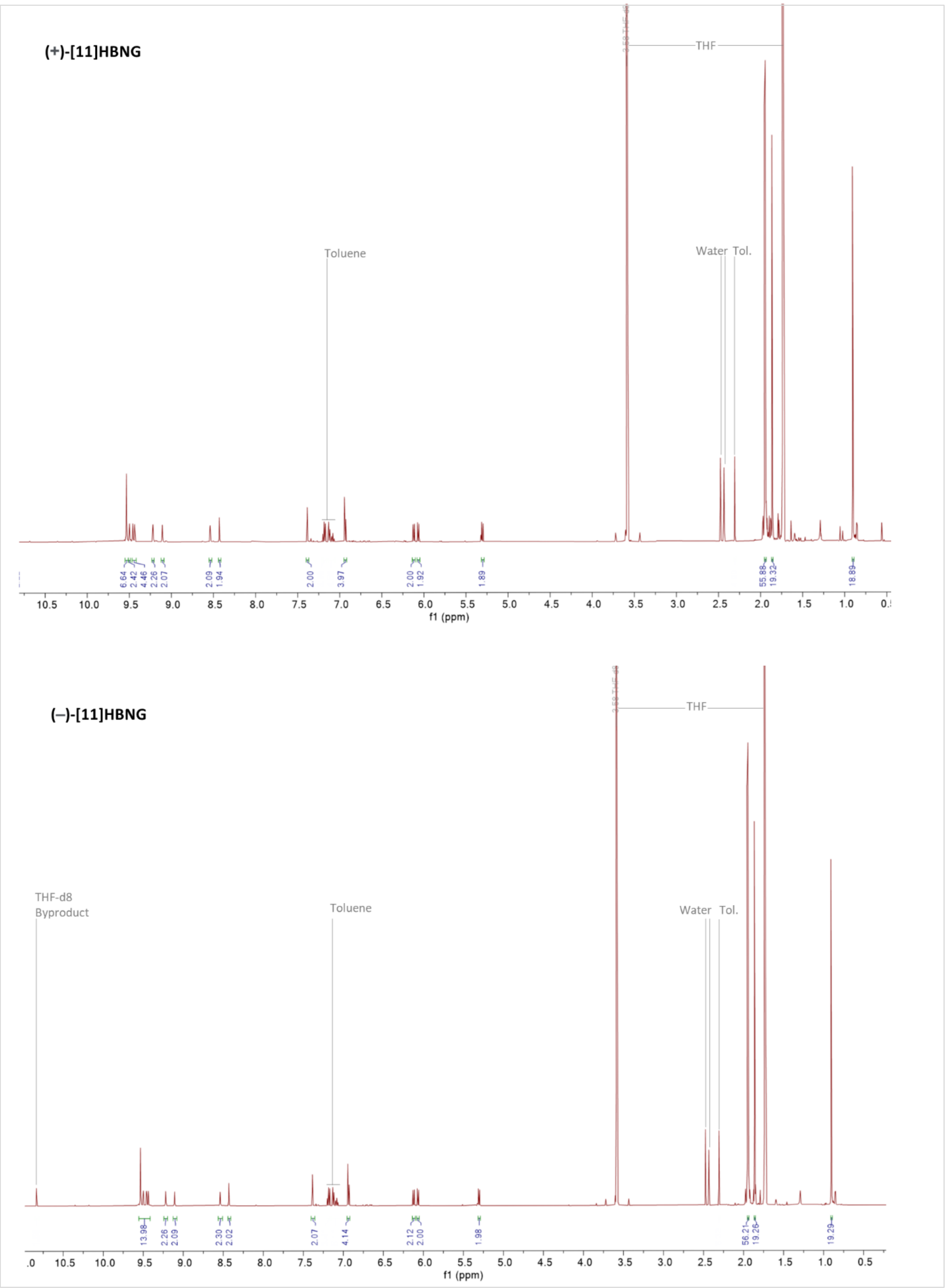

Figure S23. ^{1}H NMR spectra (in THF-d_8) of **[9]HBNG**, **[10]HBNG** and **[11]HBNG** as enantioenriched samples.

10. CD spectra

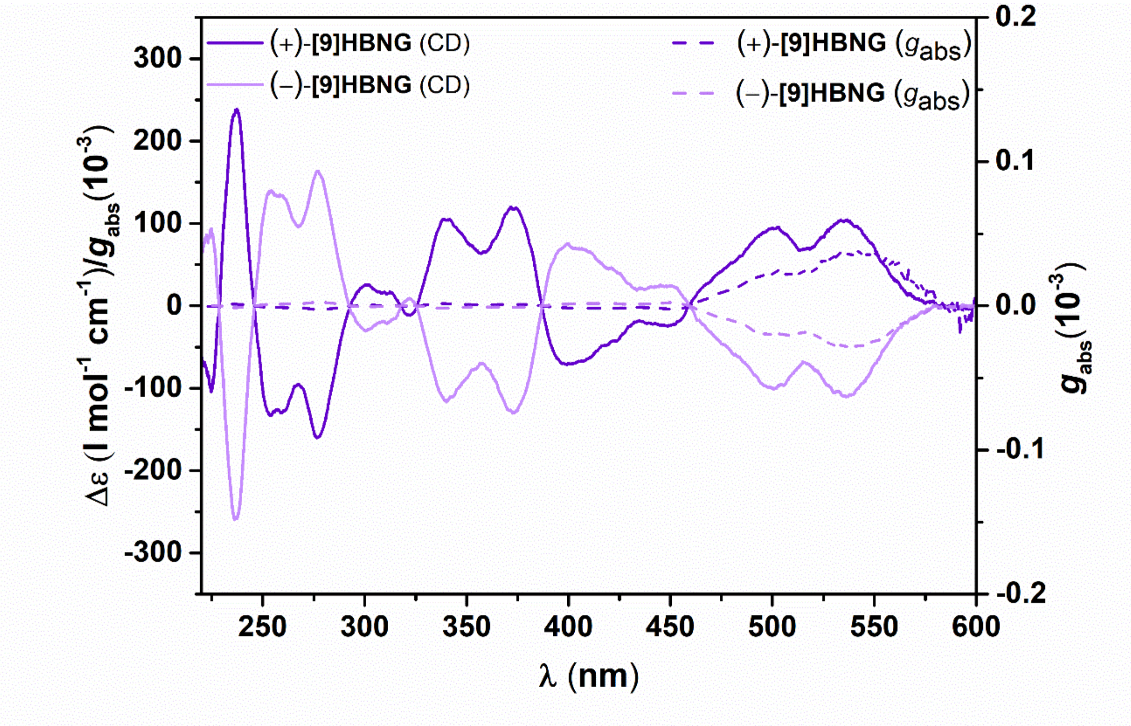

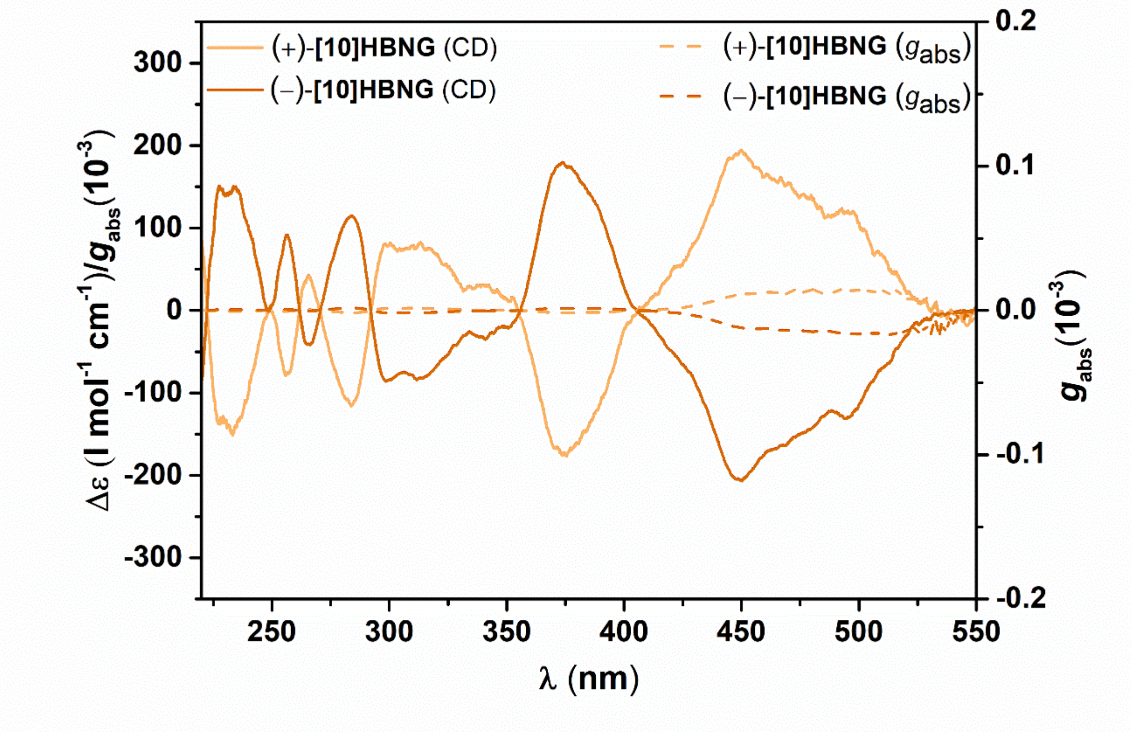

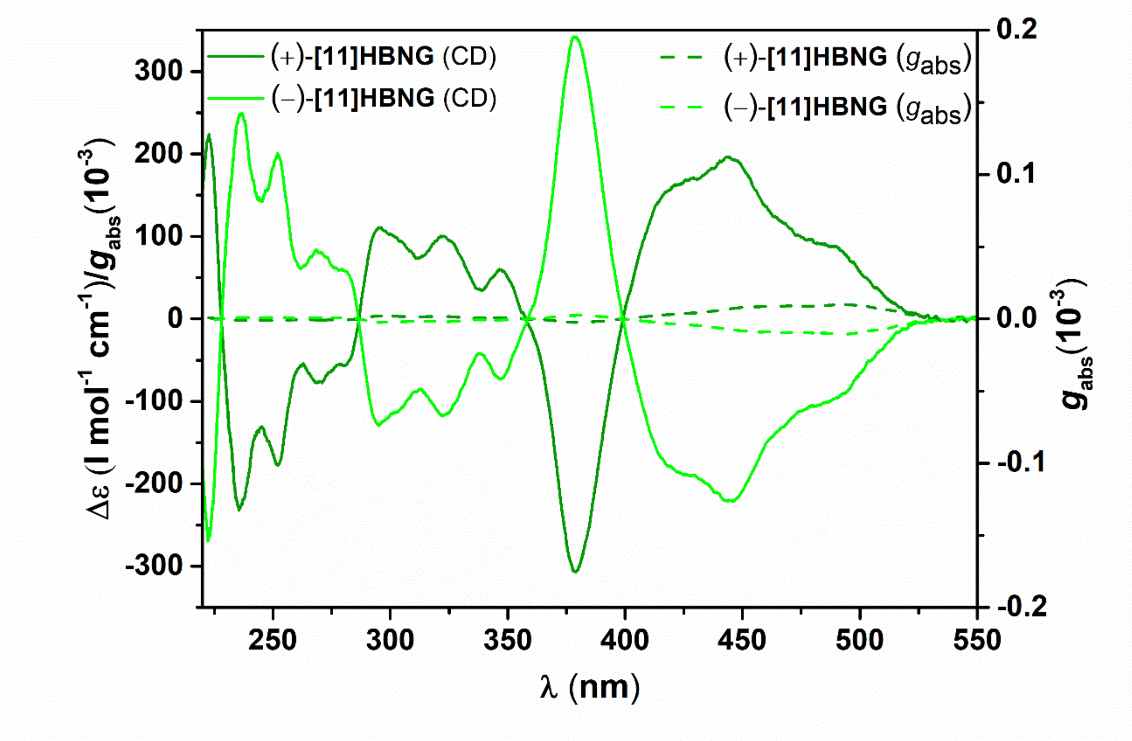

Figure S24. Optical spectra of **[9]HBNG**, **[10]HBNG** and **[11]HBNG** in THF, c = 2.5 × 10^{-5} M, l = 0.2 cm.*

Table S9. Values of g_{abs} for **[9]HBNG**, **[10]HBNG** and **[11]HBNG**.

Compound	**(+)-[9]HBNG**	**(-)-[9]HBNG**	**(+)-[10]HBNG**	**(-)-[10]HBNG**	**(+)-[11]HBNG**	**(-)-[11]HBNG**
g_{abs}	+3.6 × 10^{-2}	-2.8 × 10^{-2}	+1.4 × 10^{-2}	-1.6 × 10^{-2}	+1.0 × 10^{-2}	-1.0 × 10^{-2}

*The CD spectrum of **(+)-[10]HBNG** (including g_{abs} factor) is corrected to optical purity (*i.e.* scaled by a factor of ~1.4).

11. Quantum Chemistry Calculations

a)

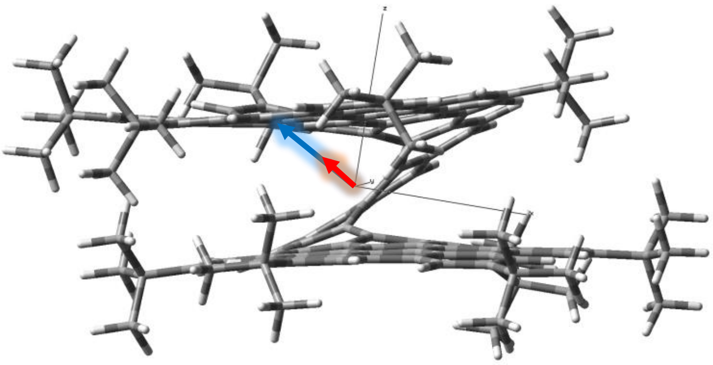

b)

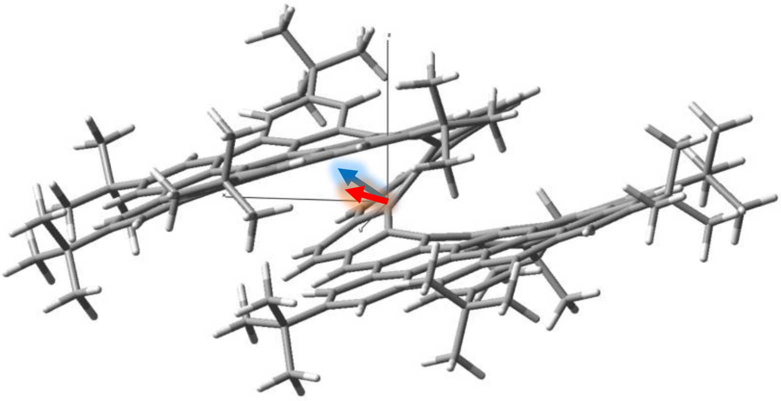

c)

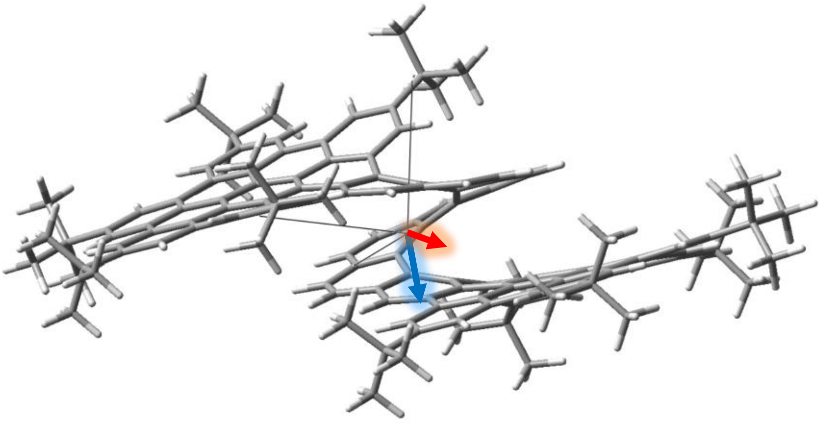

Figure S25. Direction and moduli of the ETDM (blue arrow) and MTDM (red arrows) of a) **[9]HBNG**, b) **[10]HBNG** and c) **[11]HBNG** calculated at the optimized CAM-B3LYP/3-21G* geometry.

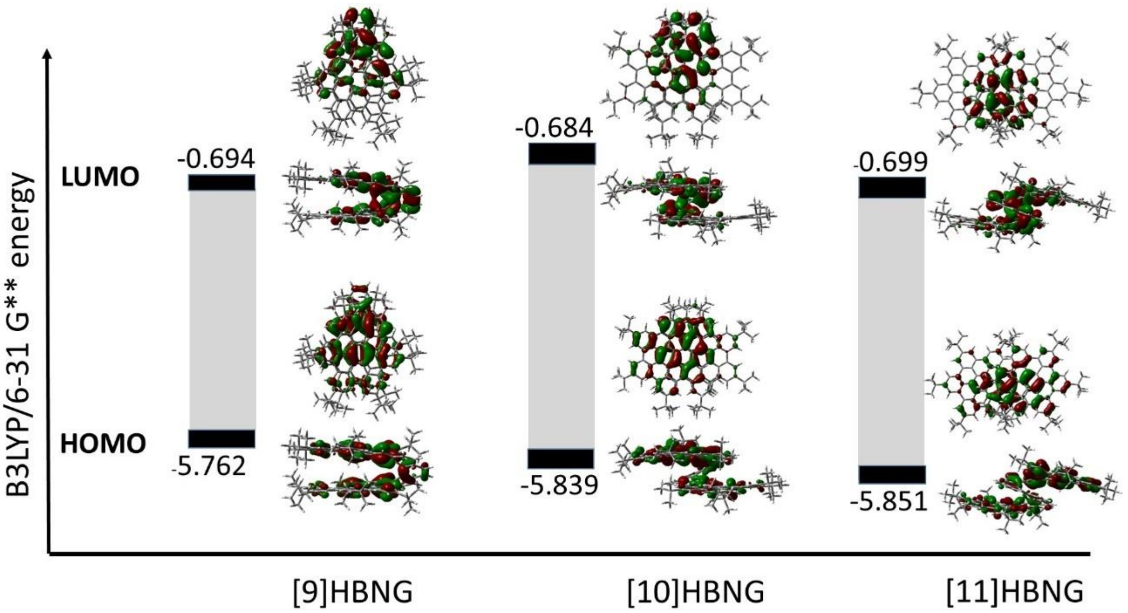

Figure S26. HOMO and LUMO energy calculations for [9]HBNG, [10]HBNG and [11]HBNG.
